# Calpain 2 promotes Lenvatinib resistance and cancer stem cell traits via both proteolysis-dependent and independent approach in hepatocellular carcinoma

**DOI:** 10.1186/s43556-024-00242-7

**Published:** 2024-12-31

**Authors:** Xiaolu Ma, Kaixia Zhou, Tianqing Yan, Ling Hu, Suhong Xie, Hui Zheng, Ying Tong, Heng Zhang, Yanchun Wang, Zhiyun Gong, Cuncun Chen, Yanan Tian, Lin Guo, Renquan Lu

**Affiliations:** 1 Department of Clinical Laboratory, Shanghai Cancer Center, Fudan University, Shanghai, 200032 China; 2 Department of Oncology, Shanghai Medical School, Fudan University, Shanghai, 200032 China

## Abstract

Lenvatinib, an approved first-line regimen, has been widely applied in hepatocellular carcinoma (HCC). However, clinical response towards Lenvatinib was limited, emphasizing the importance of understanding the underlying mechanism of its resistance. Herein, we employed integrated bioinformatic analysis to identify calpain-2 (CAPN2) as a novel key regulator for Lenvatinib resistance in HCC, and its expression greatly increased in both Lenvatinib-resistant HCC cell lines and clinical samples. Further in vitro and in vivo experiments indicated that knocking down CAPN2 greatly sensitized HCC cells to Lenvatinib treatment, while overexpression of CAPN2 achieved opposite effects in a Lenvatinib-sensitive HCC cell line. Interestingly, we observed a close relationship between CAPN2 expression and cancer stem cell (CSC) traits in HCC cells, evidenced by impaired sphere-forming and CSC-related marker expressions after CAPN2 knockdown, and *verse vice*. Mechanistically, we strikingly discovered that CAPN2 exerted its function by both enzyme-dependent and enzyme-independent manner simultaneously: activating β-Catenin signaling through its enzyme activity, and preventing GLI1/GLI2 degradation through direct binding to YWHAE in an enzyme-independent manner, which disrupting the association between YWHAE and GLI1/GLI2 to inhibit YWHAE-induced degradation of GLIs. Notably, further co-immunoprecipitation assays revealed that YWHAE could promote the protein stability of CAPN2 via recruiting a deubiquitinase COPS5 to prevent ubiquitination-induced degradation of CAPN2. In summary, our data demonstrated that CAPN2 promoted Lenvatinib resistance via both catalytic activity-dependent and -independent approaches. Reducing CAPN2 protein rather than inhibiting its activity might be a promising strategy to improve Lenvatinib treatment efficiency in HCC.

**Supplementary Information:**

The online version contains supplementary material available at 10.1186/s43556-024-00242-7.

## Introduction

Hepatocellular carcinoma (HCC) is currently the most common solid tumor worldwide. Its incidence has continued to escalate in recent years, especially in China, posing a great challenge to public health [1–3]. Though clinical advances have been achieved in recent years, including targeted therapy and systemic immunotherapy, the overall survival (OS) rate remains unsatisfactory with over 50% of patients dying within 5 years of initial therapy [4]. Hence, identification of key contributor for HCC development and progression, and the clarification of molecular mechanisms could help to improve prognosis.

Lenvatinib, a multitargeted tyrosine kinase inhibitor (TKI) which specifically targeting tyrosine receptor kinases including vascular endothelial growth factor receptors (VEGFRs), platelet-derived growth factor receptor (PDGFR) α, and fibroblast growth factor receptors (FGFRs), has been approved by the FDA as a first-line regimen for unresectable HCC patients [5]. Although the administration of Lenvatinib in clinical practice brings a new dawn for advanced HCC patients, the current clinical response remains unsatisfactory [6]. Hence, there is an urgent need for understanding how Lenvatinib resistance formed and exploring novel target for overcoming drug resistance. Unfortunately, identifying the key contributor for the Lenvatinib resistance remains a great challenge. Accumulating evidence has revealed that cellular heterogeneity exists in most solid tumors including HCC [7]. Cancer stem cells (CSCs) are a minor fraction of tumor cells which exhibit capacities such as self-renewal and differentiation [8]. More importantly, CSC traits are considered as the potential driving force for Lenvatinib resistance [9]. However, due to the complexity of regulatory network involved in CSCs, there still lacking effective target to inhibit CSC traits which helps to reverse Lenvatinib resistance.

Calpain-2 (CAPN2) is a major non-lysosomal protease member of the calpain family [10]. Increasing evidence have confirmed CAPN2 as a critical regulator of tumor progression [11–13], and high CAPN2 expression serves as a promising indicator for unfavorable outcomes [14–16]. Notably, we identified CAPN2 as a novel upstream regulator for β-Catenin signaling activation [17], a critical molecular event rendering CSCs traits for HCC [18]. However, the specific role of CAPN2 in promoting CSC traits and Lenvatinib resistance still needs further determination.

In present study, we systematically investigated the role of CAPN2 in regulating CSC traits in HCC, especially its role in Lenvatinib resistance. Importantly, we unveiled that, in addition to activating the β-Catenin signaling via classical enzyme activity approach, CAPN2 could also promote Hedgehog signaling activation through the non-enzyme approach, synergistically promoting the occurrence of Lenvatinib resistance in HCC. Moreover, detailed regulatory mechanism was further investigated.

## Results

### Identification of CAPN2 as the critical member of calpain family for regulating Lenvatinib resistance in HCC

**Fig. 1:**
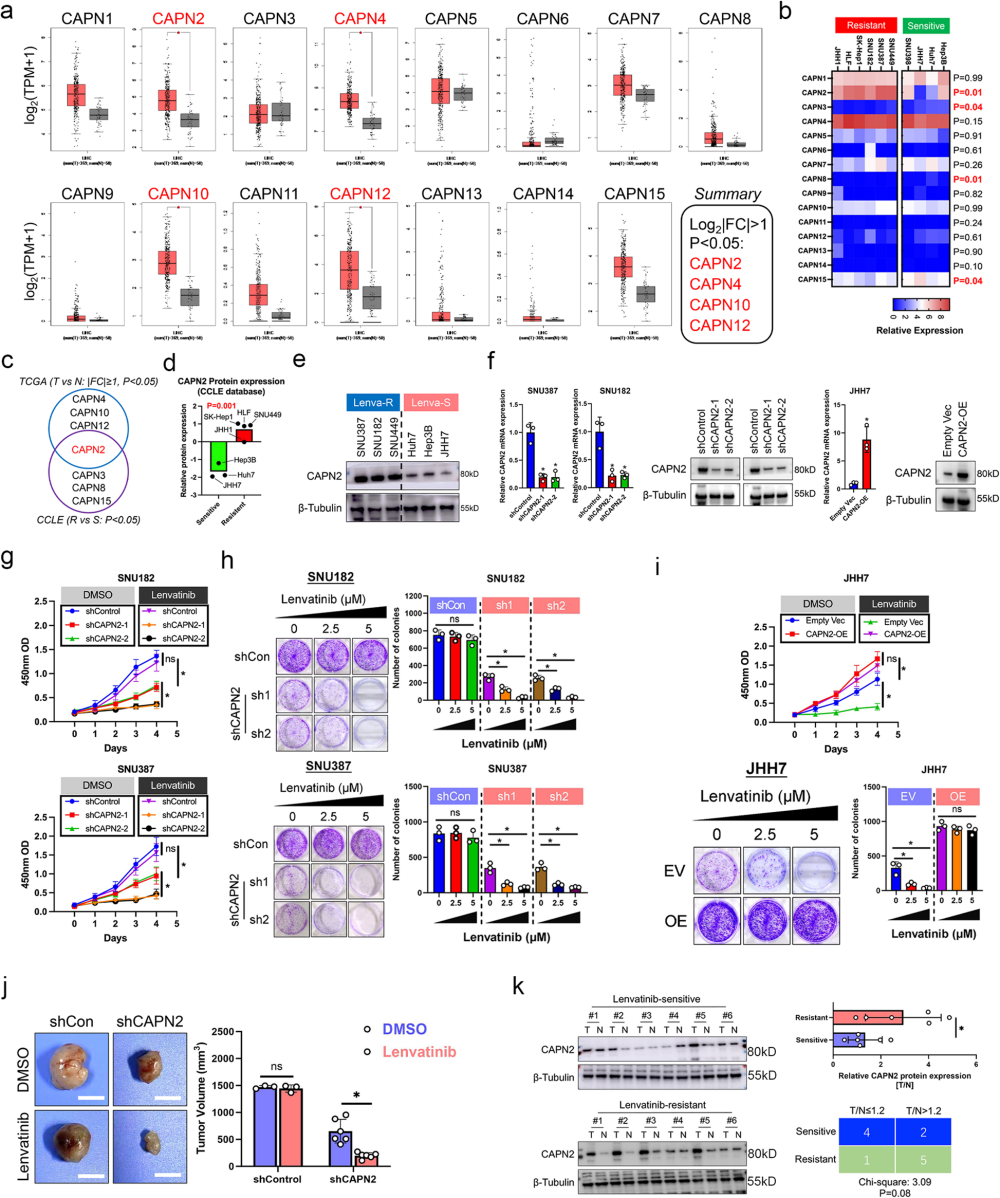
Identification of CAPN2 as the key member of calpain family to promote Lenvatinib resistance in HCC. **a** Expression patterns of each calpain family member between HCC and normal liver tissues according to TCGA dataset; Data were collected from GEPIA database. **b** Expression patterns of each calpain family member between Lenvatinib-resistant and Lenvatinib-sensitive HCC cell lines according to CCLE dataset. **c** Overlapped genes between the screening results of TCGA and CCLE dataset mentioned in (**a**) and (**b**). **d** Comparison of CAPN2 protein expression between indicated HCC cell lines according to CCLE dataset. **e** WB assay for detecting CAPN2 expression in indicated HCC cell lines. **f** PCR and immunoblotting assay results for verifying the CAPN2 knockdown efficiencies in indicated HCC cell lines. **g** CCK8 assay results for determining the effects of CAPN2 knockdown on Lenvatinib response in indicated HCC cell lines. **h** Colony formation assay results for determining the effects of CAPN2 knockdown on Lenvatinib response in indicated HCC cell lines. **i** CCK8 (upper) and colony formation (lower) assay results for determining the effects of CAPN2 overexpression on Lenvatinib response in indicated HCC cell lines. **j** In vivo results of CAPN2 knockdown on Lenvatinib response in SNU387 cells; for shControl group, 3 mice per group; for shCAPN2 group, 6 mice per group; Initially, 5 × 10^6^ cells per mouse were injected subcutaneously into the right posterior flanks of 6-week-old BALB/c nude mice; After tumor establishment, mice were randomly assigned to 5 days per week treatment with vehicle, Lenvatinib (4 mg kg − 1, oral gavage). **k** Immunoblotting assays results of CAPN2 protein expression in Lenvatinib-resistant and Lenvatinib-sensitive clinical samples. “ns” indicates no significance; “*” indicates *P* value less than 0.05

To identify the potential regulator for Lenvatinib resistance in calpain family, we first compared the expression distribution of all calpain family members between HCC and normal liver tissues according to TCGA dataset. Results showed that among all 15 calpain members, CAPN2, CAPN4, CAPN10 and CAPN12 exhibited significantly higher expression in HCC tissues according to follow criteria: Foldchange T/N ≥ 2, *P* < 0.05 (Fig. 1a). Next, we observed mRNA expression of calpain family members between Lenvatinib-resistant and Lenvatinib-sensitive HCC cell lines according to CCLE dataset. Specifically, response to Lenvatinib of HCC cell lines was determined by two previous published research [19, 20], and Hep3B, Huh7, JHH7 and SNU398 cell lines were identified as Lenvatinib-sensitive, whereas SNU449, SNU387, SNU182, SK-Hep1, HLF, and JHH1 cell lines were identified as Lenvatinib-resistant. Results showed that CAPN2 mRNA expressions were significantly elevated in Lenvatinib-resistant HCC cell lines, whereas CAPN3, CAPN8 and CAPN15 mRNA expressions were lower in Lenvatinib-resistant HCC cell lines (*P* < 0.05, Fig. 1b). Therefore, CAPN2 was identified as the only overlapped gene (Fig. 1c). Protein expression data from CCLE dataset and western blot (WB) assay further confirmed the elevated CAPN2 expression in Lenvatinib-resistant HCC cell lines (Fig. 1d and e). Hence, we speculated CAPN2 as the key member of calpain family that contributed to Lenvatinib resistance in HCC.

To further validate the crucial role of CAPN2 in mediating Lenvatinib resistance, we selected two resistant cell lines, SNU387 and SNU182, for CAPN2 knockdown. Meanwhile, a Lenvatinib sensitive cell line, JHH7, was chosen for CAPN2 overexpression due to the lowest CAPN2 expression. The knockdown and overexpression efficiencies were confirmed by RT-PCR and immunoblotting assays (Fig. 1f). Both cell counting-8 (CCK-8) and colony formation assays demonstrated that knocking down CAPN2 expression sensitized SNU387 and SNU182 cells to Lenvatinib treatment (all *P* < 0.05, Fig. 1g and h). Contrarily, overexpression of CAPN2 conferred resistance towards Lenvatinib treatment in JHH7 cells (all *P* < 0.05, Fig. 1i). Silencing CAPN2 also greatly enhanced treatment efficiency of Lenvatinib in vivo (Fig. 1j). Moreover, WB assays indicated that Lenvatinib-resistant patients exerted higher CAPN2 expression in tumoral tissue compared to paired adjacent liver tissues (Sensitive: low CAPN2 expression 4/6; Resistant: low CAPN2 expression 1/6, *P* = 0.06; Fig. 1k). Taken together, our data identified CAPN2 as a novel key member of calpain family for mediating Lenvatinib resistance in HCC.

### CAPN2 promoted cancer stem cell (CSC) traits of HCC cells

**Fig. 2:**
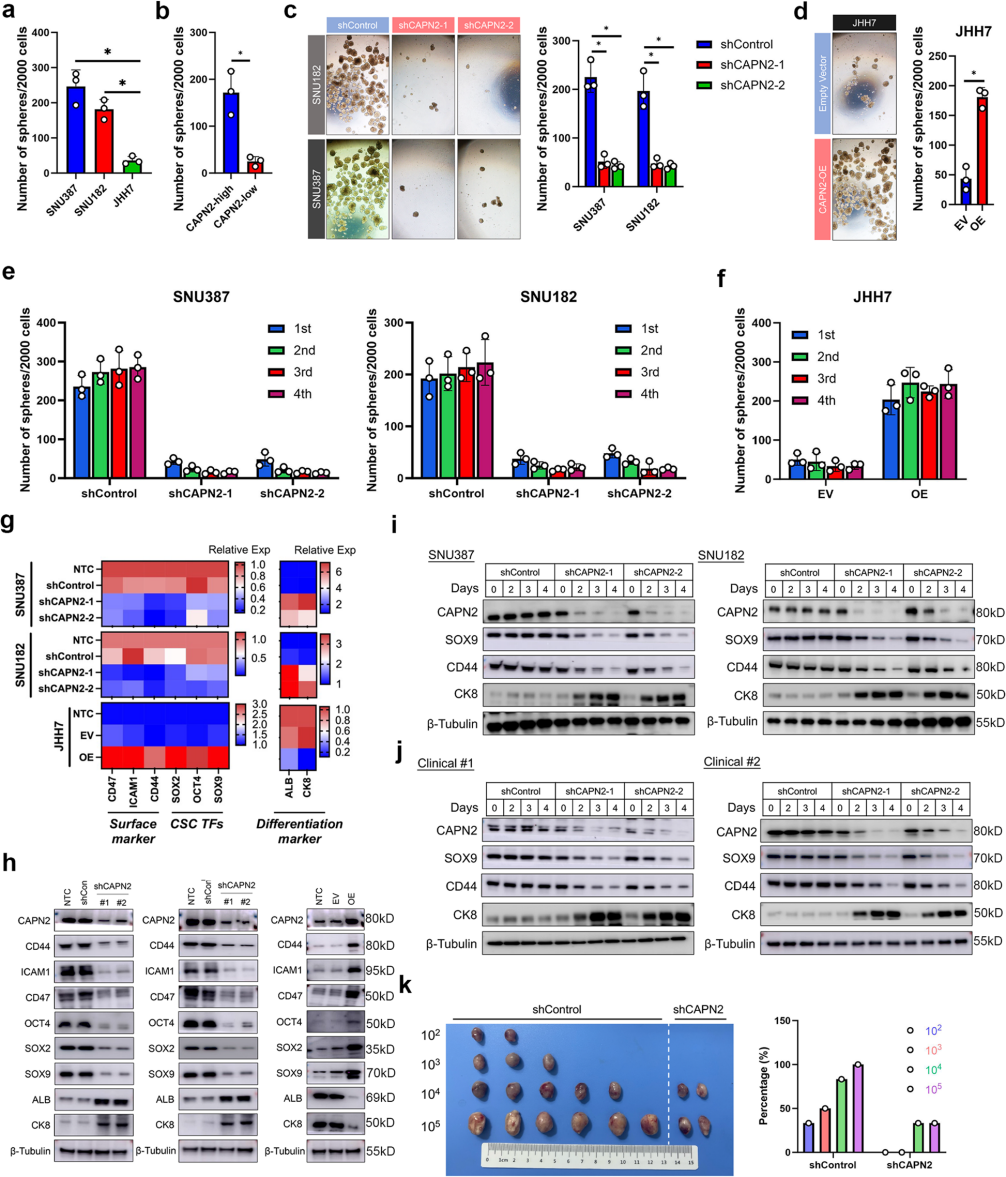
CAPN2 induced CSC traits in HCC. **a** Sphere-forming capacities of indicated HCC cell lines according to sphere-forming experiments. **b** Sphere-forming capacities of indicated cells derived from clinical samples according to sphere-forming experiments (CAPN2-high: 3 samples; CAPN2-low 3 samples). **c** Effects of CAPN2 knockdown on sphere-forming capacities of indicated HCC cell lines. **d** Effects of CAPN2 overexpression on sphere-forming capacities of JHH7 cells. **e** Effects of CAPN2 knockdown on serial sphere-forming capacities of indicated HCC cell lines. **f** Effects of CAPN2 overexpression on serial sphere-forming capacities of indicated HCC cell lines. **g** PCR assay results for the changes of indicated CSC-related marker and liver differentiation marker expressions upon CAPN2 expression alterations; experiments were conducted in triplicate. **h** WB assay results for the dynamic changes of indicated CSC-related marker and liver differentiation marker expressions upon CAPN2 knockdown (left two panel) or overexpression (right panel). **i** WB assay results for the dynamic changes of CSC-related marker and liver differentiation marker expressions after receiving shRNAs targeting CAPN2 in indicated cell lines. **j** WB assay results for the dynamic changes of CSC-related marker and liver differentiation marker expressions after receiving shRNAs targeting CAPN2 in sphere cells derived from indicated clinical samples. **k** Limiting dilution xenograft assay results of SNU387 cells received indicated treatment; Initially, indicated number of SNU387 cells per mouse were injected subcutaneously into the right posterior flanks of 6-week-old BALB/c nude mice (*n* = 6 per group), tumors were harvested 6 weeks after initial transplantation, and the frequence were calculated. “ns” indicates no significance; “*” indicates *P* value less than 0.05

CSC traits were considered as a typical traits of Lenvatinib-resistant HCC cells [9]. Interestingly, we found several stem cell-related molecular signatures were significantly enriched in CAPN2-high HCC in TCGA dataset according to gene set enrichment assay (GSEA, Fig. S1). Therefore, we hypothesized CAPN2 participated in the regulation of CSC traits in HCC. To verify this, we first conducted sphere-forming assays, a widely used in vitro approach for evaluating CSC traits [21]. Both CAPN2-high cell lines and primary HCC cells showed significantly stronger sphere formation capacities (both *P* < 0.05, Fig. 2a and b). Moreover, downregulating CAPN2 significantly hindered sphere formation in SNU182 and SNU387 cells (*P* < 0.05, Fig. 2c), whereas forced expression of CAPN2 achieve opposite result (*P* < 0.05, Fig. 2d). Further serial sphere formation assays indicated that CAPN2 knockdown markedly hindered the ability of cells to self-renew and verse vice (Fig. 2e and f). RT-PCR and WB assay results indicated that silencing CAPN2 significantly decreased expression of CSC-related marker, such as cluster of differentiation 44 (CD44), CD47, OCT4, SOX2, and SOX9, but increased the expressions of liver differentiation markers such as albumin (ALB) and cytokeratin 8 (CK8). Contrarily, overexpression of CAPN2 achieved opposite results in JHH7 cells (Fig. 2g and h). Notably, after transfection with CAPN2 shRNAs, the expression of SOX9 and CD44 decreased, while CK8 showed increased expression in a time-dependent manner in spheres derived from both CAPN2-high HCC cell lines and clinical samples (Fig. 2i and j). We further conducted limiting dilution xenograft assay, and found that CAPN2 downregulation significantly decreased tumor initiation (Fig. 2k). Together, above findings demonstrated the essential role of CAPN2 in maintaining CSC traits in HCC.

### CAPN2 partially relied on β-Catenin signaling to regulate Lenvatinib resistance and CSC traits in HCC

**Fig. 3:**
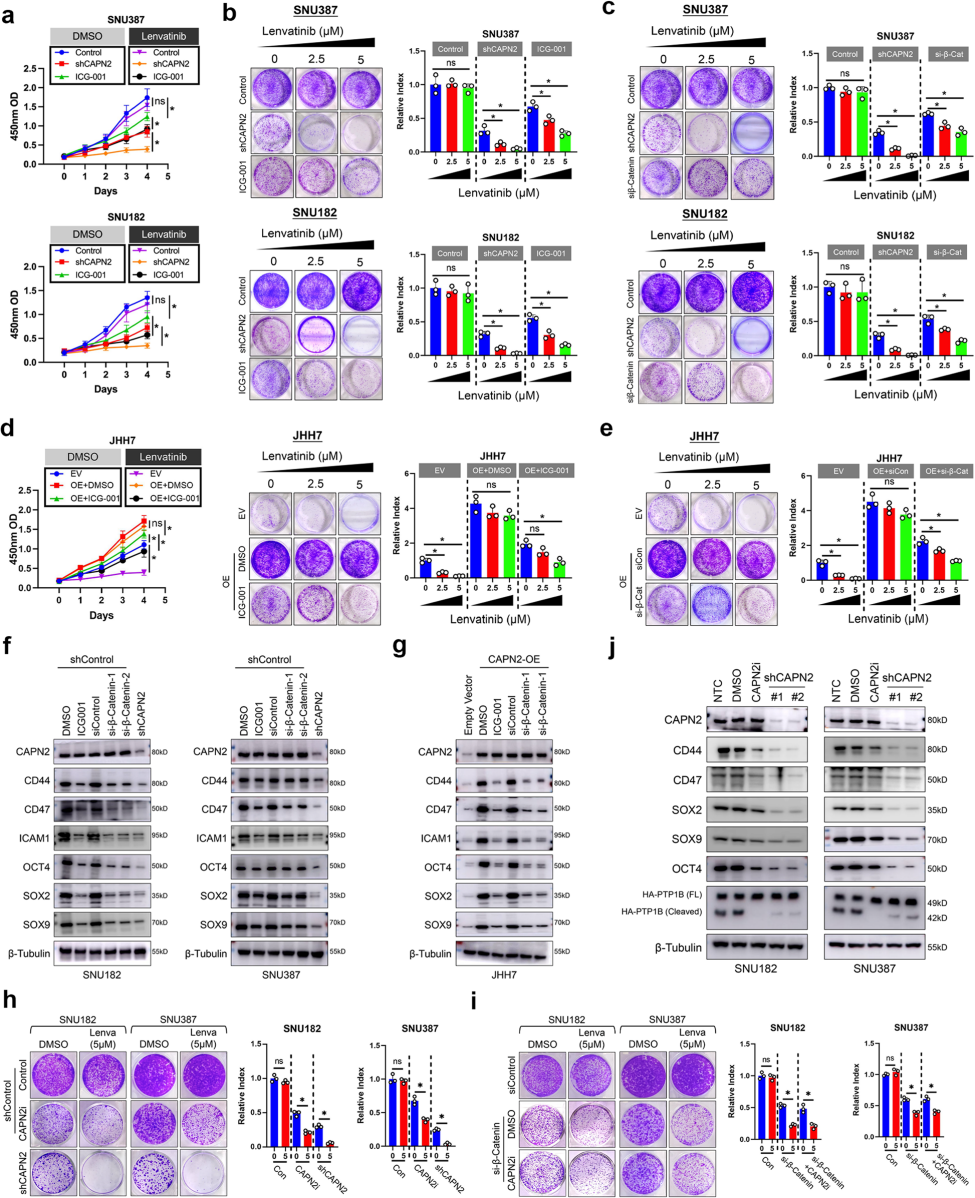
CAPN2 partially relied on β-Catenin signaling in an enzyme-dependent manner. **a** CCK8 assay results for determining the effects of β-Catenin signaling inhibition on Lenvatinib response of indicated HCC cell lines; CAPN2 knockdown was used as positive controls. **b** Colony formation assay results for determining the effects of β-Catenin signaling inhibition on Lenvatinib response of indicated HCC cell lines; CAPN2 knockdown was used as positive controls. **c** Colony formation assay results for determining the effects of silencing β-Catenin expression on Lenvatinib response of indicated HCC cell lines; CAPN2 knockdown was used as positive controls. **d** CCK8 (left) and colony formation (right) assay results for determining the effects of β-Catenin signaling inhibition on Lenvatinib response in CAPN2-overexpressed JHH7 cells. **e** Colony formation assay results for determining the effects of silencing β-Catenin expression on Lenvatinib response in CAPN2-overexpressed JHH7 cells. **f** WB assay results to evaluate the CSC-related expression alterations upon receiving indicated treatments in SNU182 (left) and SNU387 (right) cells. **g** WB assay results for evaluating CSC-related expression alterations upon receiving indicated treatments in JHH7 cells. **h** Comparison of inhibitory effects of SNU387 and SNU182 cells received indicated treatments; revealed weaker inhibitory effects of CAPN2 inhibitor than CAPN2 knockdown did. **i** Comparison of inhibitory effects of SNU387 and SNU182 cells received indicated treatments; revealed CAPN2 inhibitor exerted no further inhibitory effects when β-Catenin expression was silenced. **j** WB assay experiments for comparing the dynamic changes of CSC-related marker expression between CAPN2 knockdown and CAPN2 enzymatic inhibition. “ns” indicates no significance; “*” indicates *P* value less than 0.05

Previously, we reported CAPN2 could activate β-Catenin signaling in an enzyme-dependent manner [17]. Since β-Catenin signaling was reported to participate in target-therapy resistance in HCC [22], we investigated whether CAPN2 relied on β-Catenin signaling to promote Lenvatinib resistance. Both CCK8 and colony formation assays demonstrated that ICG-001, a specific antagonist against β-Catenin signaling, could modestly sensitize SNU182 and SNU387 cells to Lenvatinib treatment, which was weaker than the effects of CAPN2 knockdown (Fig. 3a and b). We also found silencing endogenous β-Catenin expression exerted similar effects as ICG-001 treatment did (Fig. 3c and Fig. S2a). More importantly, both pharmacological inhibition and silencing β-Catenin failed to completely abolish the promotional effects of CAPN2 overexpression in JHH7 cells (Fig. 3d and e). Meanwhile, we discovered that both ICG-001 treatment and β-Catenin silence only modestly reduced expressions of CSC-related marker in SNU182 and SNU387 cells (Fig. 3f, Fig. S2b). ICG-001 treatment or specific siRNAs targeting β-Catenin also failed to completely abolish the promotional effects of CAPN2 overexpression on CSC-related markers in JHH7 cells (Fig. 3g). These data indicated the partial function of β-Catenin signaling in CAPN2-mediated Lenvatinib resistance and CSC trait.

Next, we explored whether CAPN2 exerted its function via enzyme-dependent manner. Treatment with calpain inhibitor IV, a highly selective inhibitor for CAPN2, could only result in partial sensitization effects when compared to CAPN2 knockdown in SNU387 cells (Fig. 3h, Fig. S3a), which phenocopied the influence of β-Catenin signaling inhibition. Critically, calpain inhibitor IV showed no further sensitization effect when endogenous β-Catenin was silenced (Fig. 3i, Fig. S3b). Accordingly, calpain inhibitor IV only partially decreased CSC marker expressions in CAPN2-high HCC cell (Fig. 3j). In summary, these data demonstrated CAPN2 partially relied on β-Catenin signaling, which was enzyme-dependent, to regulate the CSC traits and Lenvatinib resistance.

### Hedgehog signaling participated in CAPN2-induced Lenvatinib resistance in HCC via an enzyme-independent approach

**Fig. 4:**
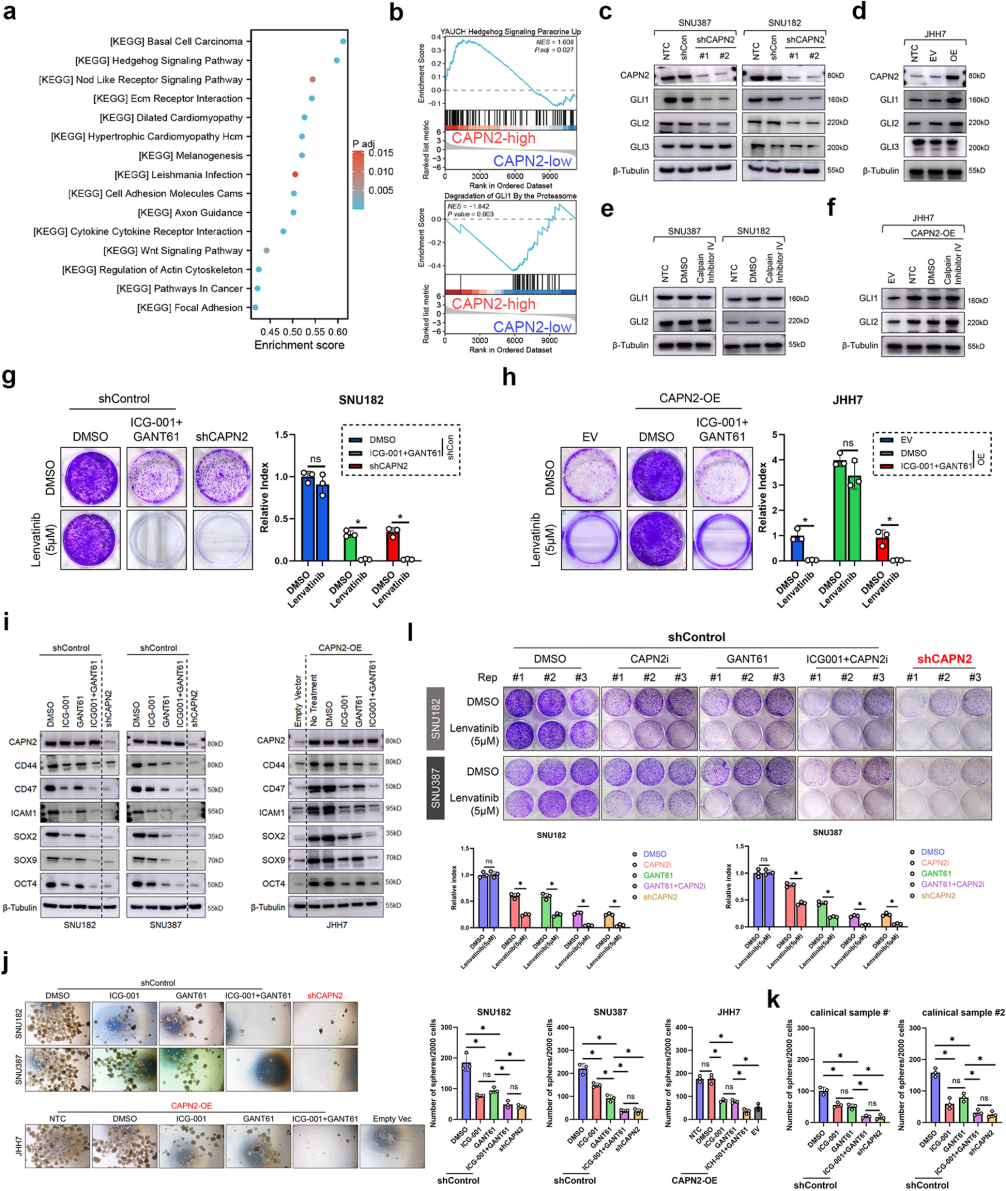
CAPN2 relied on Hedgehog signaling to promote Lenvatinib resistance in an enzyme-independent manner. **a** KEGG analysis revealed Hedgehog signaling as the most significant enriched pathway in CAPN2-high group in TCGA LIHC dataset. **b** Gene set enrichment analysis revealed Hedgehog signature was enriched in CAPN2-high group in TCGA LIHC dataset. **c** Effects of CAPN2 knockdown on the GLIs protein expressions according to WB assays. **d** Effects of CAPN2 overexpression on the GLIs protein expressions according to WB assays. **e** Effects of CAPN2 inhibitor on the GLIs protein expressions according to immunoblotting assays. **f** Effects of CAPN2 inhibitor on the GLIs protein expressions in CAPN2-overexpressed JHH7 cells according to WB assays. **g** Colony formation assays demonstrated the syngenetic effects of GANT61 and ICG-001 in sensitizing SNU387 cells to Lenvatinib. **h** Colony formation assays demonstrated the syngenetic effects of GANT61 and ICG-001 in abolishing the effects of CAPN2 overexpression on Lenvatinib in JHH7 cells. **i** WB experiment results confirmed the syngenetic effects of GANT61 and ICG-001 in decreasing CSC-related marker expressions in CAPN2-high (SNU182 and SNU387) and CAPN2-overexpressed JHH7 cells. **j** Sphere-forming assay results of HCC cell lines received indicated treatments; Representative images were shown in the left panel. **k** Sphere-forming assay results of HCC clinical samples received indicated treatments. **l** Colony formation assay for evaluating the potential syngenetic effects of GANT61 and CAPN2 inhibitor on reversing Lenvatinib resistance in CAPN2-high (SNU182 and SNU387) cells; Experiments were conducted in triplicate. “ns” indicates no significance; “*” indicates *P* value less than 0.05

To explore the downstream regulator of CAPN2 in addition to β-Catenin signaling, we re-investigated the KEGG pathways. GSEA results revealed Hedgehog signaling was listed as the most significant enriched pathway in CAPN2-high subpopulation concerning KEGG analysis (Fig. 4a). Moreover, we also observed YAUCH Hedgehog signature was positively enriched in CAPN2-high HCC, whereas Degradation of GLI1 by Proteasome signature was negatively correlated with CAPN2 high expression (Fig. 4b). Additionally, GSEA also demonstrated several Hedgehog-related molecular signatures enriched in CAPN2-high population (Fig. S4). We next detected the expression of GLI1-3, three key transcription factors involved in Hedgehog pathway, after CAPN2 expression modulation. Results showed that CAPN2 knockdown resulted in great reduction of GLI1 and GLI2 protein expression, whereas CAPN2 overexpression achieved the opposite effects (Fig. 4c and d). Importantly, CAPN2 inhibitor treatment shed no effect on GLI1 and GLI2 protein expression (Fig. 4e and f), indicating CAPN2 regulated GLI1/GLI2 expression in an enzyme-independent manner.

We further verified the function of Hedgehog signaling in CAPN2-promoted Lenvatinib resistance and CSC traits. We found GANT61, a GLI1/GLI2 specific antagonist, could synergize with ICG001 to sensitize CAPN2-high HCC cells towards Lenvatinib to the extent as CAPN2 knockdown did (Fig. 4g and h). Consistently, GANT61 cooperated with ICG-001 to downregulate the expression of CSC-related markers to a level similar to CAPN2 knockdown in CAPN2-high cells (Fig. 4i). Co-treatment with ICG-001 and GANT61 also completely abolished the promotional effects of CAPN2 overexpression in mediating CSC traits in JHH7 cells (Fig. 4i and j). Importantly, co-inhibition of β-Catenin and Hedgehog signaling also exerted similar effects in repressing sphere formation as CAPN2 knockdown did in two CAPN2-high clinical samples (Fig. 4k, Fig. S5). Unlike ICG-001, we found GANT61 treatment could effectively synergize with CAPN2 inhibitor to sensitize CAPN2-high HCC cells towards Lenvatinib (Fig. 4l). Further WB assay results indicated GANT61 cooperated with CAPN2 inhibitor to downregulating the expression of CSC-related markers to a level like CAPN2 knockdown did (Fig. S6). Collectively, above data demonstrated that Hedgehog signaling was complementary to β-Catenin signaling to entirely mediate CAPN2-induced Lenvatinib resistance in HCC.

### CAPN2 enhanced protein stability of GLI1 and GLI2 to sustain Hedgehog signaling activation

**Fig. 5:**
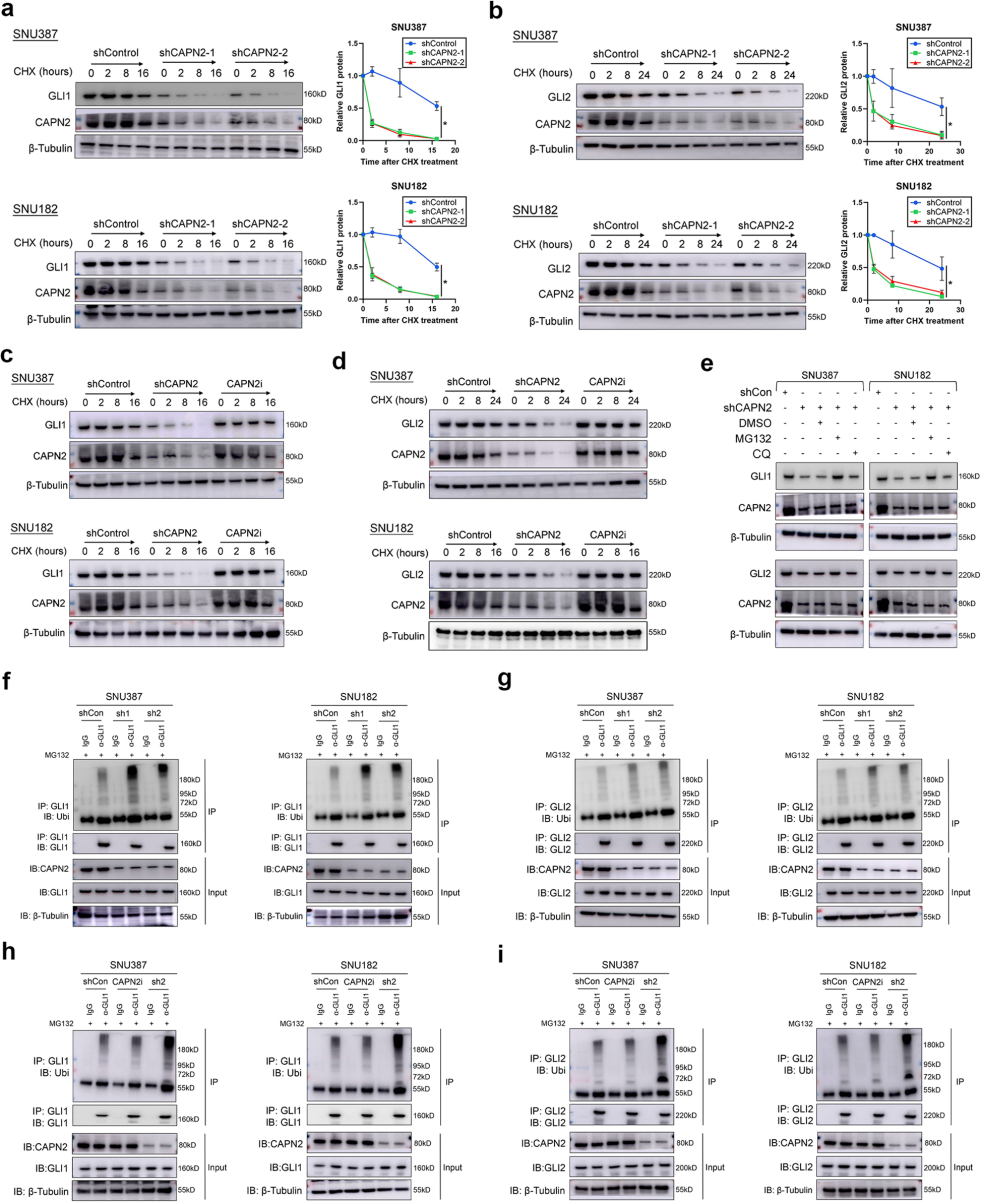
CAPN2 promoted Hedgehog signaling via preventing GLI1/2 proteasome degradation. **a** Half-life alterations of GLI1 protein upon CAPN2 knockdown were determined by CHX-chasing experiments. **b** Half-life alterations of GLI2 protein upon CAPN2 knockdown were determined by CHX-chasing experiments. **c** Half-life alterations of GLI1 protein upon CAPN2 inhibitor treatment were determined by CHX-chasing experiments. **d** Half-life alterations of GLI2 protein upon CAPN2 inhibitor treatment were determined by CHX-chasing experiments. **e** Recovery effects of proteasome pathway inhibitor, MG132, and lysosome pathway inhibitor, chloroquine, on GLI1/2 protein expressions in CAPN2 knockdown HCC cells were determined by immunoblotting assays. **f** Ubiquitination experiments for detecting the ubiquitination levels of GLI1 in HCC cells received indicated treatments. **g** Ubiquitination experiments for detecting the ubiquitination levels of GLI2 in HCC cells received indicated treatments. **h** Ubiquitination experiments for detecting the changes of ubiquitination levels of GLI1 in HCC cells received CAPN2 inhibitor and specific shRNA. **i** Ubiquitination experiments for detecting the changes of ubiquitination levels of GLI2 in HCC cells received CAPN2 inhibitor and specific shRNA. “ns” indicates no significance; “*” indicates *P* value less than 0.05

Considering the importance of GLI1 and GLI2 in mediating Hh signaling function, we next aimed to explore how CAPN2 regulated GLI1 and GLI2 expressions. First, we observed the mRNA expression of GLI1 and GLI2 remained unaltered after CAPN2 knockdown or overexpression (Fig. S7), inspiring us to prompt the hypothesis that CAPN2 improved GLI1/GLI2 expression at post-transcriptional level. CHX chasing results showed that knocking down CAPN2 significantly reduced the stability of GLI1 and GLI2 protein (Fig. 5a and 5b), however, specific inhibiting CAPN2 catalytic capacity failed to alter the protein stability of GLI1 and GLI2 (Fig. 5c and d). Moreover, impairment of the GLI1 and GLI2 protein stability caused by CAPN2 knockdown could be entirely recovered by MG132 (a proteasome-pathway inhibitor) but not chloroquine (a lysosome-pathway inhibitor, Fig. 5e), implying a critical role for the proteasome pathway in GLI1 and GLI2 protein degradation. Supportively, CAPN2 knockdown increased both GLI1 and GLI2 ubiquitination (Fig. 5f and g), whereas CAPN2 inhibitor treatment failed to increase the ubiquitination levels of GLI1 and GLI2 (Fig. 5h and i). Together, these data demonstrated CAPN2 mainly promoted Hedgehog signaling activation by preventing the ubiquitination-mediated proteasomal degradation of GLI1 and GLI2 protein in an enzyme-independent manner.

### CAPN2 interacted with YWHAE to suppress YWHAE-mediated GLIs protein degradation

**Fig. 6:**
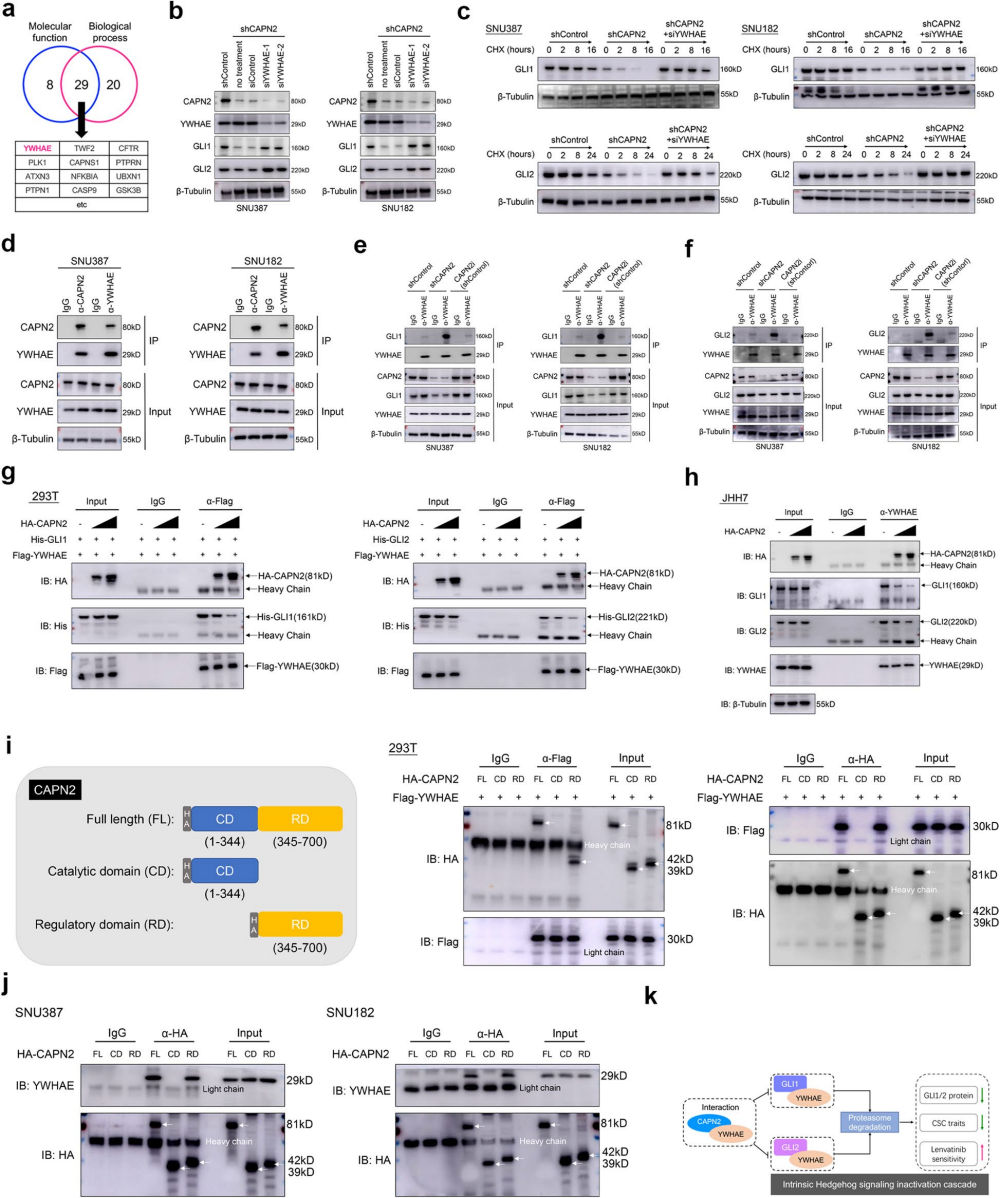
CAPN2 binds to YWHAE to prevent YWHAE-GLI1/2 interaction and following GLI1/2 degradation. **a** Candidates of CAPN2 binding partners according to PINA 3.0 dataset; a typical negative GLI1, GLI2 regulator, YWHAE, was listed. **b** WB assay results of GLI1 and GLI2 protein upon silencing YWHAE in CAPN2-knockdown HCC cells. **C** Half-life span changes of GLI1 and GLI2 protein upon receiving indicated treatments in HCC cells were determined by CHX chasing experiments. **d** Co-IP assays confirmed the interaction between CAPN2 and YWHAE. **e** Co-IP assays revealed CAPN2 knockdown promote YWHAE-GLI1 interaction, while CAPN2 inhibitor shed no effects on this interaction in HCC cells. **f** Co-IP assays revealed CAPN2 knockdown promoted YWHAE-GLI2 interaction, while CAPN2 inhibitor shed no effects on this interaction in HCC cells. **g** Co-transfection of HA-tagged CAPN2, His-tagged Gli1 and Flag-tagged YWHAE in 293 T cells followed by co-IP using anti-Flag antibodies to determine the effects of CAPN2 on YWHAE-GLI1 and GLI2 interactions; Cells were pre-treated with MG132 for 5 h to avoid GLI1/2 degradation. **h** Exogenous expression of different levels of CAPN2 in JHH7 cells followed by co-IP experiments to determine the effects of CAPN2 on YWHAE-GLI1 and GLI2 interactions; Cells were pre-treated with MG132 for 5 h to avoid GLI1/2 degradation. **i** Schematic diagram of CAPN2 protein domains (left) and Co-IP experiments for evaluating the critical domain responsible for the interaction between CAPN2 and YWHAE. **j** Co-IP experiments for validating the critical domain responsible for the interaction between CAPN2 and YWHAE in HCC cells. **k** Schematic diagram of CAPN2-mediated Hedgehog signaling activation in enzyme-independent manner

We further aim to explore how CAPN2 prevented GLI1 and GLI2 protein degradation. Based on PINA 3.0 database [23], 37 interactors of CAPN2 were identified by molecular function analysis (Cutoff = 0.7), while 49 interactors were identified by biological process enrichment (Cutoff = 0.7). A total of 29 overlapped genes were identified as potential candidates. Interestingly, we noticed YWHAE, a previously reported negative regulator for GLI1 and GLI2 [24], was included in the candidate list (Fig. 6a). Hence, we raised the hypothesis that CAPN2 might contribute to GLI1 and GLI2 expression via binding to YWHAE. To validate this notion, we first silenced YWHAE via siRNA in CAPN2-knockdown HCC cells. Results showed restored GLI1 and GLI2 protein expressions after YWHAE silence (Fig. 6b). Moreover, protein stability of GLI1 and GLI2 was effectively recovered by silencing YWHAE (Fig. 6c). These findings confirmed that CAPN2 regulated GLI1/GLI2 protein expression in a YWHAE-dependent manner.

We next determined how CAPN2 impaired YWHAE-induced GLI1/GLI2 degradation. As previous study reported, YWHAE inhibited GLI1 and GLI2 via direct interaction, we speculated that CAPN2 might bind to YWHAE to weaken the interaction between GLI1/GLI2 and YWHAE, resulting in their increased expression. Co-immunoprecipitation (Co-IP) analysis revealed endogenous interactions between CAPN2 and YWHAE in HCC cells (Fig. 6d). Moreover, CAPN2 knockdown greatly enhanced the interaction between YWHAE and GLI1/GLI2, while CAPN2 inhibitor shed no effects on their interaction (Fig. 6e and f), indicating the non-enzymatic activity dependence. Critically, further Co-IP experiments revealed that CAPN2 could compete with GLI1/GLI2 to bind to YWHAE in a dose-dependent manner in both 293 T and JHH7 cells (Fig. 6g and h).

We next determined the critical domain of CAPN2 for binding YWHAE by co-expressing different HA-tagged fragments of CAPN2 and Flag-tagged YWHAE in 293 T cells, followed by Co-IP assay. We discovered that a C-terminal fragment (termed regulatory domain [RD]) could bind to YWHAE (Fig. 6i). Moreover, exogenous full-length or RD region of CAPN2 could also co-precipitated with endogenous YWHAE in HCC cells (Fig. 6j). Collectively, our data demonstrated the important role of CAPN2 in preventing YWHAE-induced GLI1 and GLI2 ubiquitination degradation by disrupting YWHAE-GLI1/GLI2 interaction, and this regulatory effect was indispensable of the catalytic activity of CAPN2 (Fig. 6k).

### YWHAE promoted the protein stability of CAPN2 via inducing CAPN2-COPS5 interaction

**Fig. 7:**
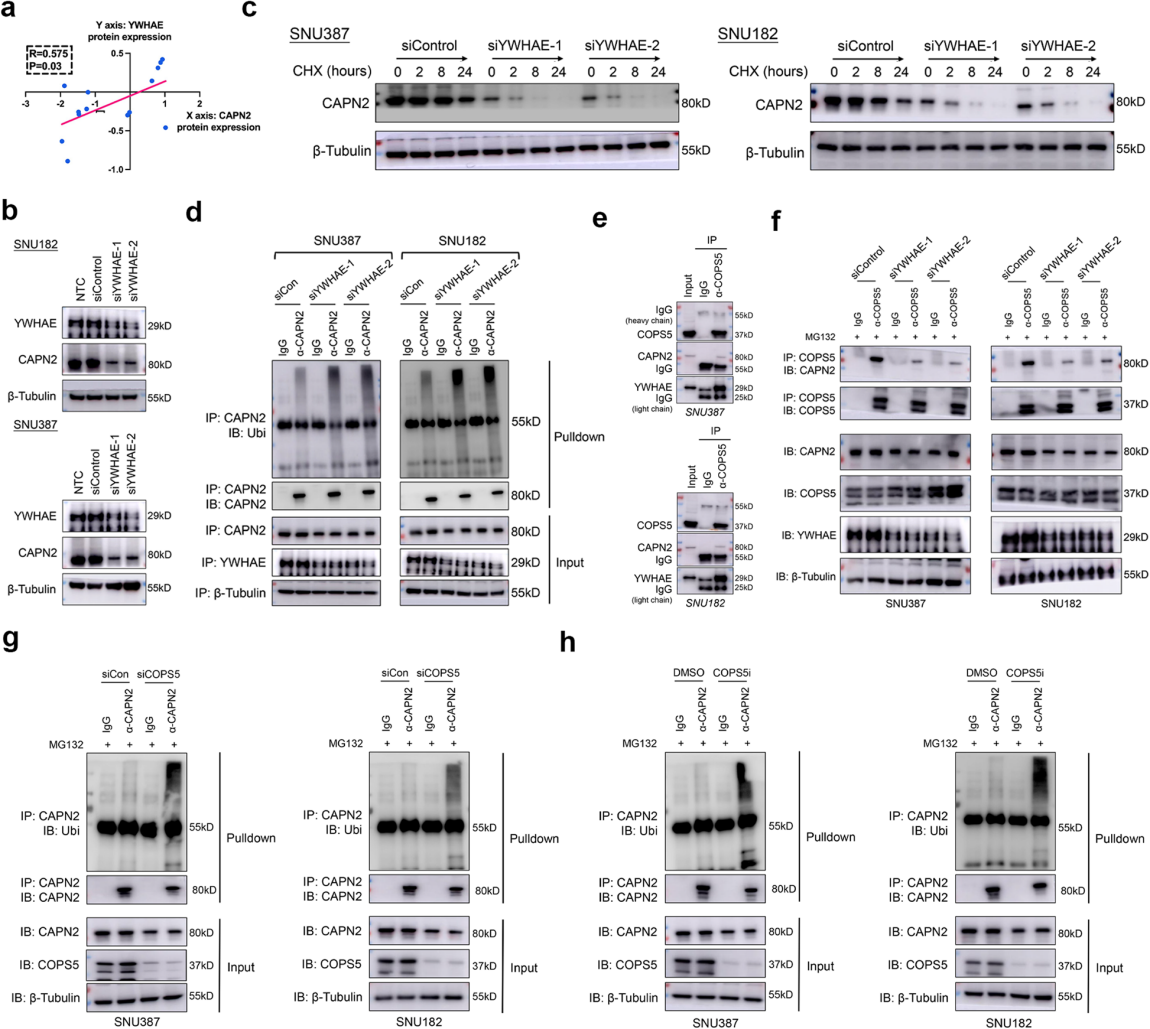
YWHAE binding CAPN2 to promote its protein stability via recruiting COPS5. **a** Positive correlation between YWHAE and CAPN2 proteins according to CCLE dataset. **b** Effects of YWHAE knockdown on CAPN2 expression in SNU182 (upper) and SNU387 (lower) cells. **c** Half-life span of CAPN2 protein upon YWHAE knockdown in HCC cells was determined by CHX chasing experiments. **d** Ubiquitination experiments for detecting the ubiquitination levels of CAPN2 in HCC cells received indicated treatments. **e** Co-IP experiments indicated interaction among COPS5, CAPN2 and YWHAE in SNU182 and SNU387 cells; endogenous protein was immunoprecipitated via using anti-COPS5 antibody, followed by WB assays. **f** Co-IP experiments indicated silencing YWHAE abolished COPS5-CAPN2 interaction. **g** Ubiquitination experiments for detecting the ubiquitination levels of CAPN2 in HCC cells upon COPS5 knockdown. **h** Ubiquitination experiments for detecting the ubiquitination levels of CAPN2 in HCC cells upon COPS5 inhibition

Intriguingly, we found CAPN2 protein expression was positively correlated with YWHAE protein expression in liver cancer cell lines according to CCLE database (Fig. 7a). Moreover, we observed marked reduction of CAPN2 protein after YWHAE knockdown in SNU387 and SNU182 cells (Fig. 7b), while thier mRNA expression remained unaltered (Fig. S8). Further CHX chasing experiments also demonstrated that knockdown of YWHAE resulted in a decreased half lifespan of CAPN2 protein (Fig. 7c). Contrarily, silencing YWHAE expression greatly increased the ubiquitination level of CAPN2 (Fig. 7d). To further elucidate how YWHAE-CAPN2 interaction prevented CAPN2 protein ubiquitination, we further investigated the interactor of CAPN2 according to BioGRID database, and mainly focused on the deubiquitinase. We noticed that COPS5, a well-established deubiquitinase in HCC [25], was identified as an interactor of CAPN2. Co-IP assays confirmed that COPS5 could bind to CAPN2 and YWHAE (Fig. 7e). More importantly, silencing YWHAE greatly hindered COPS5-CAPN2 interaction (Fig. 7f). Meanwhile, both knocking down COPS5 and applying specific inhibitor of COPS5 markedly increased CAPN2 protein ubiquitination level (Fig. 7g and h). Together, our data indicated YWHAE was essential for CAPN2 protein expression by recruiting COPS5 to CAPN2, a process that prevented CAPN2 protein ubiquitination and following degradation.

## Discussion

Lenvatinib was approved as a target-therapy regimen for the first-line therapy in advanced HCC. According to the results of clinical trial, Lenvatinib effectively improve the prognosis such as disease-free progression [6]. Unfortunately, resistance to Lenvatinib is frequently observed, which greatly restricts the therapeutic advantages [22]. Among several factors that contribute to the resistance of Lenvatinib, CSC traits are often mentioned and are considered as one of most significant characteristics [26, 27].

Here, we report CAPN2 as a novel mediator for CSC traits in HCC. Functional experiments characterized that CAPN2 expression was essential for the capacities of self-renewal, differentiation. Further investigations indicated that CAPN2 promoted CSC traits through dual signaling mechanisms: activating β-Catenin signaling via an enzyme-dependent manner, meanwhile, preventing GLI1 and GLI2 degradation by inhibiting GLI1 or GLI2-YWHAE interaction in an enzyme-independent manner. It should be noted that our results highlighted the non-catalytic of CAPN2, a new function of CAPN2, which provided novel insight for understanding the complicated function of calpain family. Clinically, we also showed that patients with low CAPN2 expression were prone to respond to Lenvatinib treatment, which provided a novel biomarker for the early warning of Lenvatinib resistance. Moreover, our results suggested that detecting expression level of CAPN2 before initiating Lenvatinib treatment might be a promising approach to avoid treatment failure, especially for the patients whose tumoral samples are available (resected or needle biopsy). For the patients whose tumoral samples are unavailable, we are exploring reliable circulating biomarkers for evaluating the expression level of CAPN2 in tumor lesions. On the other hand, for those who suffer resistance during Lenvatinib treatment, our results raised the opinion that inventing clinical available specific inhibitor for β-Catenin and Hedgehog signaling might be helpful in CAPN2-high subgroup.

As a major member of the calpain family, CAPN2 was mostly reported to exert its regulatory function via catalyzing substrates [11]. In our previous study, we also characterized CAPN2 as a novel oncogenic molecule promoting HCC progression by activating β-Catenin signaling through catalyzing PTP1B truncation [17]. Similarly, we also confirmed that the CAPN2-induced β-Catenin signaling activation participated in the CSC traits and Lenvatinib resistance. Unexpectedly, we found that CAPN2 only partially relied on activating β-Catenin signaling. More importantly, the remaining function of CAPN2 did not depend on enzyme activity, but relied on its binding to YWHAE, thus restraining the interaction between GLI1/GLI2 and YWHAE. Detailed analysis revealed the non-catalytic region within the protein sequence was responsible for the binding to YWHAE. Our results demonstrated a dual mechanism for CAPN2-induced Lenvatinib resistance. It should be noted that, based on our findings, only targeting the catalytic activity by highly selective antagonist might not be a successful strategy to control CAPN2-induced disease progression in the context of target therapy. Instead, searching for upstream regulatory mechanism which is responsible for high expression of CAPN2 in HCC might be helpful. In addition, with development of target protein degradation, such as PROTAC technology, inducing CAPN2 protein degradation might be promising approach in the future.

Aberrant activation of β-Catenin signaling is a critical molecular event during the dedifferentiation of HCC cells [28, 29]. Importantly, previous activated β-Catenin signaling could trigger Lenvatinib resistance by following approaches: 1) enhancing CSC markers expression, such as CD44 [30]; 2) activating downstream kinase network, such as MEK/ERK [22]; 3) inducing GPX2 expression to prevent cell apoptosis [31]. Previously, we discovered CAPN2 was a upstream promotor for β-Catenin signaling activation [17]. In present study, we also found CAPN2 relied on this approach to regulate the β-Catenin signaling, as the selective inhibitor targeting CAPN2 could effectively restrain β-Catenin activation. These data strengthen the notion that CAPN2 served as a critical upstream regulator for β-Catenin signaling in HCC. It should also be noted that the downstream regulatory network of β-Catenin which contributed to Lenvatinib resistance in HCC still needed to be more detailed clarified, and this work is undergoing in our lab currently.

On the other hand, Hodgehog signaling is critical the regulation of embryonic development, thus rendering its potential in promoting CSC traits [32]. Importantly, activation of Hodgehog signaling was essential for the tumorigenesis and progression of various types of cancers including HCC [33]. Activated GLIs could serve as powerful transcription factor to induce stem cell-related molecules expression, a critical regulatory process for sustaining CSC traits in HCC [34]. Generally, there are three GLIs in mammary, including GLI1, GLI2, and GLI3 [35]. In present study, we identified GLI1 and GLI2 as the key downstream effector of CAPN2. We further clarify CAPN2 promoted GLI1 and GLI2 protein stability in an enzyme-independent manner. As pilot study pointed out that, while Hedgehog signaling endowed cells with CSC characteristics, it also induced cells to enter a dormant state, which is not suitable to tumor dissemination. Therefore, the progression of tumor might require another “hit” in addition to Hedgehog pathway [36]. In context of our story, we posted that β-Catenin and Hedgehog signaling cooperate with each other and jointly participate in CAPN2-induced CSC characteristics and Lenvatinib resistance. Notably, our data demonstrated that simply targeting a single pathway might also not be a satisfactory strategy for reversing Lenvatinib resistance in HCC.

YWHAE, also known as 14–3-3ε, is an important member of 14–3-3 protein family [37, 38]. The exact role of YWHAE in HCC remains contradictory. In one hand, YWHAE could promotes epithelial-mesenchymal transition and was associated with the metastasis risk of HCC patients [39]. On the other hand, YWHAE was reported to be a critical negative regulator for Hedgehog signaling [24], and YWHAE-TAK1 association could inhibit the anti-apoptotic potentials of TAK1, resulting in apoptosis under Bleomycin treatment [40]. We inferred that the role of YWHAE was mainly dependent on its binding partner. In our study, we found CAPN2 could bind to YWHAE in an enzyme-independent manner, leading to the disruption of GLI1 or GLI2-YWHAE interaction. Our data revealed a novel regulatory role of YWHAE in HCC.

Since present study was conducted retrospectively, follow limitations should be noted when applying the discoveries of present study: 1) Bias in patient enrollment: we only collected 12 recurrent patients who received Lenvatinib treatment for the clinical value evaluation, therefore, the predictive significance of CAPN2 for Lenvatinib resistance in unresectable HCC patient cohort needs to be further validated; 2) Follow-up time is relatively short, and overall survival information is not provided, which would inevitably lead to bias when interpreting clinical significance of CAPN2, especially for the long-term treatment response evaluation; 3) Individual heterogeneity as well as intratumoral heterogeneity shed great effect on treatment response to Lenvatinib in HCC, thus, more detailed analysis about the heterogeneity of CAPN2 in HCC at single-cell scale might provide more accurate information for evaluating Lenvatinib response. Therefore, the predictive value of CAPN2 for Lenvatinib resistance needs to be further validated by a large-cohort, prospective study containing advanced HCC patients with different baseline states. Moreover, the regulatory role of CAPN2 needs to be verified in patient derived models such as patient-derived organoids (PDO) and patient-derived xenografts (PDX), and these explorations are undergoing in our lab. Finally, detailed molecular explanation for why YWHAE binding to CAPN2 prior to GLI1/GLI2 remains an unresolved issue.

**Fig. 8:**
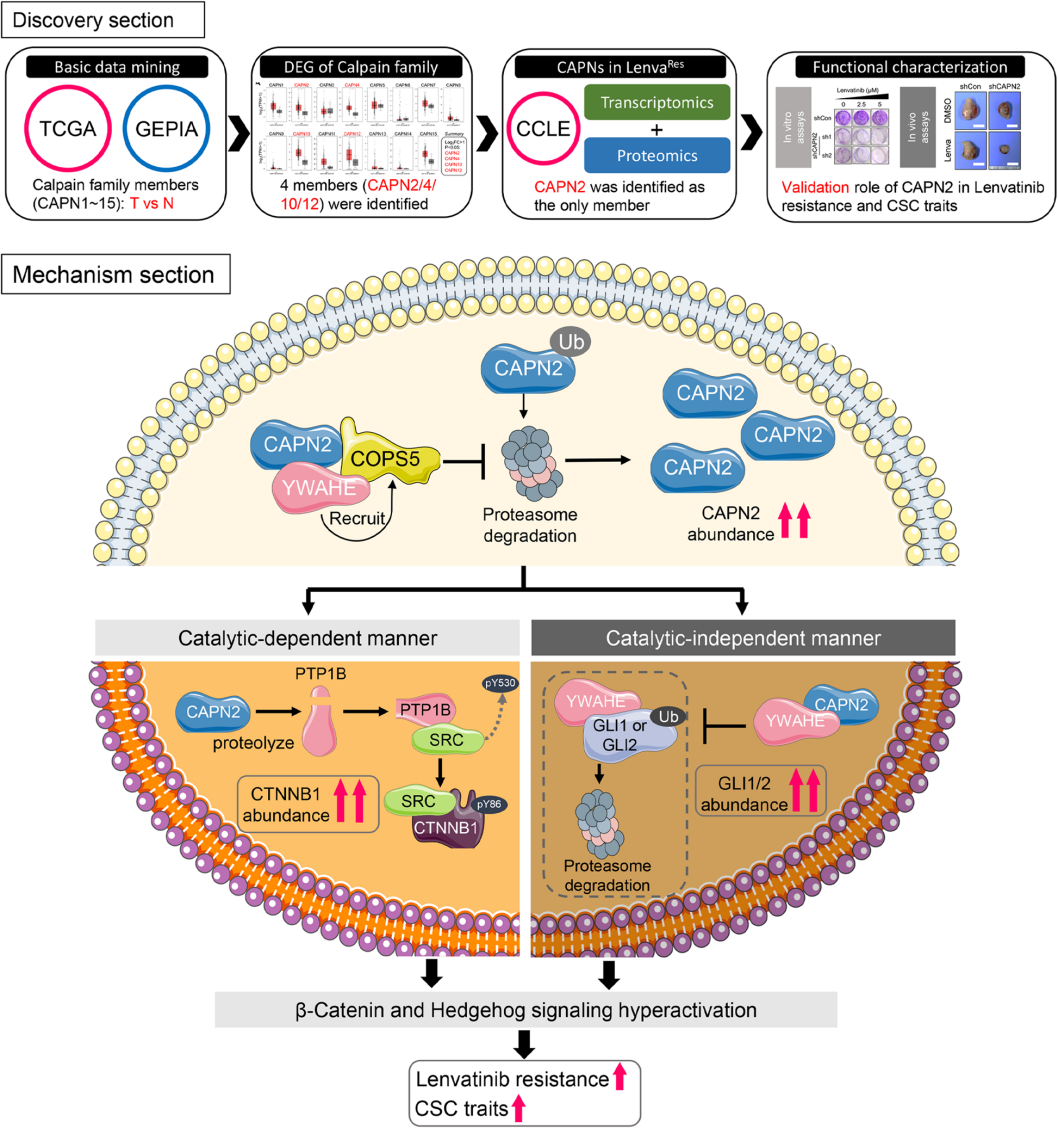
Diagram illustration of present study. CAPN2 exerted its function through promoting β-Catenin and Hedgehog signaling simultaneously: 1) activating β-Catenin signaling through its enzyme activity, and 2) preventing GLI1/GLI2 degradation through direct binding to YWHAE in an enzyme-independent manner, which disrupts the association between YWHAE and GLI1/GLI2 to inhibit YWHAE-induced degradation of GLIs. Moreover, intracellular CAPN2 protein stability was also enhanced by YWHAE binding, as it could serve as a scaffold to recruit deubiquitinase COPS5 to prevent proteasome degradation of CAPN2

In summary, this study identified CAPN2 as a novel contributor for CSC traits and Lenvatinib resistance in HCC. Moreover, we revealed CAPN2 exerted its regulatory function by activating β-catenin signaling in an enzyme-dependent manner, and also by binding to YWHAE to restrain YWHAE-induced GLI1/GLI2 degradation in an enzyme-independent manner, which promoted Hh signaling activation (Fig. 8). Our present study might provide novel insight into the Lenvatinib resistance mechanism.

## Materials and methods

### Patients and follow-up

We collected two cohorts of HCC patients in this study. Cohort 1: A total of 12 advanced HCC patients received Lenvatinib treatment who had undergone surgical resection prior to Lenvatinib therapy were retrospectively enrolled from November 2021 to February 2022, and these patients received Lenvatinib treatment after recurrence or metastasis (10 of 12 suffered intrahepatic recurrence within 1 year after resection; 2 of 12 suffered lung metastasis within 1 year after resection). Frozen liver cancerous tissues and adjacent normal liver tissues were collected in Shanghai Cancer Center. These samples were subjected to immunoblotting assay to determined difference of CAPN2 expression between responsive and non-responsive patients (6 of 12 were identified as resistant). Cohort 2: we randomly collected 6 fresh HCC samples in February 2022 in Shanghai Cancer Center. These samples were subjected to sphere-forming assays for evaluating CSC potential, and WB assays for determining CAPN2 expression states. These patients were stratified by their median CAPN2 expression (Fig. S9), and 3 of 6 were considered as CAPN2-high. Moreover, spheres from 2 of CAPN2-high patients were further used for CAPN2 knockdown experiments. This study was approved by the Shanghai Cancer Center Research Ethics Committee (Approval Number: 050432–4-2108*), and all individuals provided informed consent for inclusion of their tissue. The inclusion and exclusion criteria were as follows: 1) HCC diagnoses were based on histopathological examinations; 2) no prior anti‐cancer therapy before resection and Lenvatinib therapy; 3) complete removal of all tumor nodules, without any loci observed on the incision surface according to histological tests; 4) availability of sample collection; and 5) complete clinicopathologic and follow‐up data. Follow-up was conducted as previously described [41, 42]. Follow-up ended in August 2023 (cohort 1). Disease progression was evaluated according to the modified Response Evaluation Criteria in Solid Tumors (mRECIST) criteria [43]. Patients were divided into 4 subgroups: complete response (CR), partial response (PR), stable disease (SD), or progressive disease (PD). Patients with CR or PR diseases were considered responsive, while patients with SD or PD were considered as non-responsive.

### Protein extraction and immunoblotting assays

Total cell/tissue protein was extracted using RIPA lysis buffer (Beyotime, Nantong, China) supplemented with 0.1 mM PMSF and protease inhibitor cocktail (Beyotime, China) according to the manufacturer’s instructions. Bicinchoninic Acid (BCA) Assay Kit (Beyotime, China) was used for the quantification of extracted protein. Equal quantities of protein lysates were resolved by sodium dodecyl sulfate–polyacrylamide gel electrophoresis (SDS-PAGE), then transfer to polyvinylidene fluoride (PVDF) membranes (0.45 μm, Beyotime, China). Membranes with protein lysates were incubated with specific primary antibodies overnight at 4 °C. After removing unconjugated primary antibodies by TBST washing, membranes were further incubated with the horseradish peroxidase (HRP)-conjugated secondary antibody. After washing three times with TBS supplemented with 0.1% Tween-20, bands on the membrane were visualized by applying BeyoECL moon Kit (Beyotime, China). The primary antibodies used are listed as Supplementary Table 1.

### RNA extraction and qRT-PCR

Total RNA was extracted by RNAeasy mini kit (Qiagen, Germany) according to manufacturer’s instructions. NanoDrop 2000 (ThermoFisher, USA) was used for the quantification of extracted RNA. For cDNA was synthesized, SuperScript IV First-Strand Synthesis System kit (Invitrogen, USA) was used according to manufacturer’s instructions. qRT-PCR was conducted using TB Green Fast qPCR Mix (Takara, China). DNA amplification was carried out using a DX-II device (ABI, USA). The relative quantities of target gene were calculated by the ΔCq method (referred to the expression of internal control gene). PCR were performed as follows: 5 min at 95 °C, followed by 40 cycles of 95 °C for 20 s and 60 °C for 60 s. β-Actin was used as an internal control. Primers are listed as Supplementary Table 2.

### Cell culture and animal models

SNU387, SNU449 and SNU182 cell lines were purchased from BeNa Culture Collection (BNCC) corporation. Hep3B and Huh7 cell lines were purchased from Shanghai Institute of Cell Biology (Chinese Academy of Sciences). JHH7 cell line was preserved in our lab. SNU387, SNU449, SNU182 and JHH7 cell lines were cultured in 1640 (Gibco, Grand Island, NY, USA) supplemented with 10% fetal bovine serum (Gibco, Grand Island, NY, USA) at 37 °C with 5% CO_2_. Hep3B and Huh7 cells were maintained in DMEM (Gibco, Grand Island, NY, USA) supplemented with 10% fetal bovine serum at 37 °C with 5% CO_2_. For in vivo experiments, different concentrations of SNU387-shControl, SNU387-shCAPN2 cells were suspended in a mixture of 100 μL serum-free DMEM and Matrigel (BD Biosciences, Franklin Lakes, NJ, USA) at 1:1 volumetric according to a previous study [44]. Thereafter, these cell mixtures were subcutaneously injected into the upper flank of 4-week-old male BALB/c nude mice (In drug resistance experiment, shControl group: 3/group, shCAPN2 group: 6/group; In serial dilution experiment, 6 mice/group). Tumor dimensions were measured, and tumor volumes (mm3) were calculated according to follows: V = ab^2^ ÷ 2, where a and b are the largest and smallest tumor diameters, respectively. Establishment of animal models and treatment assays were approved by The Research Ethical Committee of Shanghai Cancer Center (Approved Number: FUSCC-IACUC-S2022-0209).

### Statistical analysis

Statistical analyses were performed using SPSS v21.0 (IBM, Armonk, NY, USA) or GraphPad Prism v8 (GraphPad Software, CA, USA). Quantification values are presented as mean ± standard deviation (SD). Continuous data were evaluated to observe whether they were normal distribution by using one-way ANOVA. If data was normal distribution, two-tailed Student's t-test was performed to explore the significance. Chi-square test or Pearson's test were conducted to analyze the correlations between categorical variables. A *P*-value less than 0.05 was defined as statistically significant.

Other detailed information about materials and methods could be seen in Supplementary Methods and Materials.

## Supplementary Information

Supplementary Material 1.

Supplementary Material 2.

## Data Availability

The datasets used and/or analyzed during the current study are available from the corresponding author on reasonable request.
