# Supplementary material for: Calpain 2 promotes Lenvatinib resistance and cancer stem cell traits via both proteolysis-dependent and independent approach in hepatocellular carcinoma
Source: Mol Biomed. 2024 Dec 31;5:74. doi: 10.1186/s43556-024-00242-7 (PMC11688263; doi:10.1186/s43556-024-00242-7)
Supplement: Supplementary file 1 — Supplementary Material 1. [file 43556_2024_242_MOESM1_ESM.docx]

**Calpain 2 promotes Lenvatinib resistance and cancer stem cell traits via both proteolysis-dependent and independent approach in hepatocellular carcinoma**

Ma*, et al.*

**Content**

1. Supplementary Method and Materials

2. Supplementary Table 1. Antibodies used for WB and IP assays.

3. Supplementary Table 2. Primers used for RT-PCR assays.

4. Supplementary Fig. 1: Gene set enrichment analysis revealed stem cell trait molecular signatures were enriched in CAPN2-high HCC according to TCGA dataset.

5. Supplementary Fig. 2: Immunoblotting assays confirmed the knockdown efficiencies of β-Catenin in indicated HCC cells.

6. Supplementary Fig. 3: Effects of specific inhibitors for CAPN2 on Lenvatinib resistance in HCC cells received indicated treatment.

7. Supplementary Fig. 4: Gene set enrichment analysis revealed Hedgehog signaling signatures were enriched in CAPN2-high HCC according to TCGA dataset.

8. Supplementary Fig. 5: Representative images of spheres derived from clinical samples which received indicated treatment.

9. Supplementary Fig. 6: WB assay results for the dynamic changes of CSC-related markers upon indicated treatment in SNU387 and SNU182 cells.

10. Supplementary Fig. 7: GLI1 and GLI2 mRNA expression alterations due to CAPN2 knockdown in HCC cells were determined by RT-PCR assays.

11. Supplementary Fig. 8: CAPN2 mRNA expression alterations due to YWHAE knockdown in HCC cells were determined by RT-PCR assays.

12. Supplementary Fig. 9: WB assay results for CAPN2 expression in clinical HCC samples.

**1. Supplementary Methods and Materials**

**Bioinformatic analysis**

Gene Expression Profiling Interactive Analysis (GEPIA, http://gepia.cancer-pku.cn/), which sources data from The Cancer Genome Atlas (TCGA, https://tcga-data.nci.nih.gov/tcga/) were used to evaluate differential expression patterns of calpain family members between HCC and normal liver tissues. Cancer Cell Line Encyclopedia (CCLE) dataset was used to compare the differential expression of calpain family members between Lenvatinib-resistant and Lenvatinib-sensitive HCC cell lines. Specifically, we inquired CAPN2 protein expression data from CCLE dataset to validate the elevated protein levels of CAPN2 in Lenvatinib-resistant HCC cells. Protein Interaction Network Analysis (PINA, version 3.0)^[1]^ was applied for searching the interactors of CAPN2. Expression profiling data were extracted from TCGA Liver hepatocellular carcinoma (LIHC) dataset (https://portal.gdc.cancer.gov). HCC patients were stratified by their CAPN2 expression status according to quartile range (low: 0%–25%; high: 75%–100%) and differentially expressed genes (DEGs) were identified by comparing expression profiling results between the CAPN2-high and CAPN2-low subgroups using DESeq2 [1.36.0] and edgeR [3.38.2]. Gene set enrichment analysis (GSEA) was used for clustering DEGs according to clusterProfiler [4.4.4]. In addition, the gene set used for GSEA was based on the MSigDB database (https://www.gsea-msigdb.org/gsea/msigdb/collections.jsp). Moreover, BioGRID (https://thebiogrid.org) datasets were also investigated for identifying potential regulator for CAPN2 protein stability.

**Regents and treatments**

To specific inhibit β-Catenin signaling activation, 25μM of ICG-001 (Selleck, China) was applied for in vitro experiments. Additionally, for hindering the activities of GLI1 and GLI2, 10μM of GANT61 (Selleck, China) was applied for in vitro experiments. To restrain intracellular protein synthesis process, cycloheximide (CHX) was applied at a concentration of 50μg/ml, and HCC cells treated with CHX were collected at indicated time point for subsequent protein extraction, followed by immunoblotting assays. For inhibiting CAPN2 catalytic activity, 100 μmol/L of selective CAPN2 antagonist, calpain inhibitor IV, was applied. Finally, to prevent endogenous protein degradation, HCC cells were pretreated with 20μM MG132 for 5-6 hours prior to collection and further experiments.

**Protein extraction and immunoblotting assays**

Total cell/tissue protein was extracted was conducted using RIPA lysis buffer (Beyotime, Nantong, China) supplemented with 0.1 mM PMSF and protease inhibitor cocktail (Beyotime, China) according to the manufacturer’s instructions. Extracted protein concentrations were determined by Bicinchoninic Acid (BCA) Assay Kit (Beyotime, China). Equal quantities of protein lysates were resolved by sodium dodecyl sulfate-polyacrylamide gel electrophoresis (SDS-PAGE), followed by transfer to polyvinylidene fluoride (PVDF) membranes (0.45 μm, Beyotime, China). Membranes were then incubated with specific primary antibodies overnight at 4°C. After removing unconjugated primary antibodies, membranes were further incubated with the appropriate horseradish peroxidase (HRP)-conjugated secondary antibody. After washing three times with Tris-buffered saline supplemented with 0.1% Tween-20, immunoreactive bands on the membrane were visualized with BeyoECL moon Kit (Beyotime, China), which allowed detection via an enhanced chemiluminescence approach. The primary antibodies used are listed as **Supplementary Table 1**.

**RNA extraction and qRT-PCR**

Total RNA was extracted by RNAeasy mini kit (Qiagen, Germany) according to manufacturer’s instructions. Extracted RNA was quantified using NanoDrop 2000 (ThermoFisher, USA), and cDNA was synthesized via using SuperScript IV First-Strand Synthesis System kit (Invitrogen, USA) according to manufacturer’s instructions. qRT-PCR was conducted with TB Green Fast qPCR Mix (Takara, China). DNA amplification was carried out using a DX-II (ABI, USA). The relative quantities of target gene mRNAs compared to an internal control were calculated by the ΔCq method. PCR conditions were as follows: 5 min at 95 °C, followed by 40 cycles of 95 °C for 15 s and 60 °C for 60 s. β-Actin was used as an internal control. Primers are listed as **Supplementary Table 2.**

**Plasmids, transduction, and small interfering RNA (siRNA) transfection**

Short hairpin RNAs (shRNAs) targeting specific region of CAPN2 genes were cloned into pLKO.1 vector, or entire CDS sequence of target (full-length CAPN2, regulatory domain CAPN2, catalytic domain CAPN2) were cloned into pLenti-CMV-puro plasmid. Scramble shRNA and empty pLenti-CMV-puro vector were applied as controls, respectively. Lentivirus was produced in HEK293 cells transfected with above pLKO.1-shRNA or pLenti-CMV-puro plasmids as previous described^[2]^. 72 hours after transfection, virus supernatant was collected, and viral particles were precipitated by PEG-8000 as previous reported^[3]^. To generate stable transfected cancer cells, lentivirus stocks were applied to transduce SNU387, SNU182 cells, and JHH7 cells with 8mg/L polybrene. Then, cells were culture in selective medium containing 2.5mg/L puromycin 48 hours after virus transduction for 7-12 days. Target sequences for specific genes were described as follow: shCAPN2-1: 5’- CCCGAGAATACTGGAACAATA-3’; shCAPN2-2: 5’- CAGGAACTACCCGAACACATT-3’; The siRNAs targeting β-Catenin were purchased from Merdobio, and transfected into indicated HCC cells via using Lipofectamine 3000 reagent (Gibco, USA) according to the manufacturer’s instructions. Target sequences were listed as below: si-β-Catenin -1: Guide, 5‘-UCAUUAUAUUUACUAAAGCUU-3‘, Passenger, 5’-GCUUUAGUAAAUAUAAUGAGG-3’; si-β-Catenin-2: Guide, 5‘-AAACAUUAAAGUUAAUAACUU-3’, Passenger, 5’-GUUAUUAACUUUAAUGUUUUU-3’; si-YWHAE-1: Guide, 5’-UGAAAAAGCCUCUAUGUAGUC-3’, Passenger, 5’-CUACAUAGAGGCUUUUUCAGC-3’; si-YWHAE-2: Guide, 5’-AGUAAAGUAGGCAAGAAUGAG-3’, Passenger, 5’-CAUUCUUGCCUACUUUACUCU-3’. Scrambled siRNA was purchased from Santa Cruz (Control siRNA-A).

**Cell counting kit-8 (CCK-8) and colony formation assays**

CCK8 and colony formation assays were performed according to our previous study. Briefly, for CCK-8 assays, the indicated HCC cells were seeded at a density of 1,000~3000 cells per well depending on proliferation state of indicated cells in a 96-well plate. Seeded cells were cultured for overnight to allow the cell adhering to the well (defined as Day 0). Afterwards, 10 μL CCK-8 working solution (Dojindo, Japan) was added into each well at the indicated time points. Then, cells were incubated at 37°C for 2 hours. Finally, the optical density of each well was measured at 450 nm using a microplate reader (Thermo Fisher) to detemine the cell viability. For colony formation assays, HCC cells were seeded at a density of 5×10^3^–1×10^4^ cells per well, depending on growth rate cells, and cultured in complete DMEM containing 10% FBS and 1% antibiotics for 10-14 days, depending on the growth states of control group. Surviving cell colonies were fixed with methanol and then stained with 0.1% crystal violet for further quantification of colonies.

**Sphere formation assay**

Sphere formation assay was conducted according to previous research^[4]^. Briefly, cells derived from HCC cell lines were cultured at a density of 2000 cells/ml; whereas, cells derived from fresh HCC tissues were cultured at a density of 20000 cells per well. Serum-free medium for sphere forming culture was composed of DMEM/F12 medium supplemented with 100 IU/ml penicillin, 100 μg/ml streptomycin, 40 ng/ml human recombinant epidermal growth factor, 50 ng/ml human recombinant basic fibroblast growth factor, 10 ng/ml human hepatocyte growth factor, 1% nonessential amino acids, 1% GlutaMax, and 2% B27 supplement (Invitrogen, USA). Sphere forming culture lasted for 14 days for HCC cell lines and 21 days for primary HCC cells.

**Limiting dilution xenograft assay**

In present study, tumorigenicity was conducted in six-to-eight-week-old male nude mice, following serial dilution, to form tumor nodules within a certain time interval. Nude mice were randomly divided into groups (six mice per group) received indicated number of HCC cells, and maintained under standard conditions, according to institutional guidelines. HCC cells were harvested and re-suspended in a serum-free DMEM/Matrigel (BD Biosciences, USA) mixture (1:1 by volume), and injected subcutaneously into the flanks of recipient nude mice. Tumor formation was monitored every two weeks following injection, and tumor size as well as incidence of tumor formation were recorded. The experiment was terminated six weeks after initial tumor cell injection, at which point mice with no apparent tumor nodules at the injection site were considered negative.

**Primary HCC cell separation**

Primary HCC cells from fresh tumor tissues were separated according to previous report^[4]^. In brief, surgical specimens were obtained and these samples were sent to the laboratory within one hour. Immediately, mechanically disaggregated and digested with type IV collagenase (Gibco, USA) and DNAase (Gibco, USA), and re-suspended in DMEM medium. Single-cell suspensions were obtained by filtration through 70 μm and 40 μm filter. Red blood cells were lysed by using ACK buffer (Invitrogen, USA). The number of viable cells was counted and analyzed using Trypan blue before cell sending.

**Co-immunoprecipitation experiment**

Co-IP assays were performed to determine interactions between proteins according to previous reports^[5]^. Briefly, indicated cells were harvested and lysed in NP-40 lysis buffer containing 50 mM Tris–HCl (pH 7.4), 150 mM NaCl and 1% NP‐40, which was supplemented with protease inhibitor cocktail reagent (Beyotime, USA). Immunoprecipitation of protein from cell lysate was performed via using specific antibodies. Antibody-target complex was subsequently immobilized with Sepharose G beads (GE Life Science) and eluted under reducing–denaturing condition in SDS lysis buffer and denaturing at 95°C for 5 min. Afterwards, proteins were detected by WB assays with specific antibodies.

**CHX chasing assay**

CHX chasing assays was performed to determine the half life span of indicated protein under different treatment according to previous study^[5]^. Briefly, indicated HCC cells were treated with CHX (50 μg/ml) for different periods of time. Afterwards, total protein of treated HCC cells was extracted and levels of these proteins were determined by WB analysis.

**Ubiquitination assay**

HCC cells transfected with the indicated plasmids or received indicated regimen were treated with MG132 (20 μM) for 5-6 hours prior to harvest. Afterwards, total protein of treated HCC cell was extracted in 1% SDS and 10 mM N-ethylmaleimide (Sigma), followed by denaturation at 95°C for 10 min. Thereafter, denatured protein extracts were sonicated and diluted 10 times in NP-40 lysis buffer followed by immunoprecipitation with specific antibodies. Finally, immunoblotting assay was performed with anti-Ub to determine the ubiquitination level of specific protein.

**Reference**

[1] Du Y, Cai M, Xing X, Ji J, Yang E, Wu J. PINA 3.0: mining cancer interactome Nucleic Acids Res. 2021 Jan 8;49(D1):D1351-D1357.

[2] Dong ZR, Sun D, Yang YF, et al. TMPRSS4 Drives Angiogenesis in Hepatocellular Carcinoma by Promoting HB-EGF Expression and Proteolytic Cleavage. Hepatology. 2020 Sep;72(3):923-939.

[3] Liao C, Zhang Y, Fan C, et al. Identification of BBOX1 as a Therapeutic Target in Triple-Negative Breast Cancer. Cancer Discov. 2020 Nov;10(11):1706-1721.

[4] Ma XL, Sun YF, Wang BL, et al. Sphere-forming culture enriches liver cancer stem cells and reveals Stearoyl-CoA desaturase 1 as a potential therapeutic target. BMC Cancer. 2019 Aug 1;19(1):760.

[5] Ma XL, Nie YY, Xie SH, et al. ASAP2 interrupts c-MET-CIN85 interaction to sustain HGF/c-MET-induced malignant potentials in hepatocellular carcinoma. Exp Hematol Oncol. 2023 Apr 15;12(1):38.

| **2. Supplementary Table 1. Antibodies used for WB and IP assays** | | |
| --- | --- | --- |
| Target | Brand | Use (dilution) |
| CAPN2 | CST | WB (1:1000) |
|  |  | IHC (1:100) |
| CD44 | ProteinTech | WB (1:1000) |
| CD47 | ProteinTech | WB (1:1000) |
| ICAM1 | ProteinTech | WB (1:1000) |
| OCT4 | ProteinTech | WB (1:1000) |
| SOX2 | ProteinTech | WB (1:1000) |
| SOX9 | ProteinTech | WB (1:1000) |
| Albumin | ProteinTech | WB (1:1000) |
| CK8 | ProteinTech | WB (1:1000) |
| Vinculin | ProteinTech | WB (1:1000) |
| β-Tubulin | ProteinTech | WB (1:500) |
| β-Catenin | ProteinTech | WB (1:1000) |
| GLI1 | ProteinTech | WB (1:1000) |
|  |  | IP (1:100) |
| GLI2 | ProteinTech | WB (1:1000) |
|  |  | IP (1:100) |
| Ubiquitin | CST | WB (1:1000) |
| HA | CST | WB (1:1000) |
|  |  | IP (1:50) |
| Flag | CST | WB (1:1000) |
|  |  | IP (1:50) |
| YWHAE | ProteinTech | WB (1:1000) |
| COPS5 | CST | WB (1:1000) |
|  |  | IP (1:50) |

| **3. Supplementary Table 2. Primers used for RT-PCR assays** | | |
| --- | --- | --- |
|  | Forward (5’->3’) | Backward (5’->3’) |
| CAPN2 | CCAAGCTGGCGAAGGACC | GCAGATCTCCGTGGGGC |
| β-Actin | CATGTACGTTGCTATCCAGGC | CTCCTTAATGTCACGCACGAT |

**4. Supplementary Fig. 1**

**
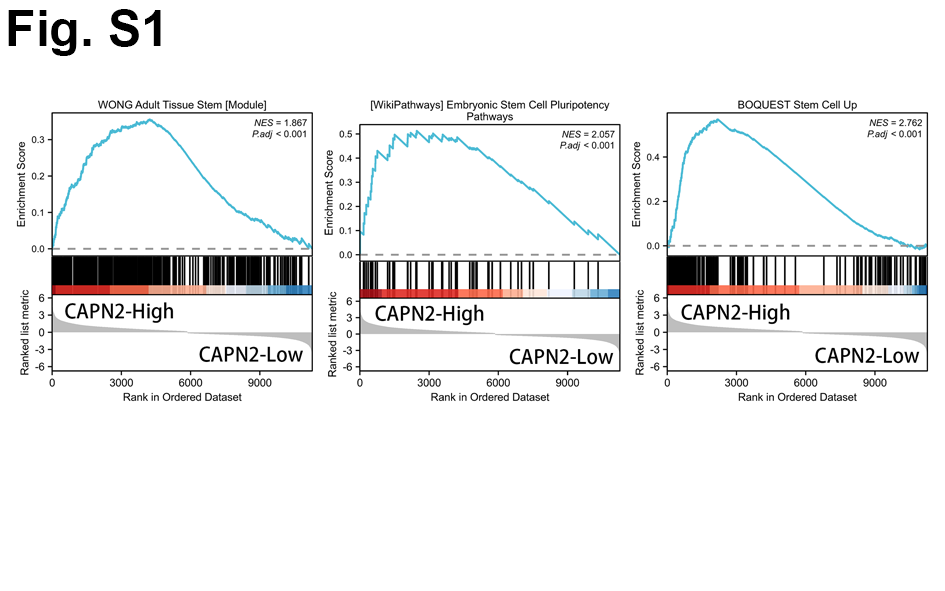
**

**Supplementary Fig. 1:** Gene set enrichment analysis revealed stem cell trait molecular signatures were enriched in CAPN2-high HCC according to TCGA dataset.

**5. Supplementary Fig. 2**


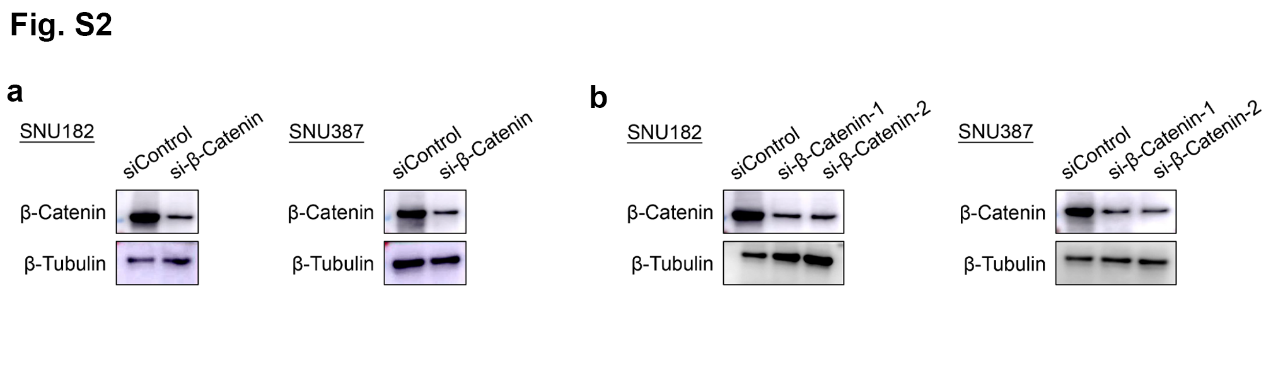


**Supplementary Fig. 2**: Immunoblotting assays confirmed the knockdown efficiencies of β-Catenin in indicated HCC cells.

**6. Supplementary Fig. 3**

**
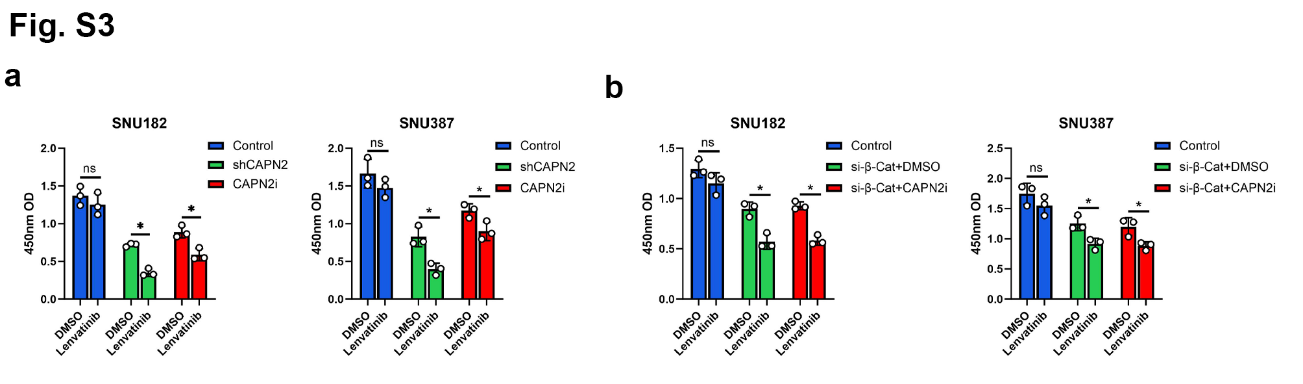
**

**Supplementary Fig. 3**: Effects of specific inhibitors for CAPN2 on Lenvatinib resistance in HCC cells received indicated treatment. (A) CCK8 assay results for CAPN2 inhibitor used alone in SNU182 and SNU387 cells; OD value was determined at Day 4. (B) Results for CAPN2 inhibitor combined with β-Catenin silence in SNU182 and SNU387 cells; OD value was determined at Day 4.

**7. Supplementary Fig. 4**

**
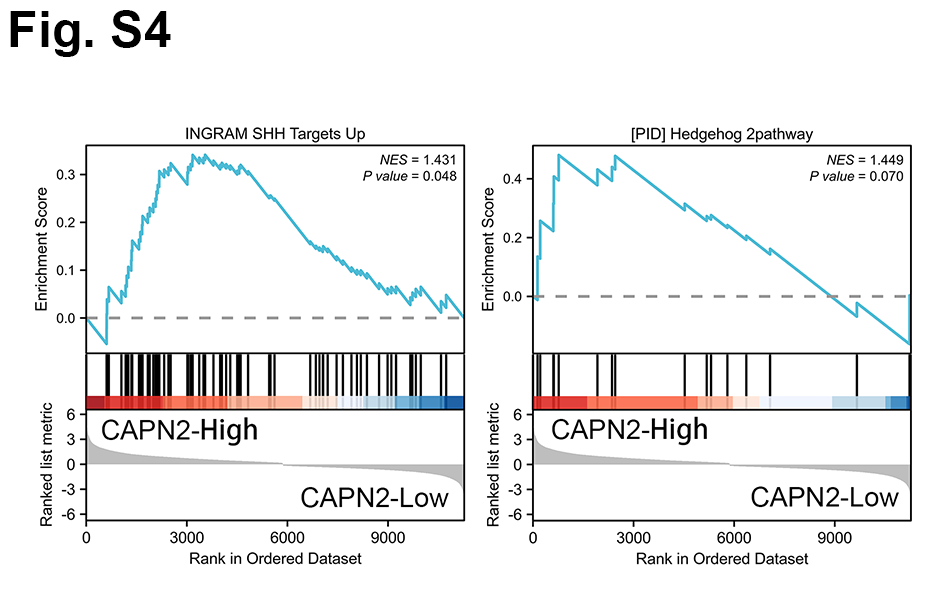
**

**Supplementary Fig. 4**: Gene set enrichment analysis revealed Hedgehog signaling signatures were enriched in CAPN2-high HCC according to TCGA dataset.

**8. Supplementary Fig. 5**

**
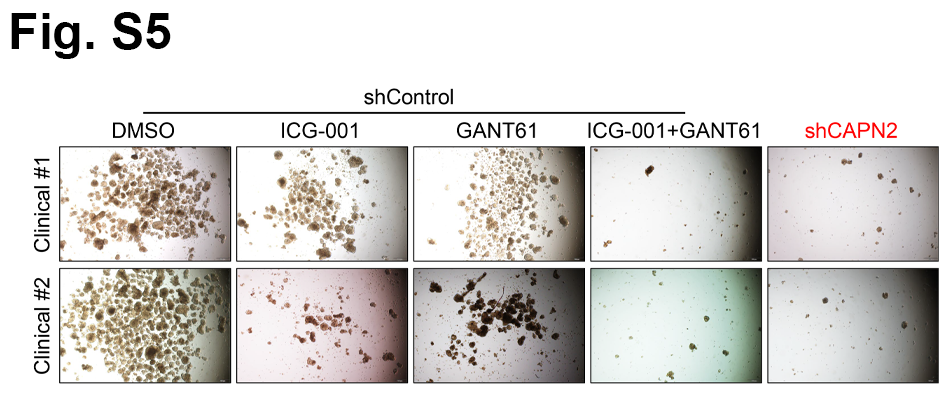
**

**Supplementary Fig. 5**: Representative images of spheres derived from clinical samples which received indicated treatment.

**9. Supplementary Fig. 6**


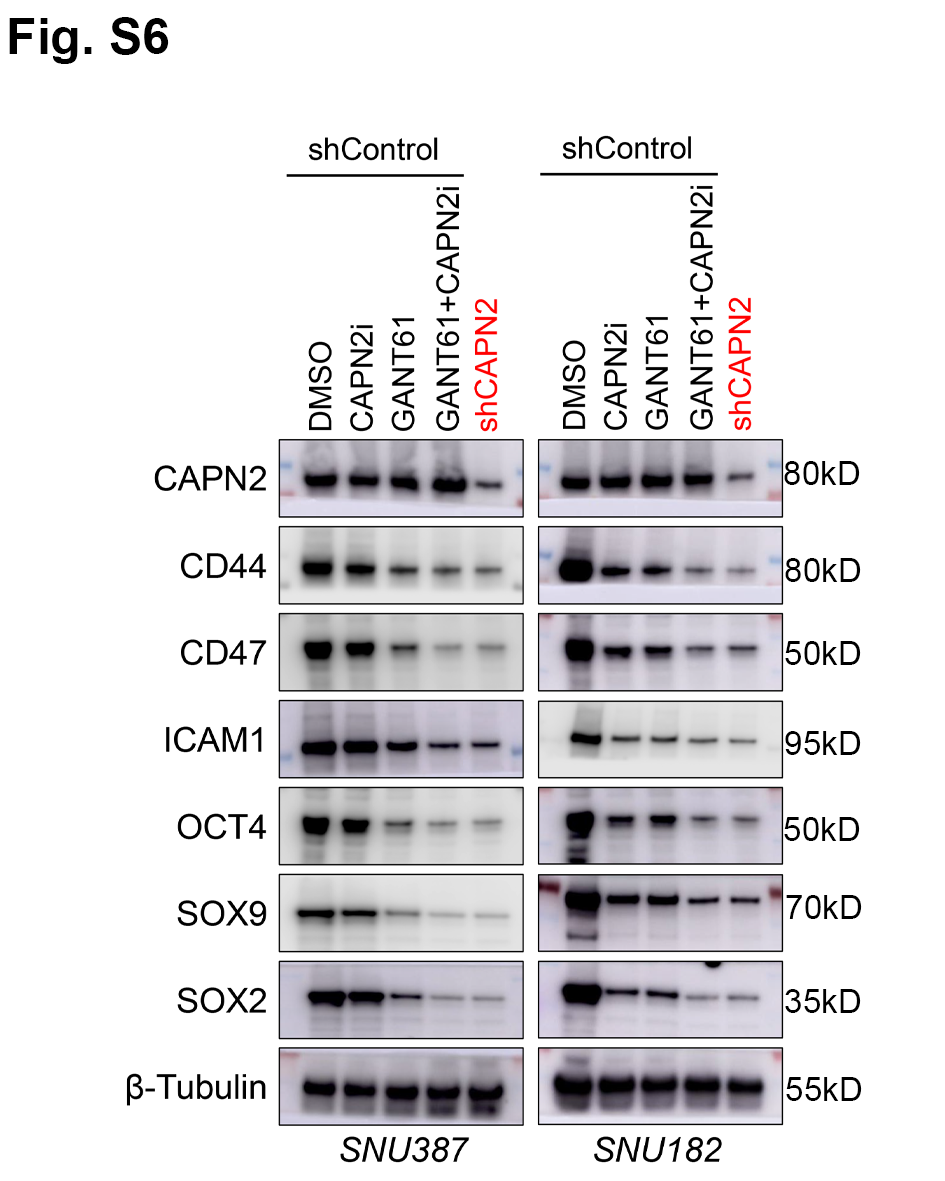


**Supplementary Fig. 6**: WB assay results for the dynamic changes of CSC-related markers upon receiving indicated treatment in SNU387 and SNU182 cells.

**10. Supplementary Fig. 7**

**
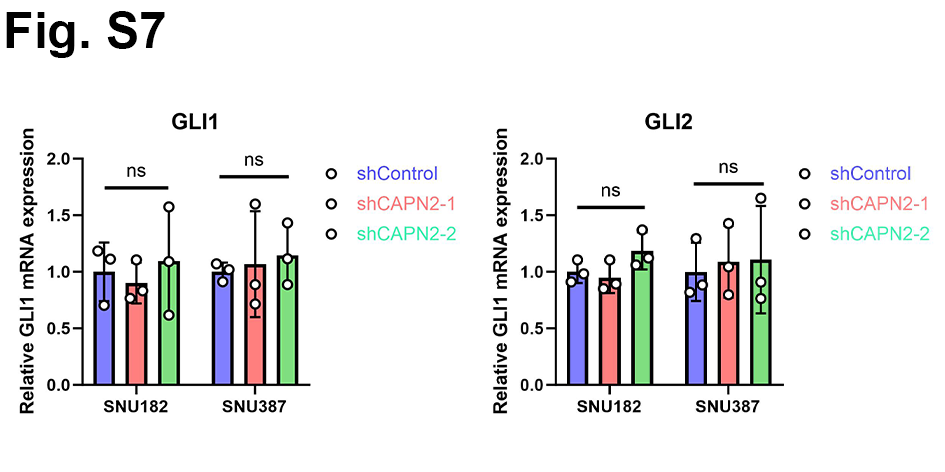
**

**Supplementary Fig. 7**: GLI1 and GLI2 mRNA expression alterations due to CAPN2 knockdown in HCC cells were determined by RT-PCR assays.

**11. Supplementary Fig. 8**

**
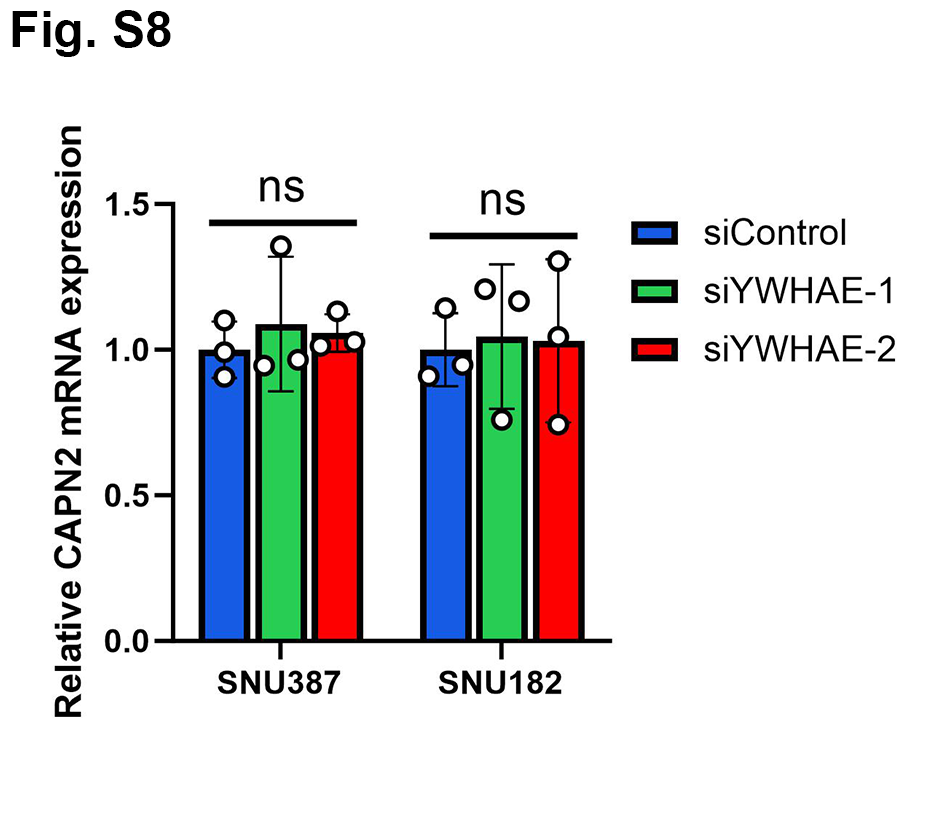
**

**Supplementary Fig. 8**: CAPN2 mRNA expression alterations due to YWHAE knockdown in HCC cells were determined by RT-PCR assays.

**12. Supplementary Fig. 9**

**
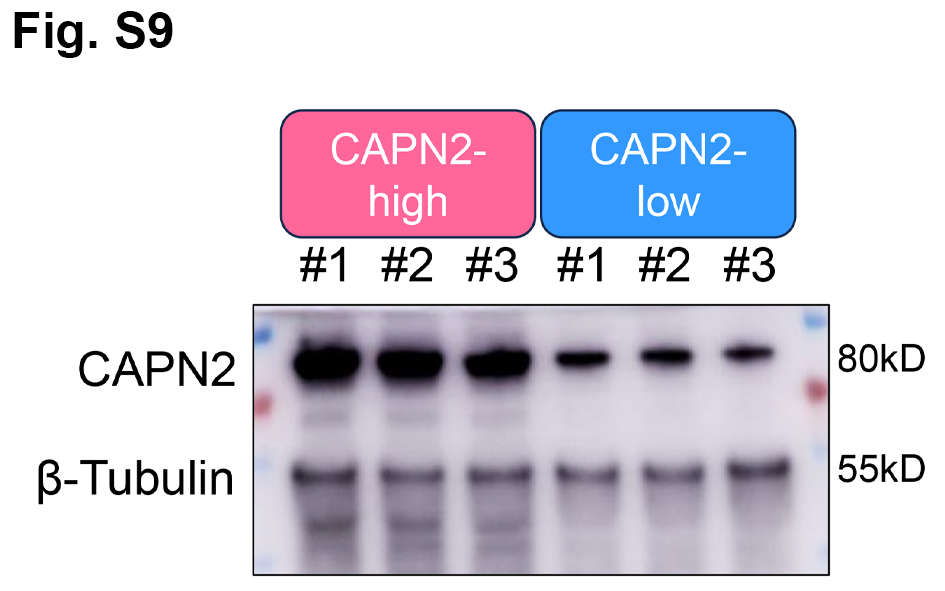
**

**Supplementary Fig. 9**: WB assay results for CAPN2 expression in clinical HCC samples.
