# Supplementary material for: Calpain 2 promotes Lenvatinib resistance and cancer stem cell traits via both proteolysis-dependent and independent approach in hepatocellular carcinoma
Source: Mol Biomed. 2024 Dec 31;5:74. doi: 10.1186/s43556-024-00242-7 (PMC11688263; doi:10.1186/s43556-024-00242-7)
Supplement: Supplementary file 2 — Supplementary Material 2. [file 43556_2024_242_MOESM2_ESM.pdf]

Fig. 1e

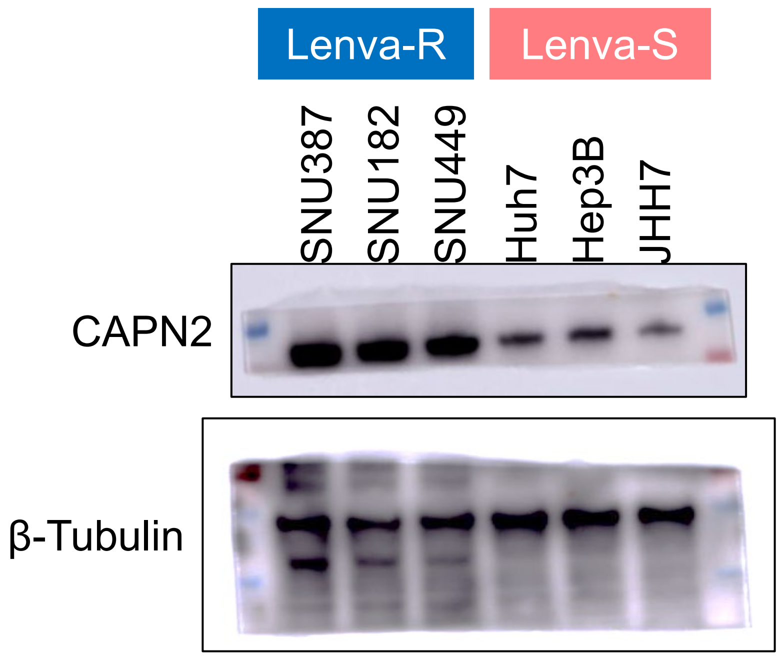

Fig. 1f

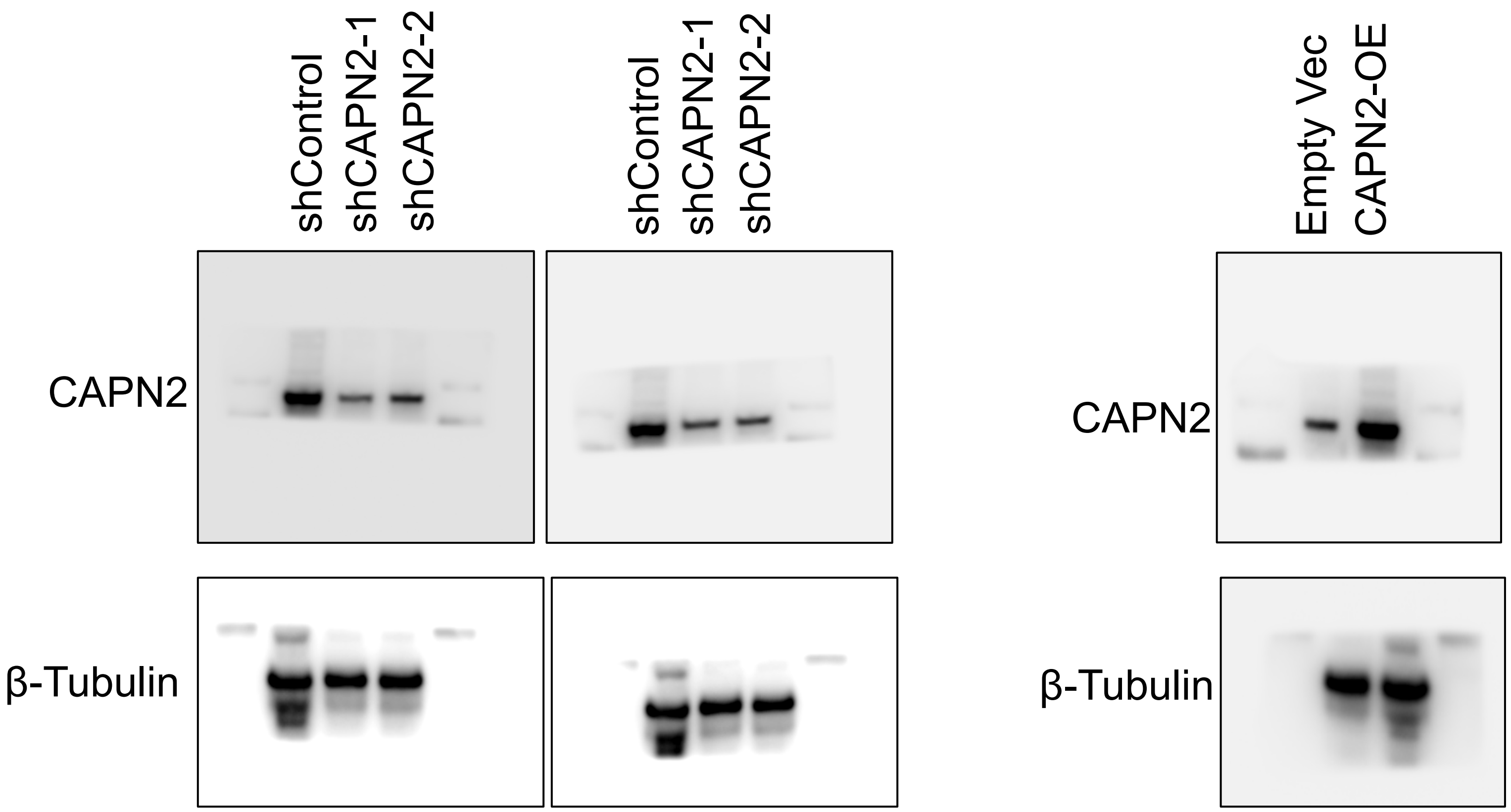

Fig. 7b

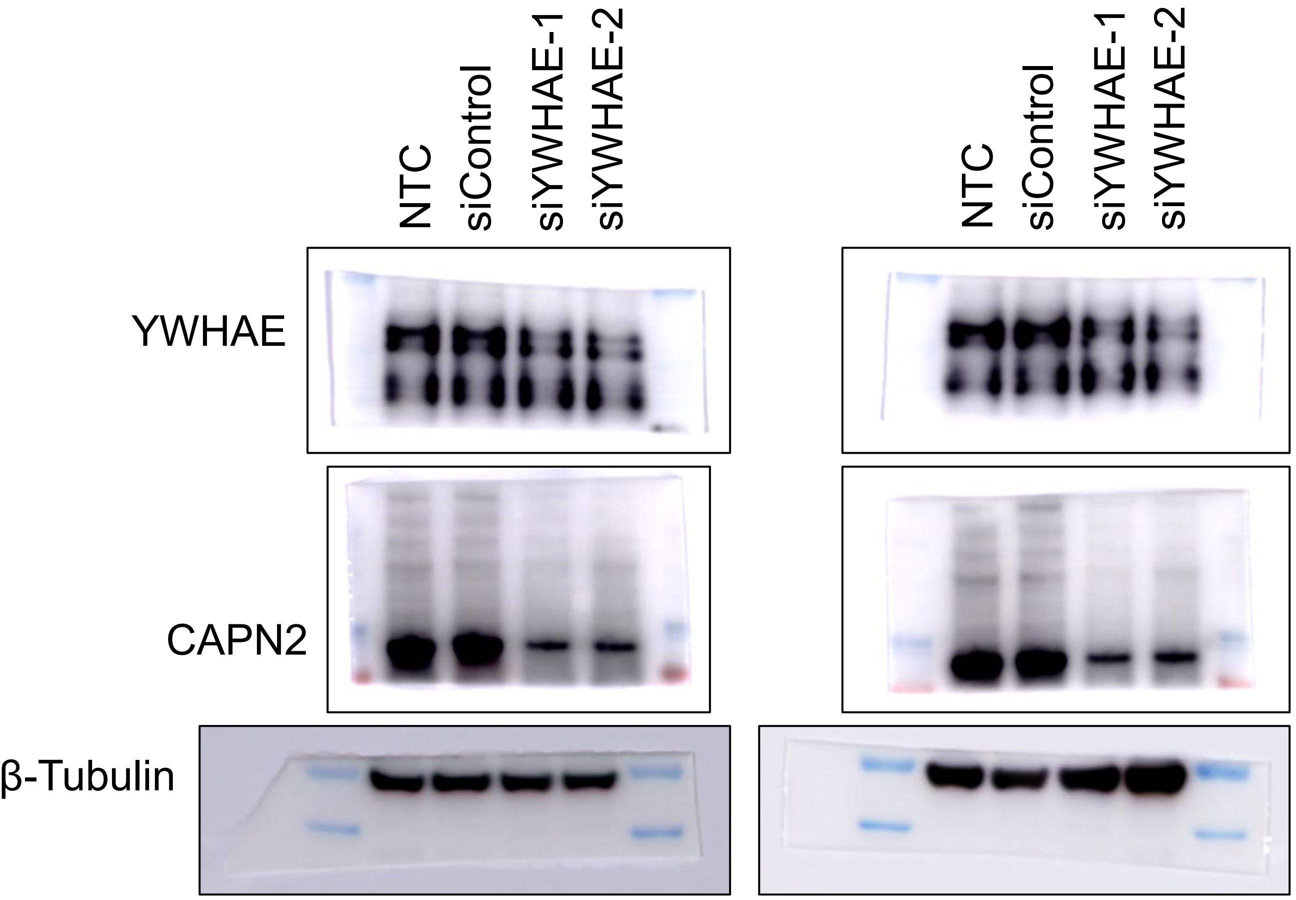

Fig. 1k

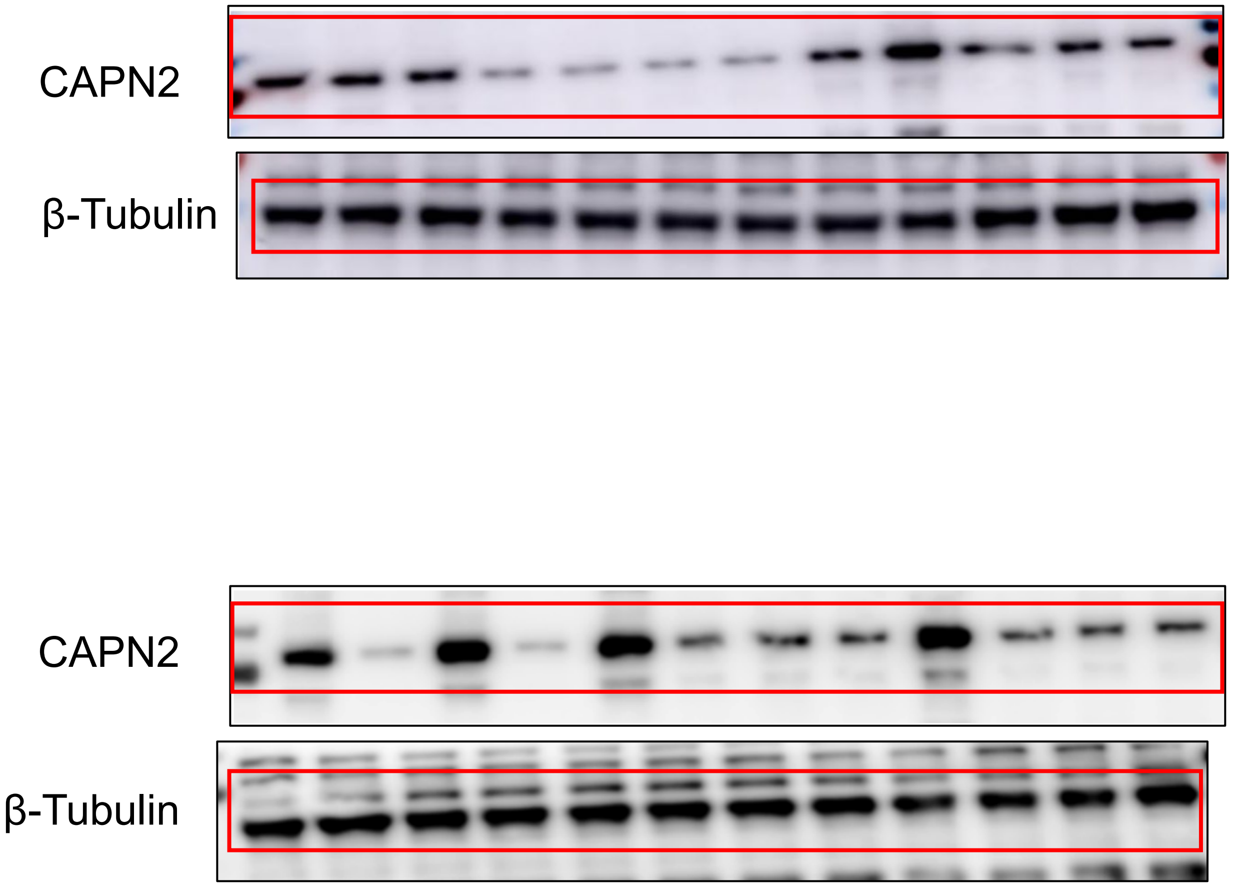

Fig. 2h

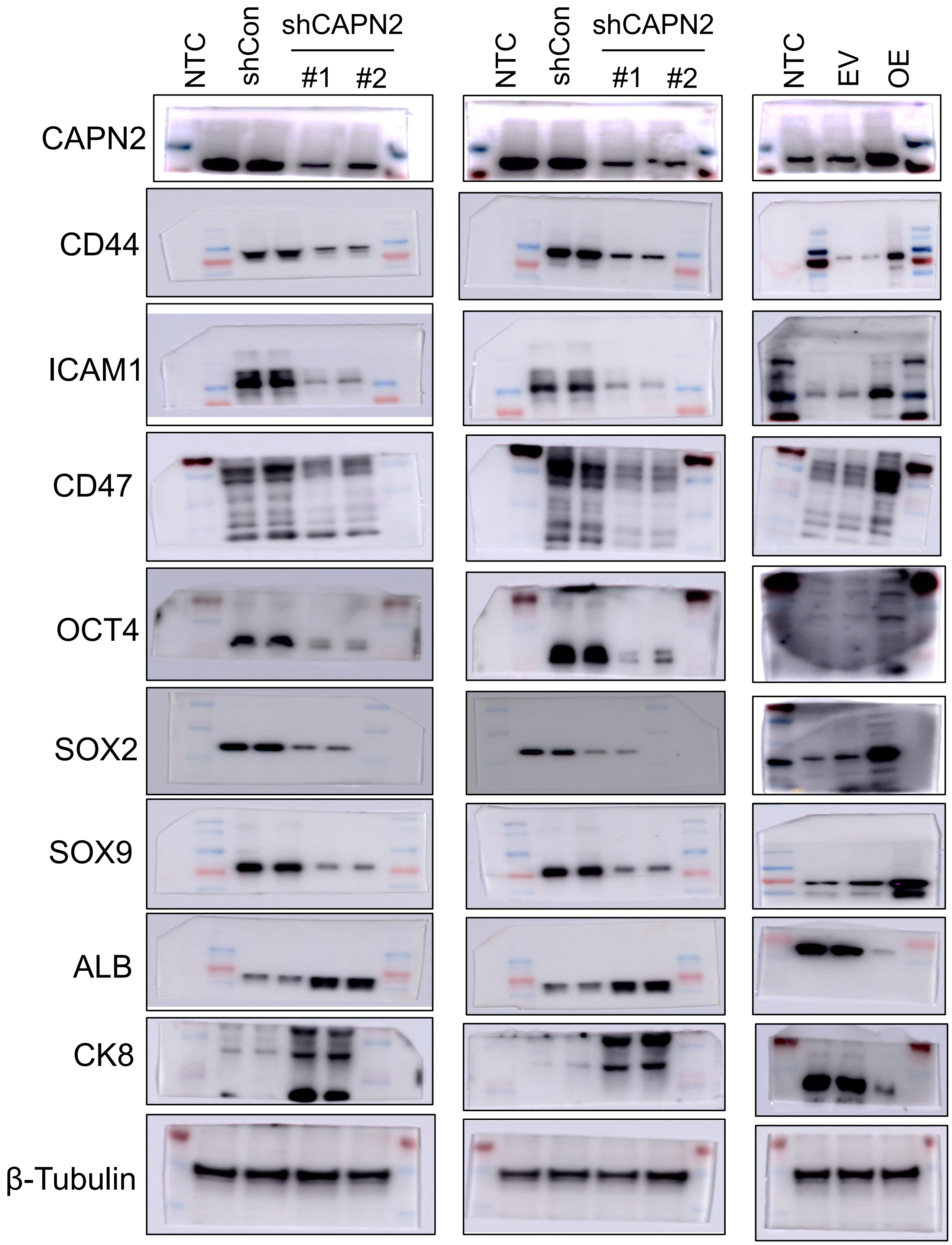
NTC
shCon
shCAPN2
#1
#2
NTC
shCon
shCAPN2
#1
#2
NTC
EV
OE
CAPN2
CD44
ICAM1
CD47
OCT4
SOX2
SOX9
ALB
CK8
β-Tubulin

Fig. 3g

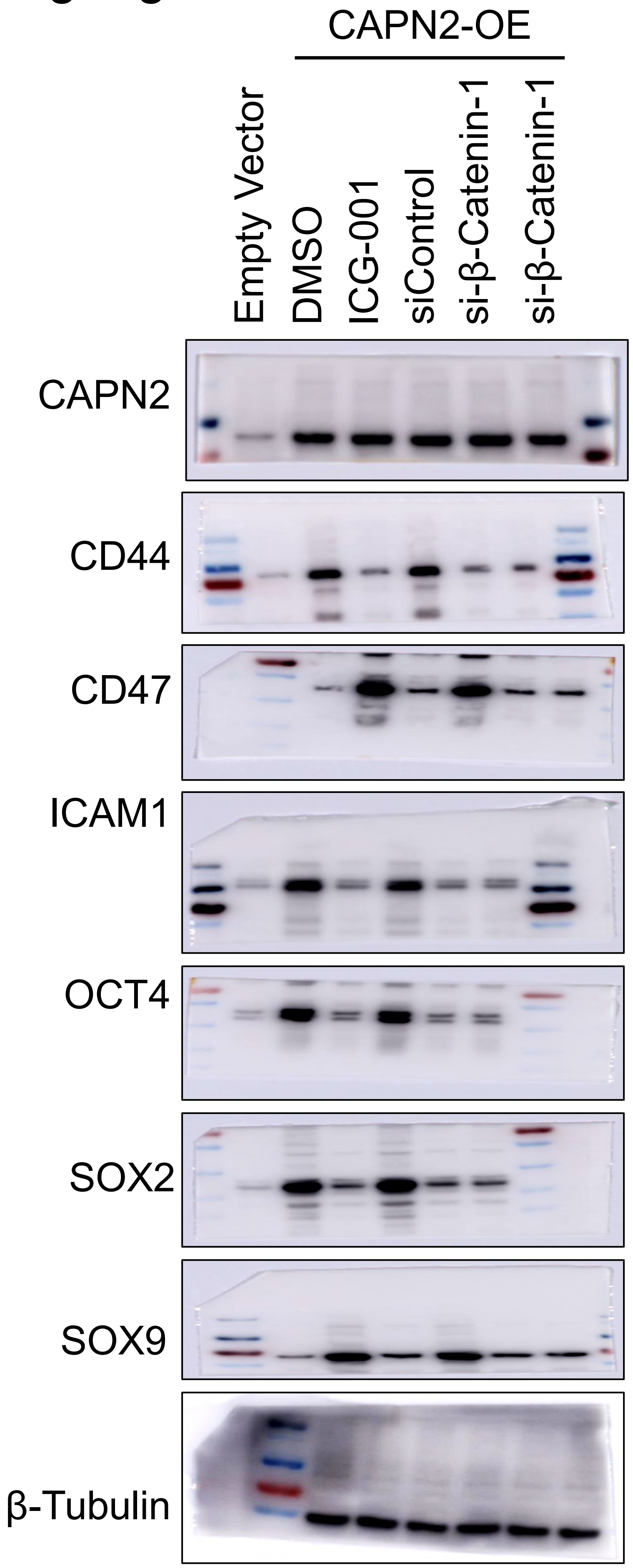
CAPN2-OE
Empty Vector
DMSO
ICG-001
siControl
si-β-Catenin-1
si-β-Catenin-1
CAPN2
CD44
CD47
ICAM1
OCT4
SOX2
SOX9
β-Tubulin

Fig. 2i

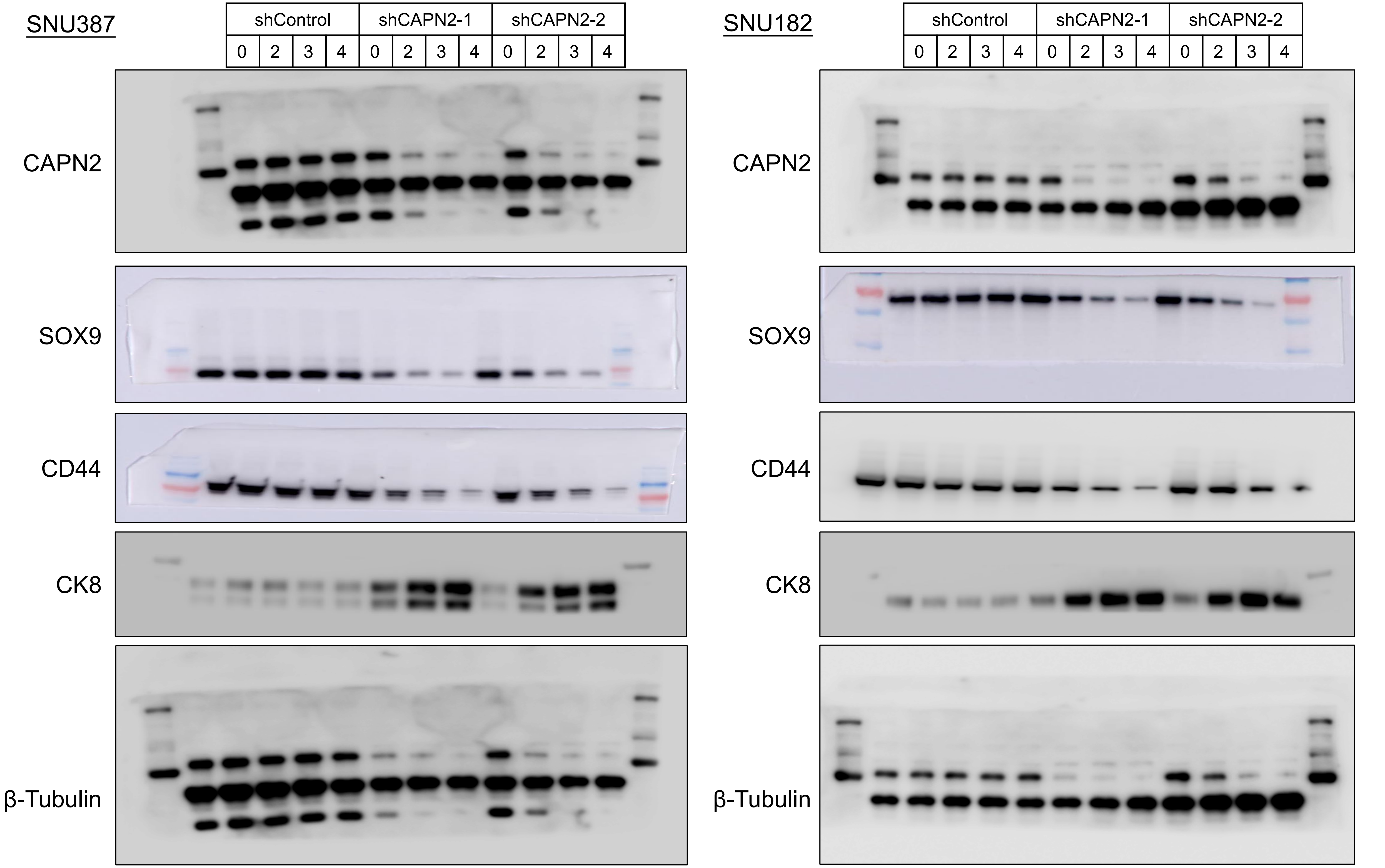

SNU387
shControl
shCAPN2-1
shCAPN2-2
0 2 3 4 0 2 3 4 0 2 3 4
CAPN2
SOX9
CD44
CK8
β-Tubulin
SNU182
shControl
shCAPN2-1
shCAPN2-2
0 2 3 4 0 2 3 4 0 2 3 4
CAPN2
SOX9
CD44
CK8
β-Tubulin

Fig. 2j

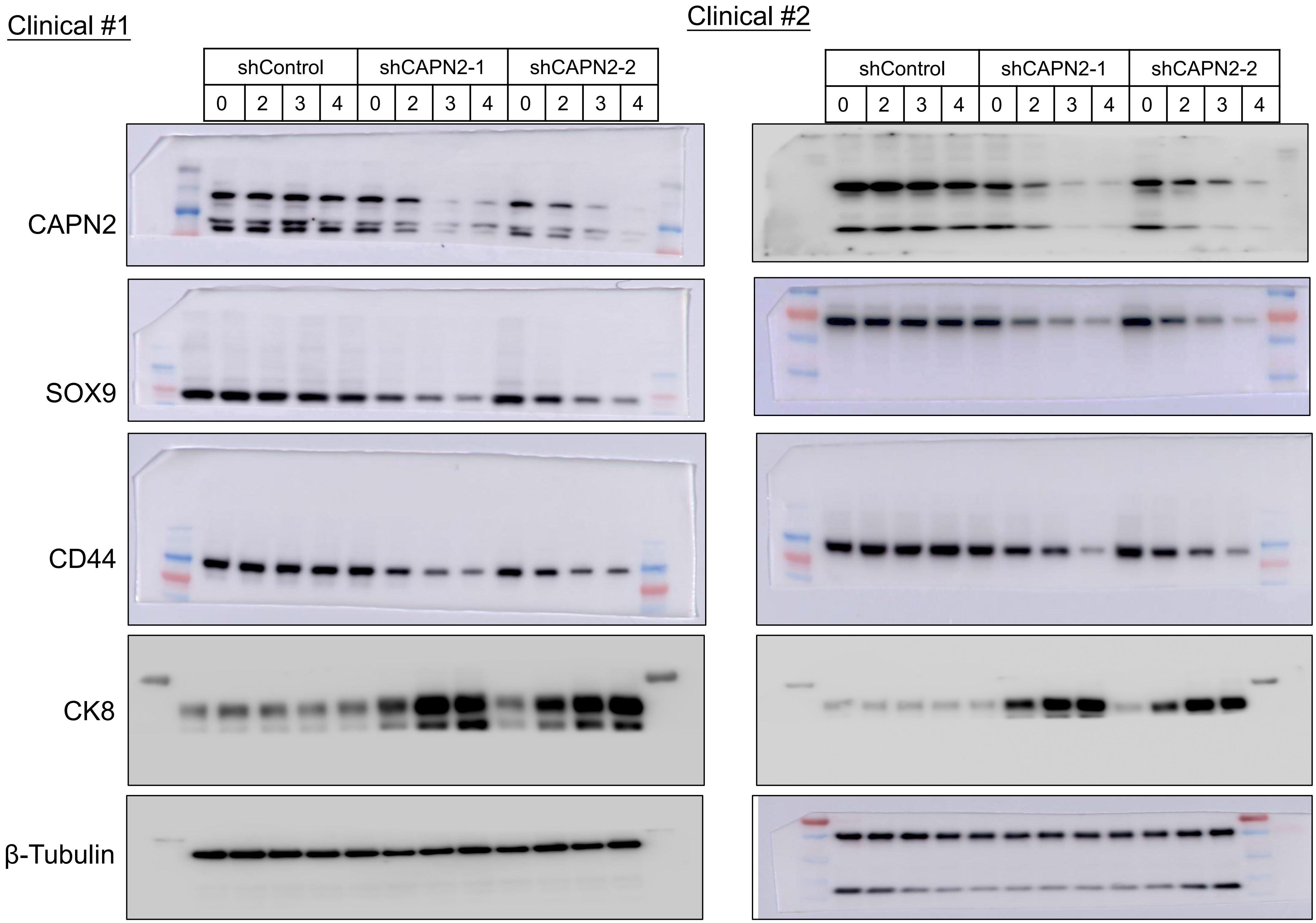
Clinical #1
Clinical #2
shControl
shCAPN2-1
shCAPN2-2
0
2
3
4
CAPN2
SOX9
CD44
CK8
β-Tubulin

Fig. 3f

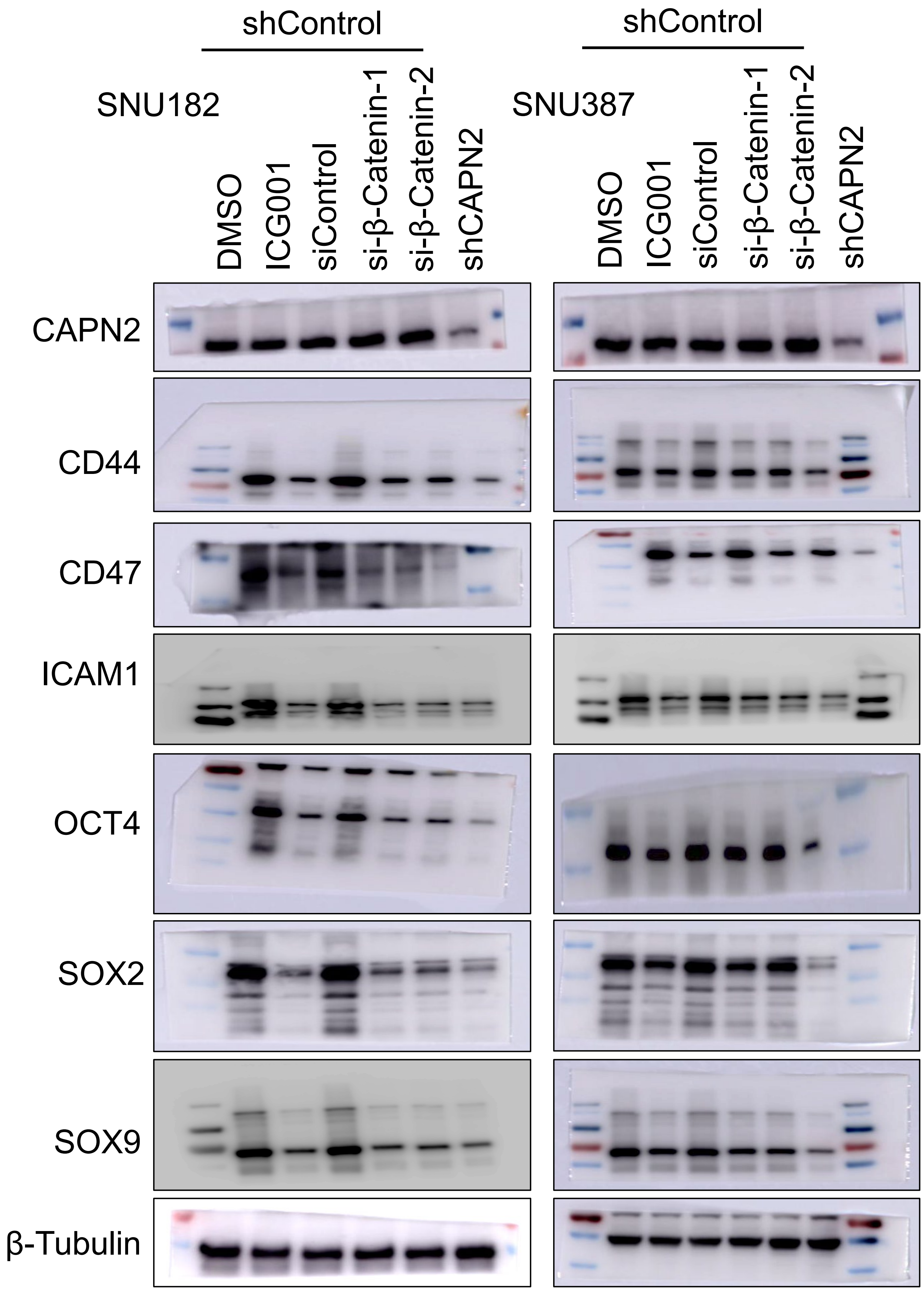
shControl
SNU182
DMSO
ICG001
siControl
si-β-Catenin-1
si-β-Catenin-2
shCAPN2
shControl
SNU387
DMSO
ICG001
siControl
si-β-Catenin-1
si-β-Catenin-2
shCAPN2
CAPN2
CD44
CD47
ICAM1
OCT4
SOX2
SOX9
β-Tubulin

Fig. 3j

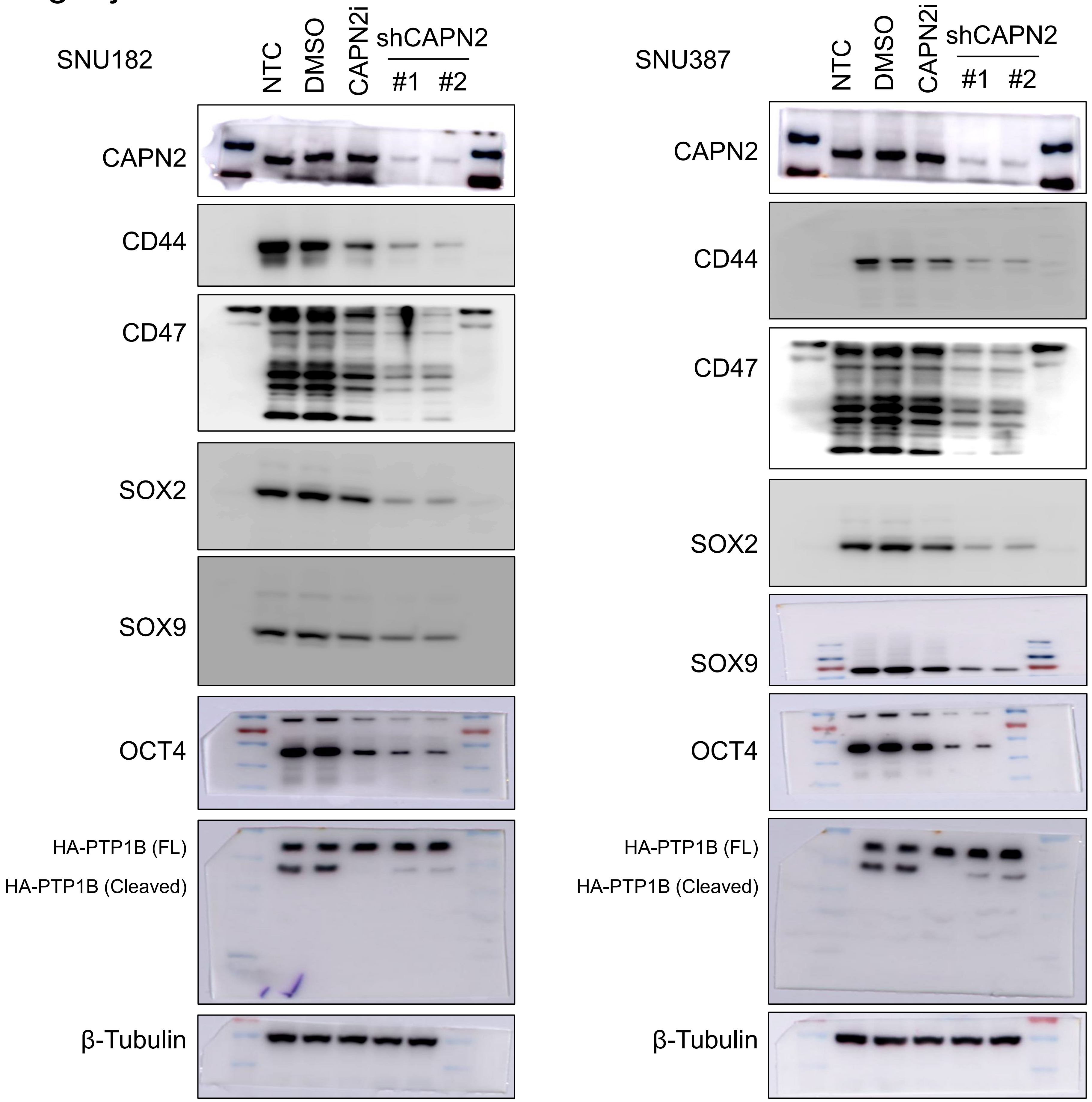

SNU182
NTC
DMSO
CAPN2i
shCAPN2
#1
#2
CAPN2
CD44
CD47
SOX2
SOX9
OCT4
HA-PTP1B (FL)
HA-PTP1B (Cleaved)
β-Tubulin
SNU387
NTC
DMSO
CAPN2i
shCAPN2
#1
#2
CAPN2
CD44
CD47
SOX2
SOX9
OCT4
HA-PTP1B (FL)
HA-PTP1B (Cleaved)
β-Tubulin

Fig. 4d

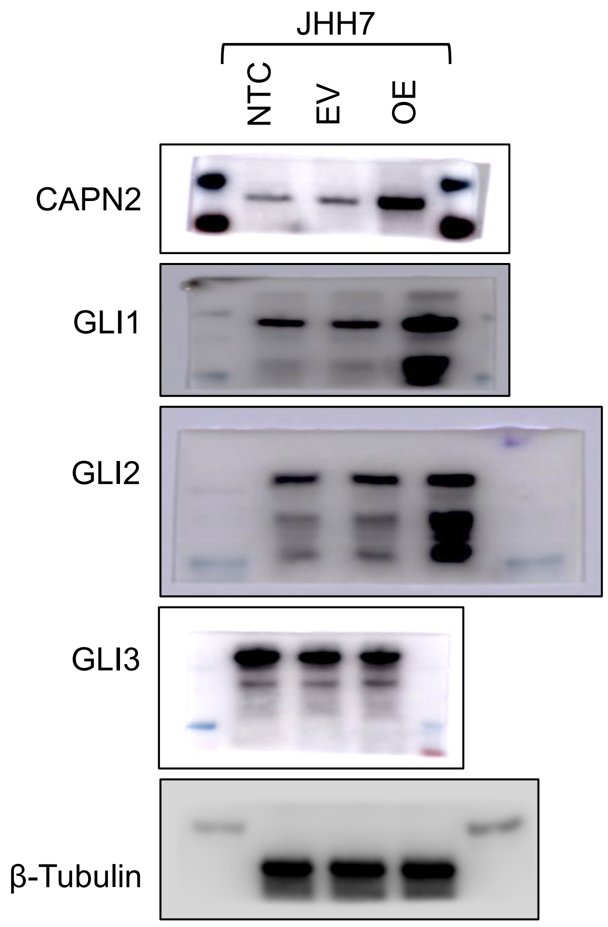

Fig. 4e

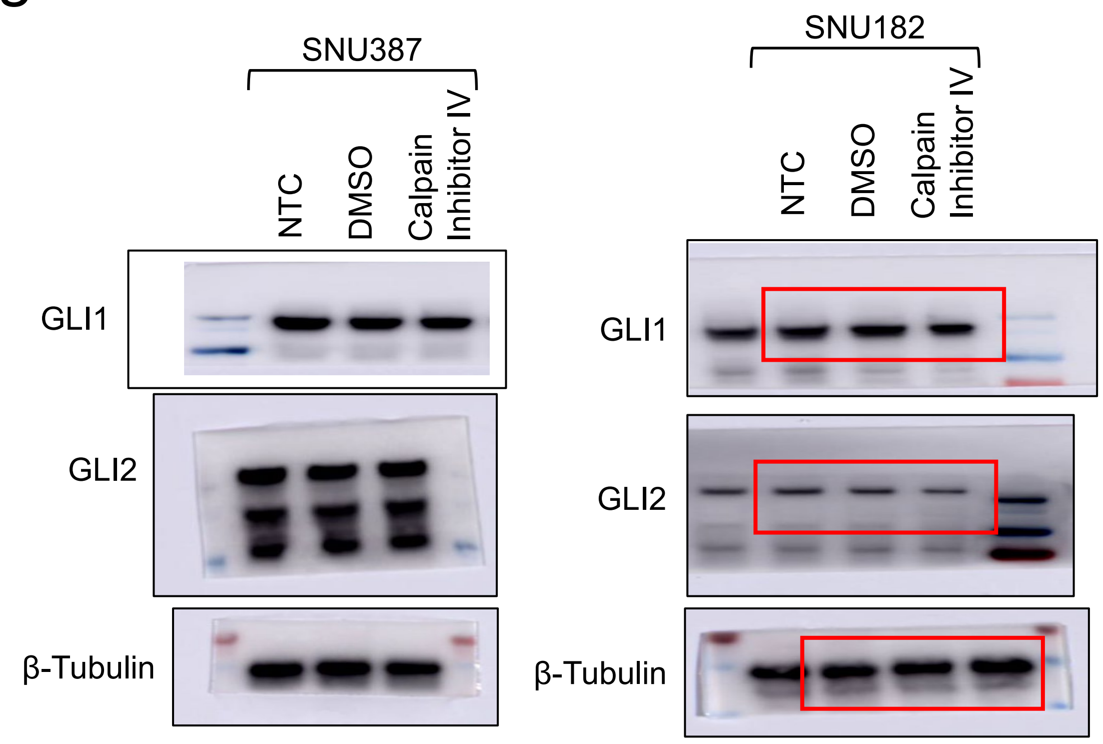

Fig. 4c

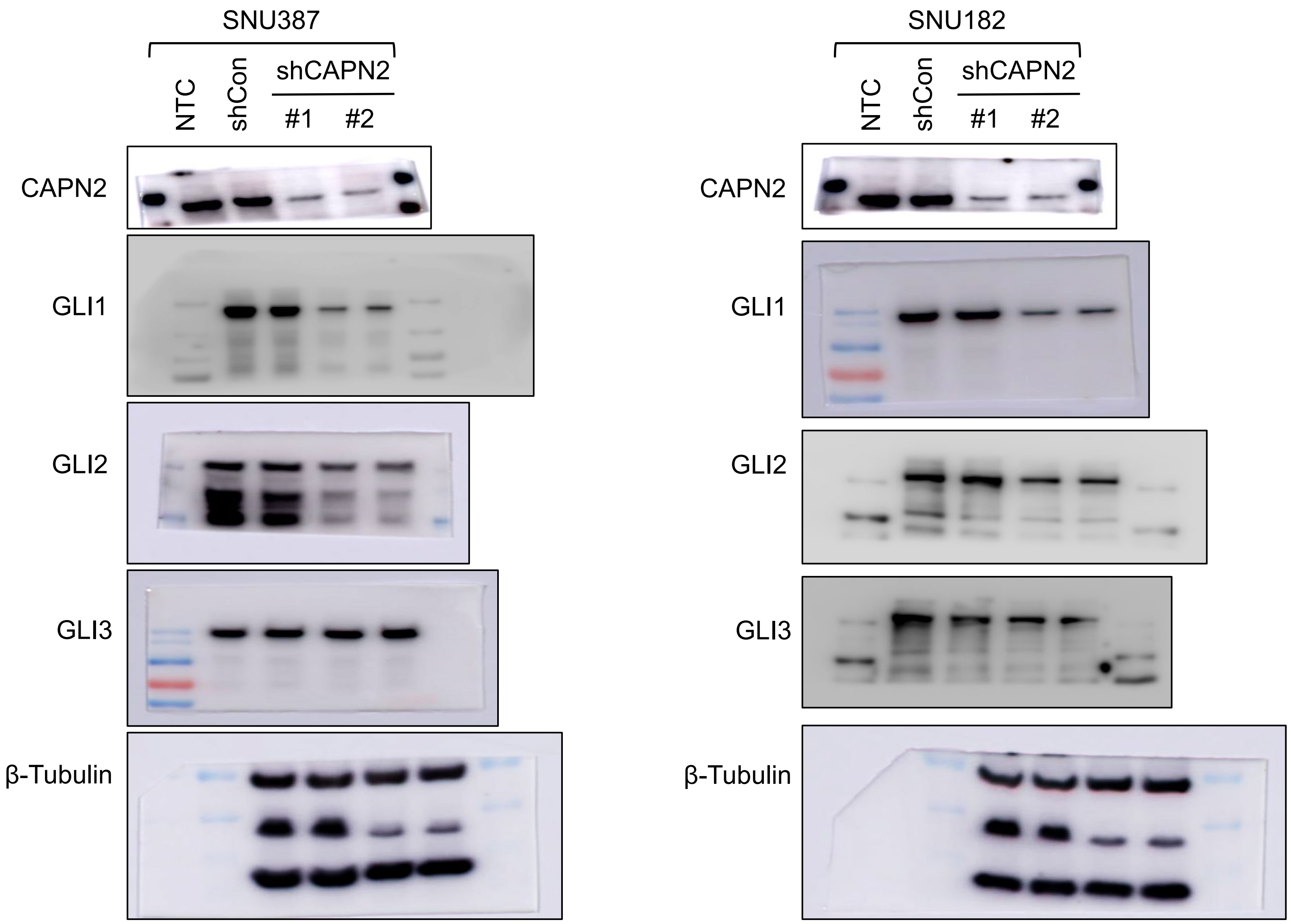

Fig. 4f

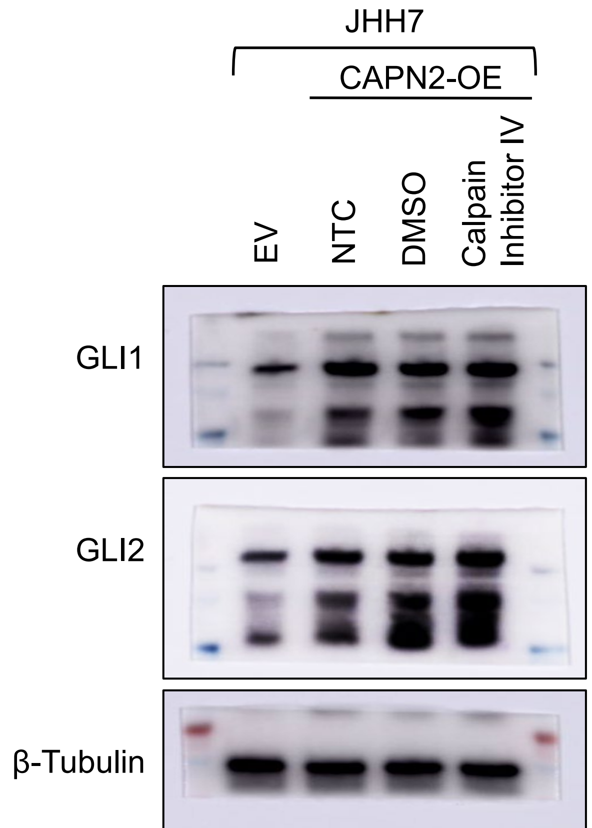

Fig. 4i

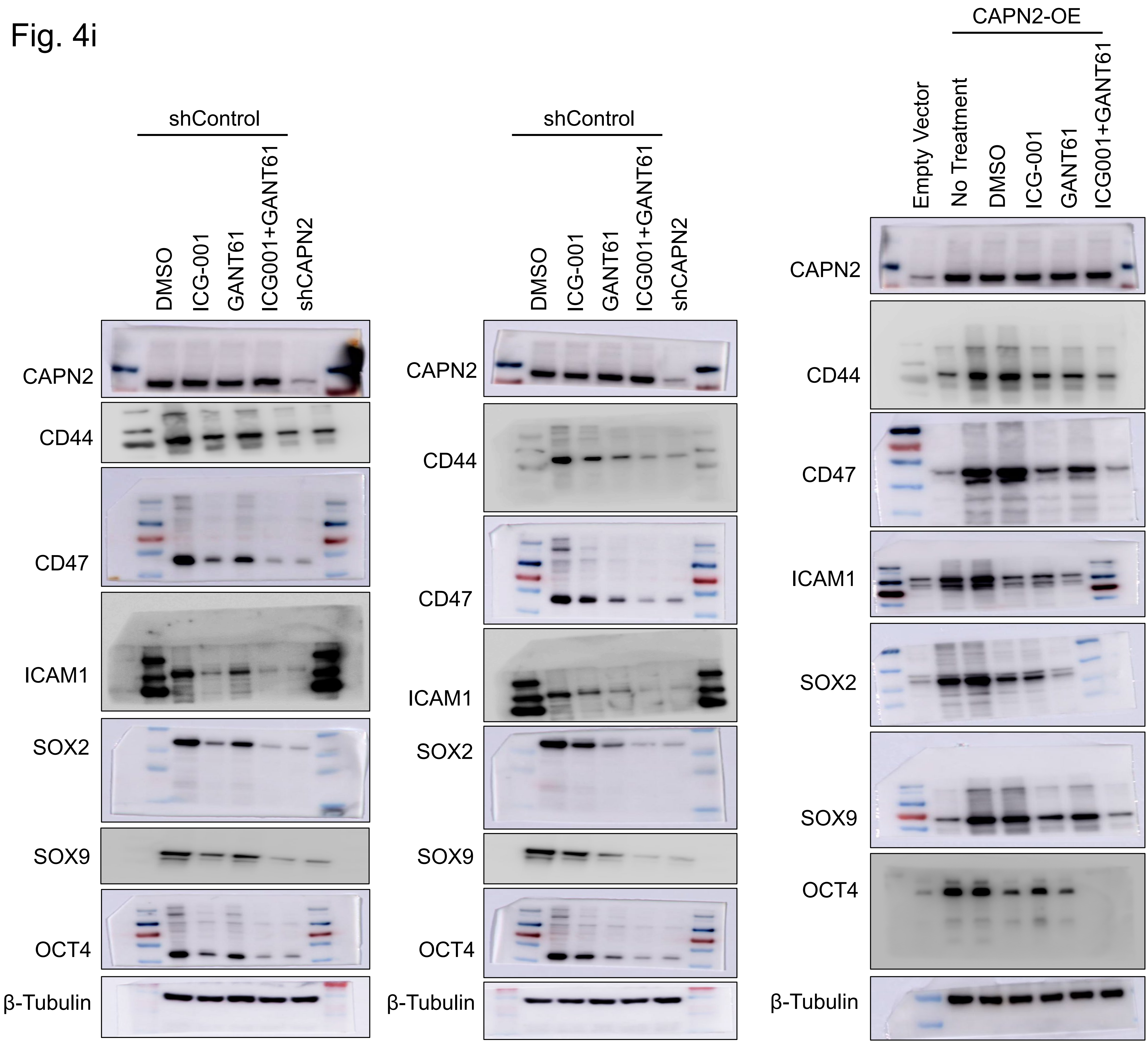

shControl
DMSO
ICG-001
GANT61
ICG001+GANT61
shCAPN2
CAPN2
CD44
CD47
ICAM1
SOX2
SOX9
OCT4
β-Tubulin
shControl
DMSO
ICG-001
GANT61
ICG001+GANT61
shCAPN2
CAPN2
CD44
CD47
ICAM1
SOX2
SOX9
OCT4
β-Tubulin
CAPN2-OE
Empty Vector
No Treatment
DMSO
ICG-001
GANT61
ICG001+GANT61
CAPN2
CD44
CD47
ICAM1
SOX2
SOX9
OCT4
β-Tubulin

Fig. 5a

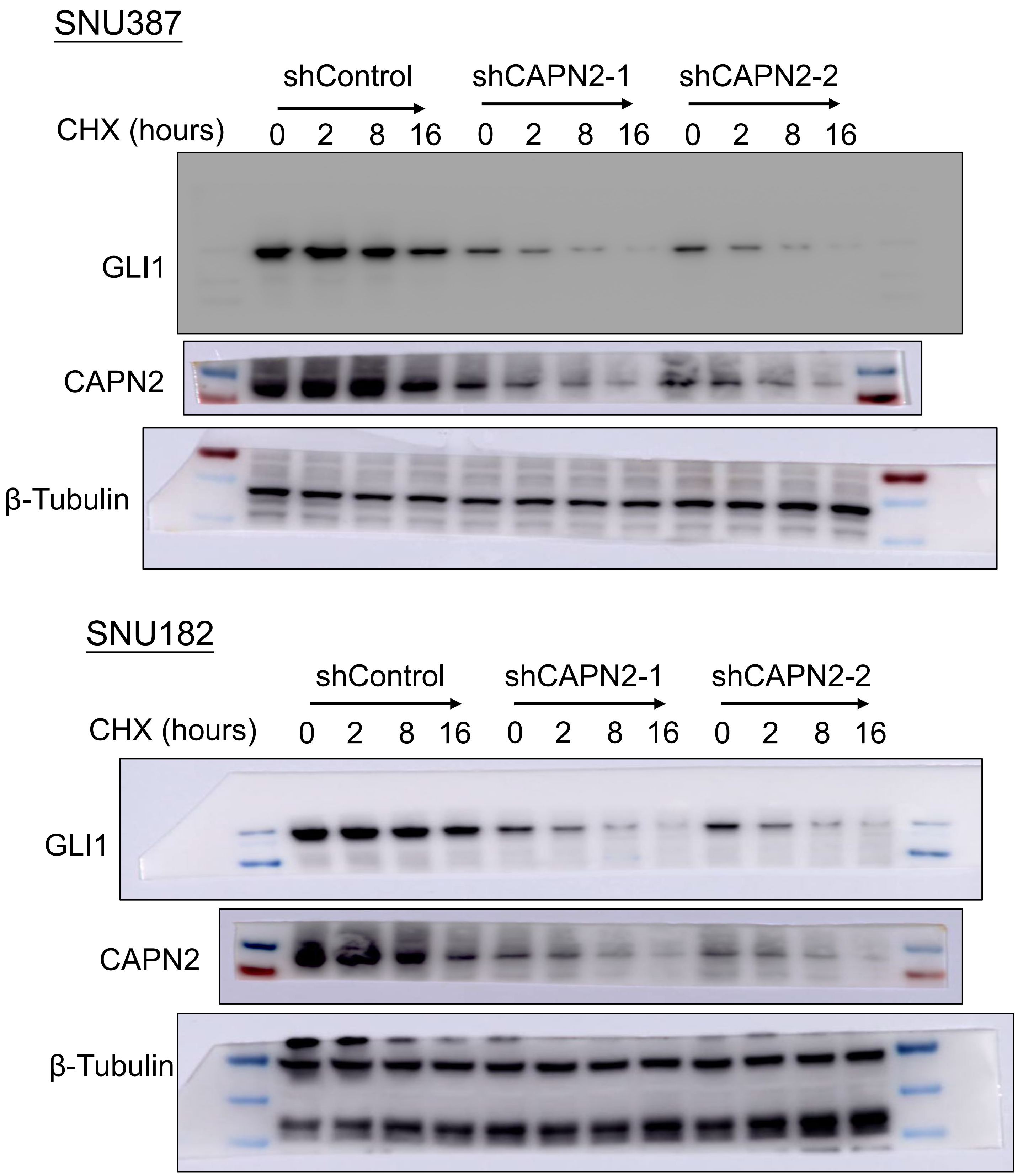
SNU387
shControl
shCAPN2-1
shCAPN2-2
CHX (hours)
0 2 8 16 0 2 8 16 0 2 8 16
GLI1
CAPN2
β-Tubulin
SNU182
shControl
shCAPN2-1
shCAPN2-2
CHX (hours)
0 2 8 16 0 2 8 16 0 2 8 16
GLI1
CAPN2
β-Tubulin

Fig. 5b

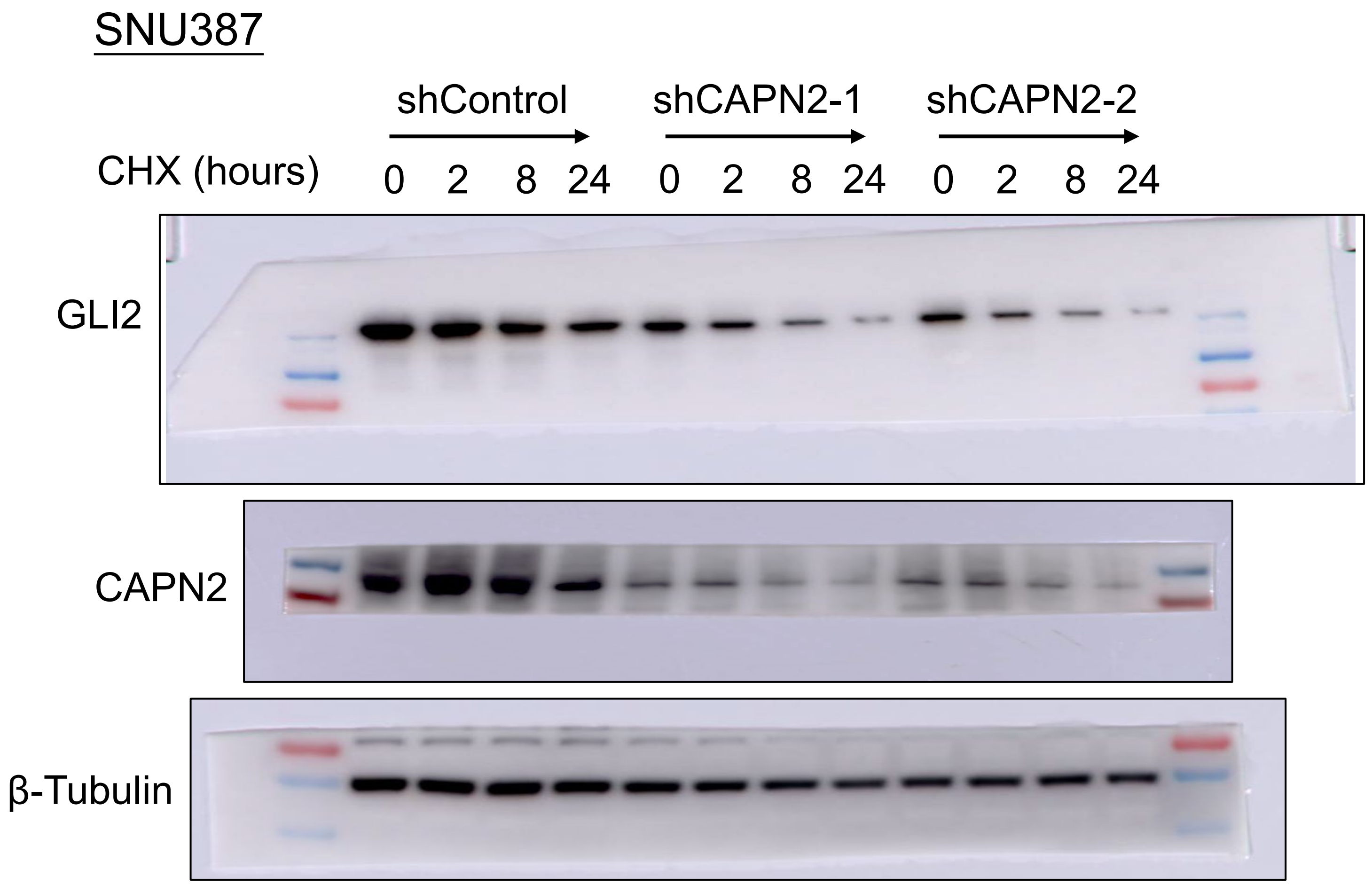
SNU387
shControl
shCAPN2-1
shCAPN2-2
CHX (hours)
0 2 8 24 0 2 8 24 0 2 8 24
GLI2
CAPN2
β-Tubulin

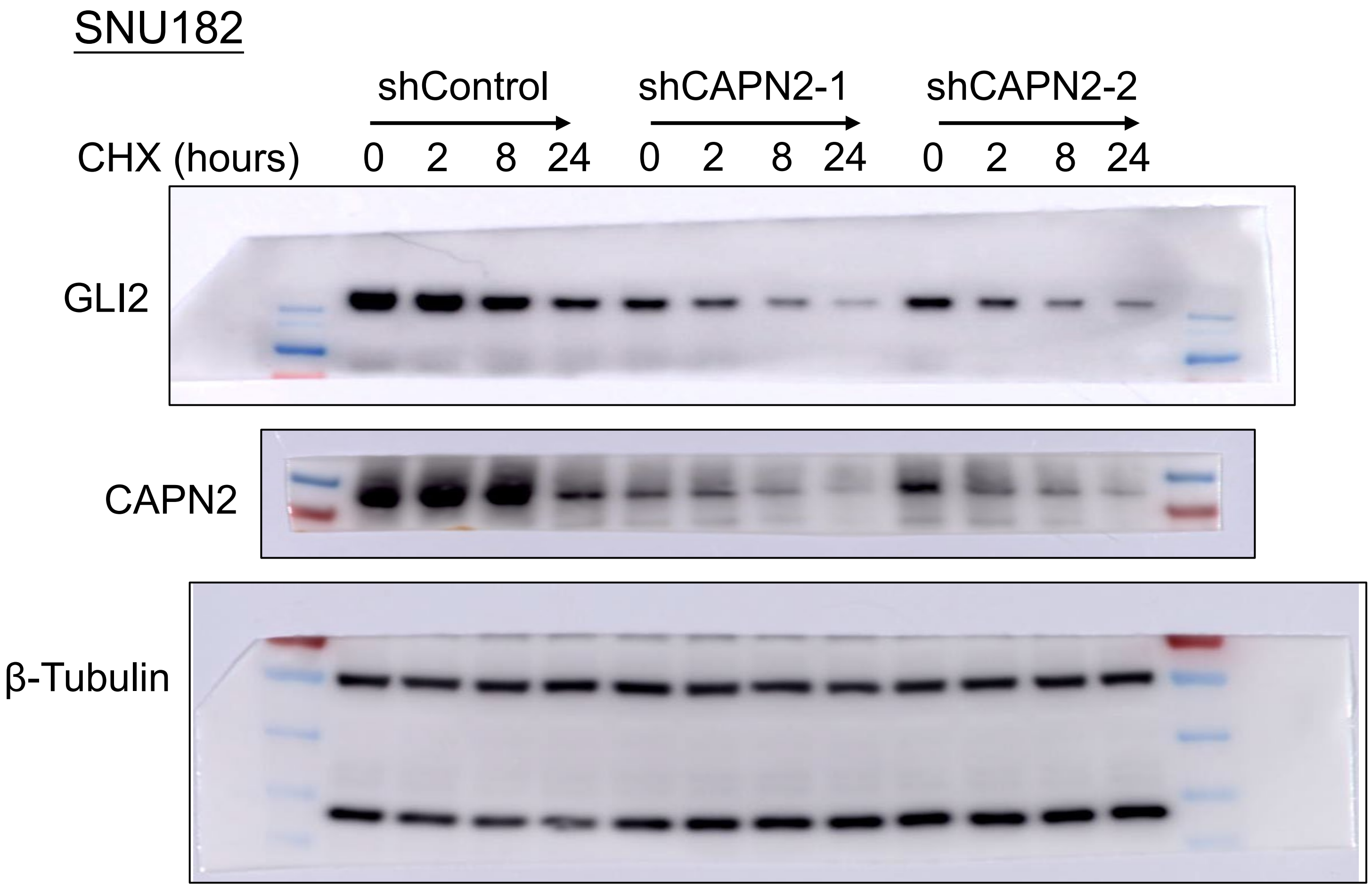
SNU182
shControl
shCAPN2-1
shCAPN2-2
CHX (hours)
0 2 8 24 0 2 8 24 0 2 8 24
GLI2
CAPN2
β-Tubulin

Fig. 5c

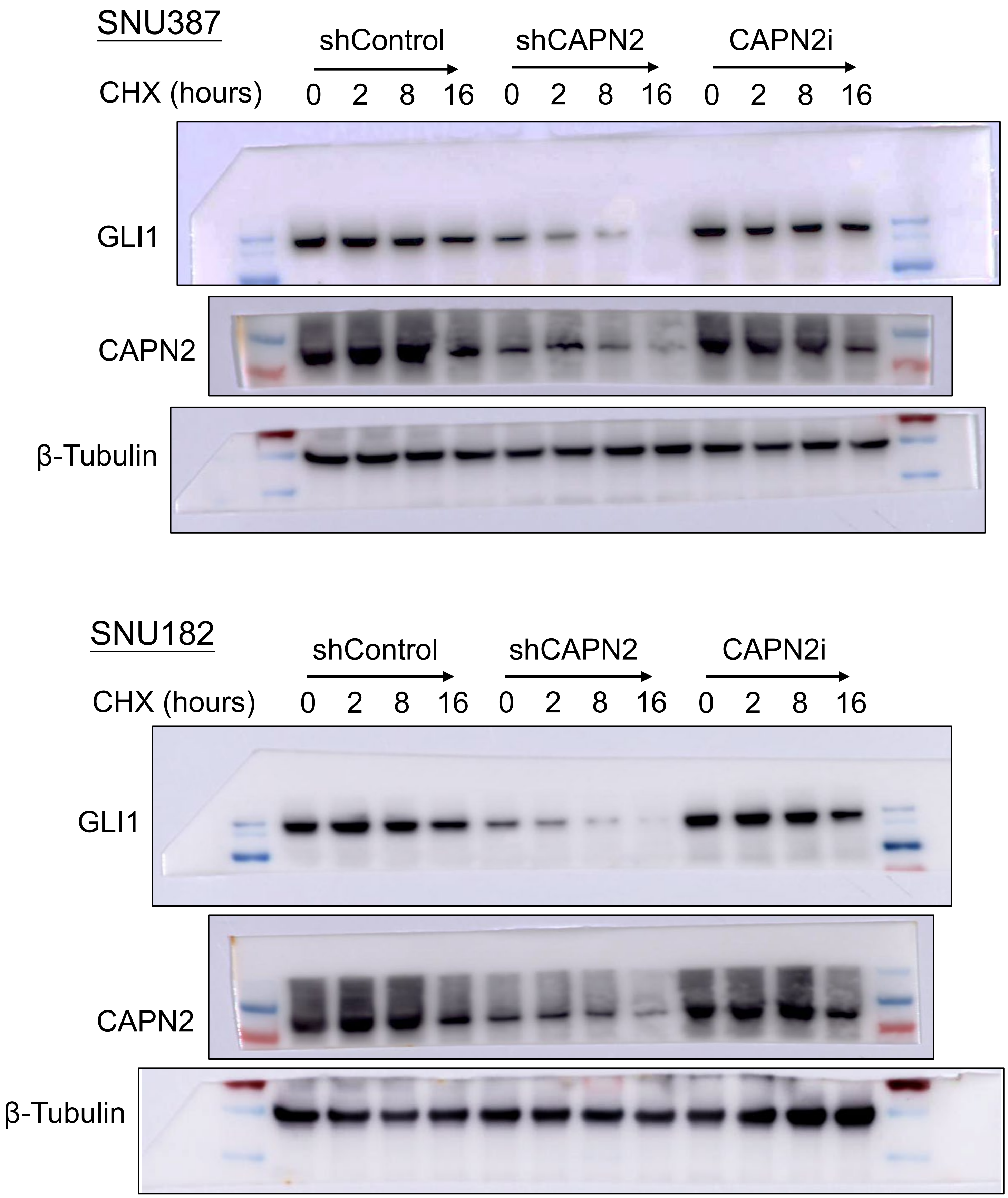

SNU387
shControl
shCAPN2
CAPN2i
CHX (hours)
0 2 8 16 0 2 8 16 0 2 8 16
GLI1
CAPN2
β-Tubulin
SNU182
shControl
shCAPN2
CAPN2i
CHX (hours)
0 2 8 16 0 2 8 16 0 2 8 16
GLI1
CAPN2
β-Tubulin

Fig. 5d

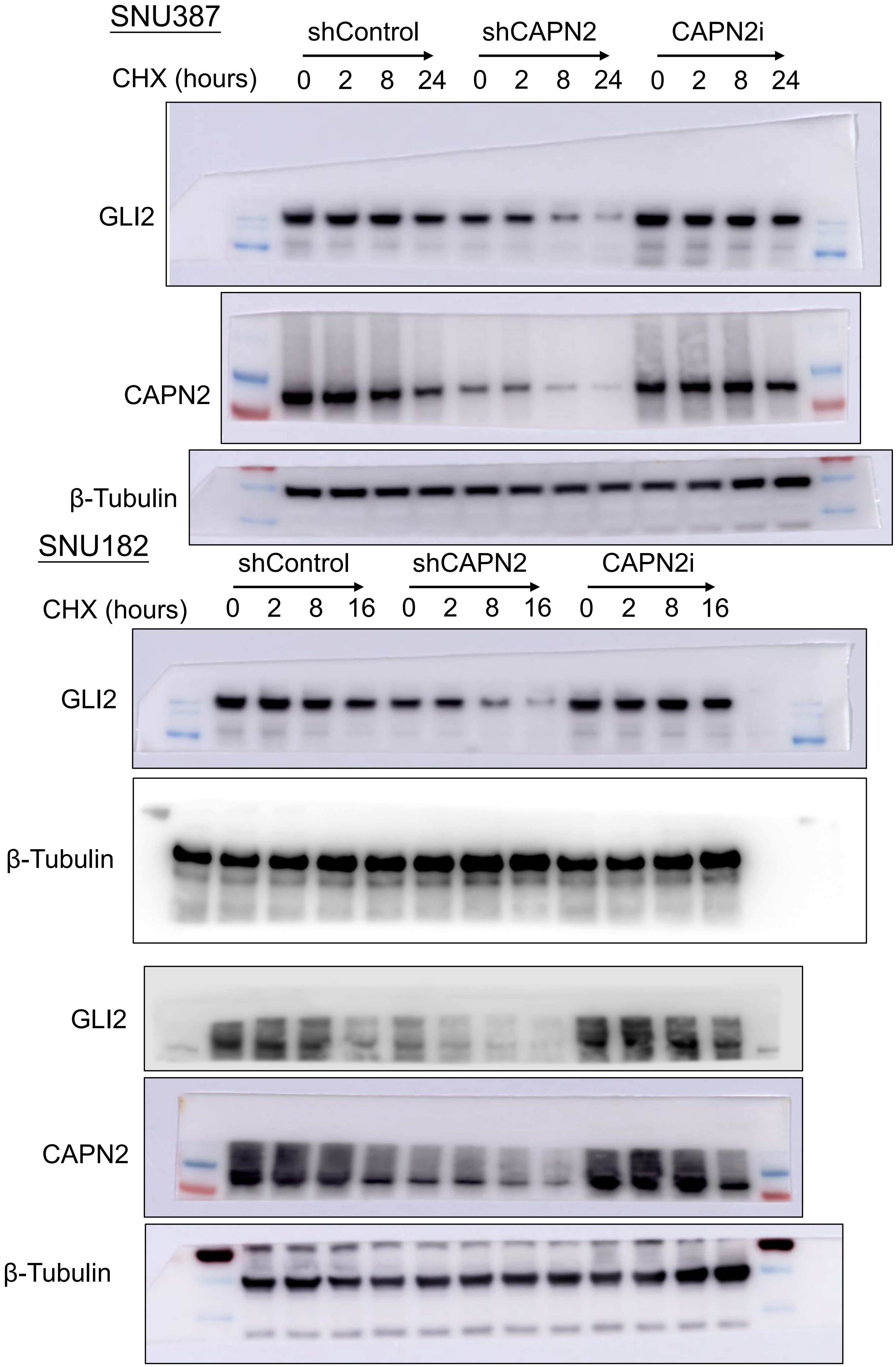
SNU387
shControl
shCAPN2
CAPN2i
CHX (hours)
0 2 8 24 0 2 8 24 0 2 8 24
GLI2
CAPN2
β-Tubulin
SNU182
shControl
shCAPN2
CAPN2i
CHX (hours)
0 2 8 16 0 2 8 16 0 2 8 16
GLI2
β-Tubulin
GLI2
CAPN2
β-Tubulin

Fig. 5e

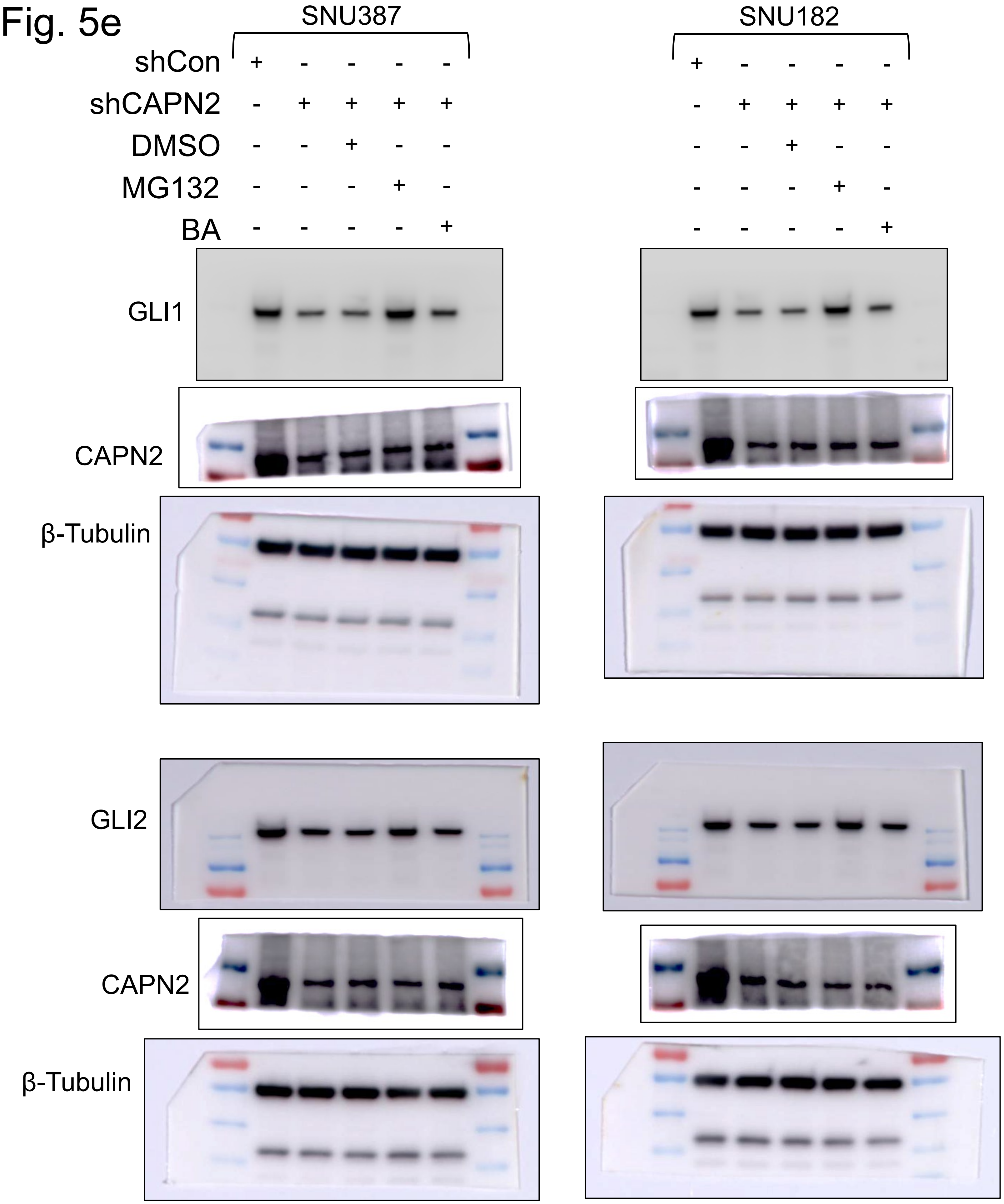
SNU387
SNU182
shCon + - - - -
shCAPN2 - + + + +
DMSO - - + - -
MG132 - - - + -
BA - - - - +
GLI1
CAPN2
β-Tubulin
GLI2
CAPN2
β-Tubulin

Fig. 5f

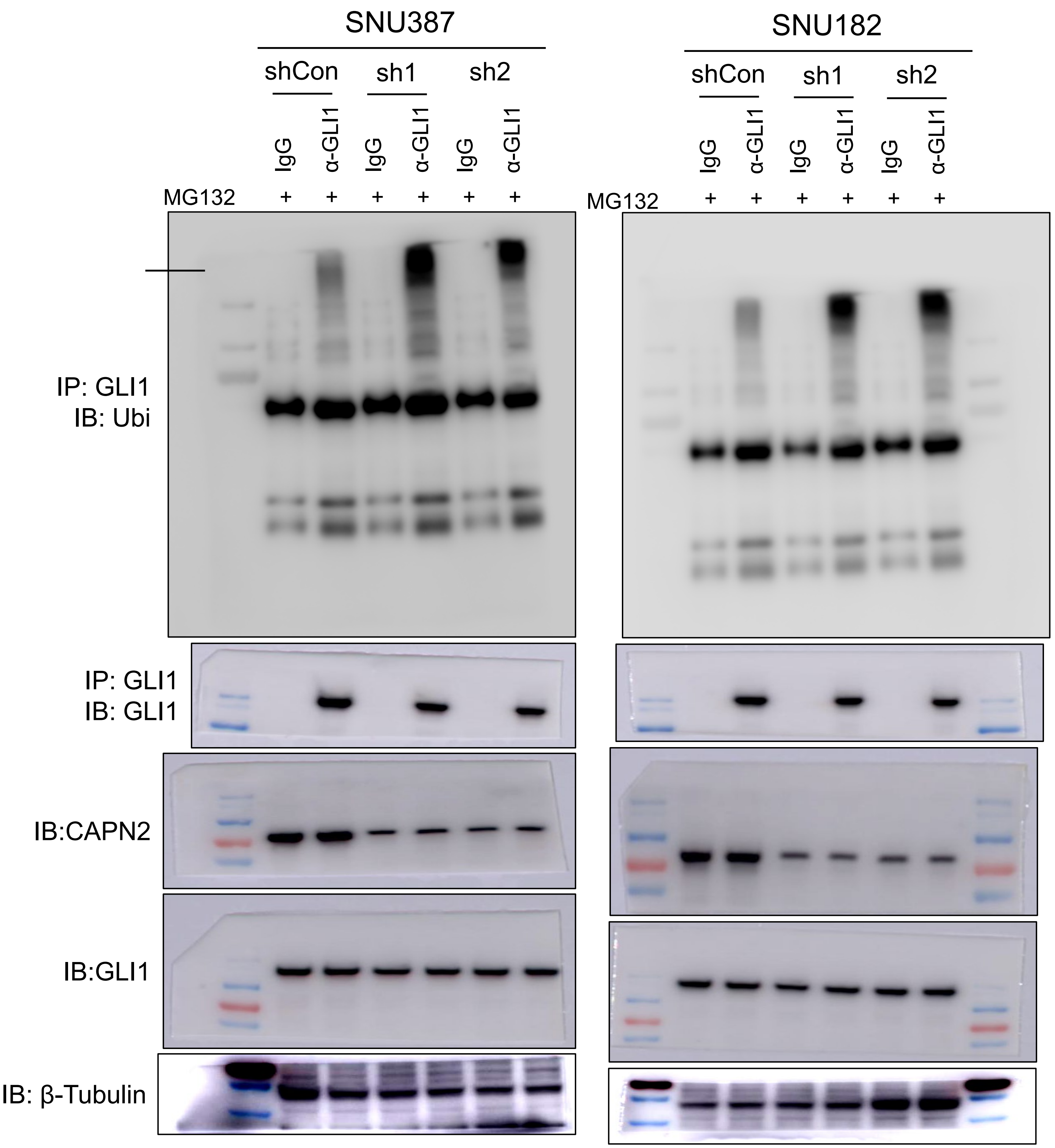
SNU387
shCon
sh1
sh2
IgG
α-GLI1
IgG
α-GLI1
IgG
α-GLI1
MG132
+
+
+
+
+
+
SNU182
shCon
sh1
sh2
IgG
α-GLI1
IgG
α-GLI1
IgG
α-GLI1
MG132
+
+
+
+
+
+
IP: GLI1
IB: Ubi
IP: GLI1
IB: GLI1
IB:CAPN2
IB:GLI1
IB: β-Tubulin

Fig. 5g

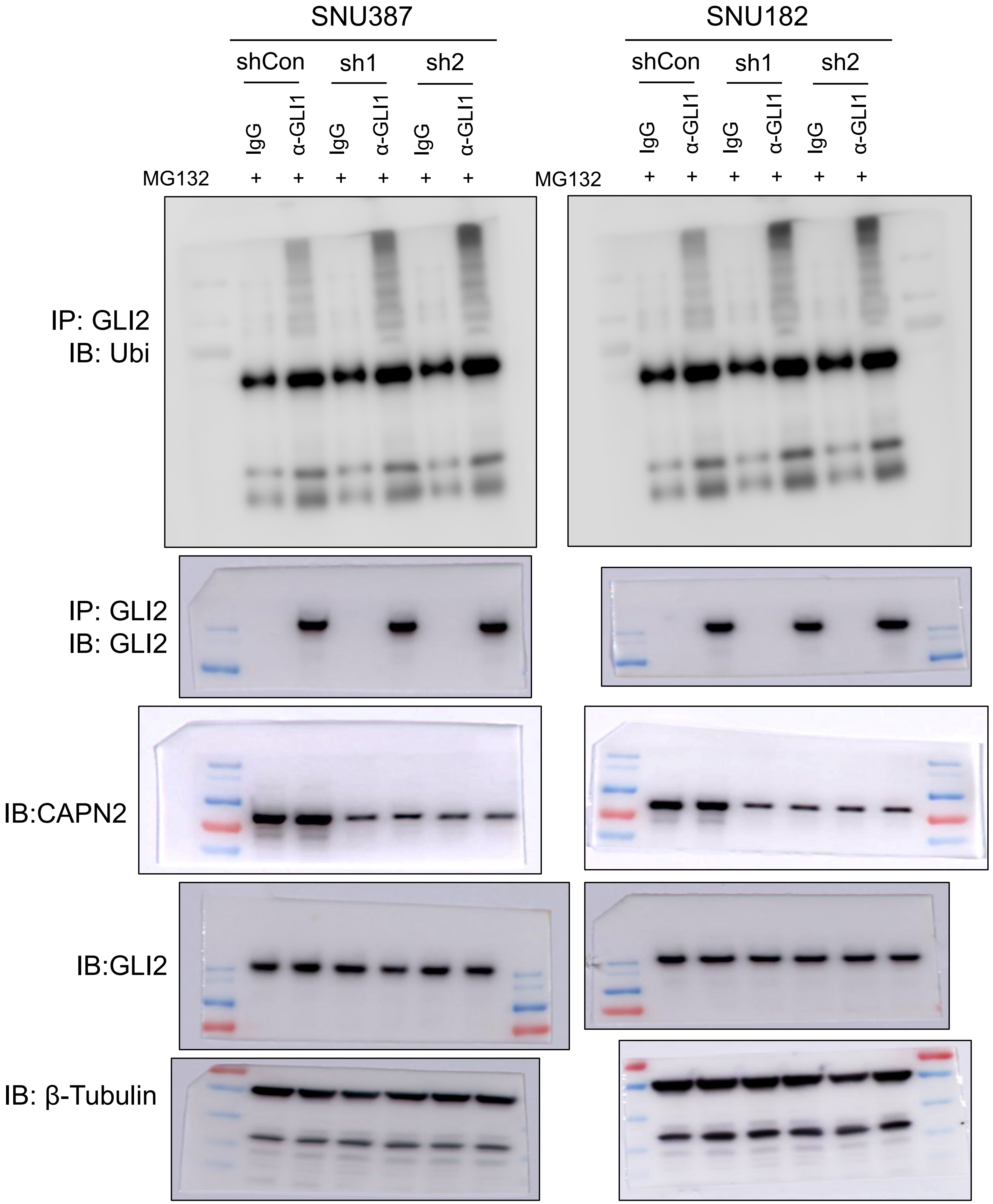

SNU387
shCon
sh1
sh2
IgG
α-GLI1
IgG
α-GLI1
IgG
α-GLI1
MG132
+ + + + + +
SNU182
shCon
sh1
sh2
IgG
α-GLI1
IgG
α-GLI1
IgG
α-GLI1
MG132
+ + + + + +
IP: GLI2
IB: Ubi
IP: GLI2
IB: GLI2
IB:CAPN2
IB:GLI2
IB: β-Tubulin

Fig. 5h

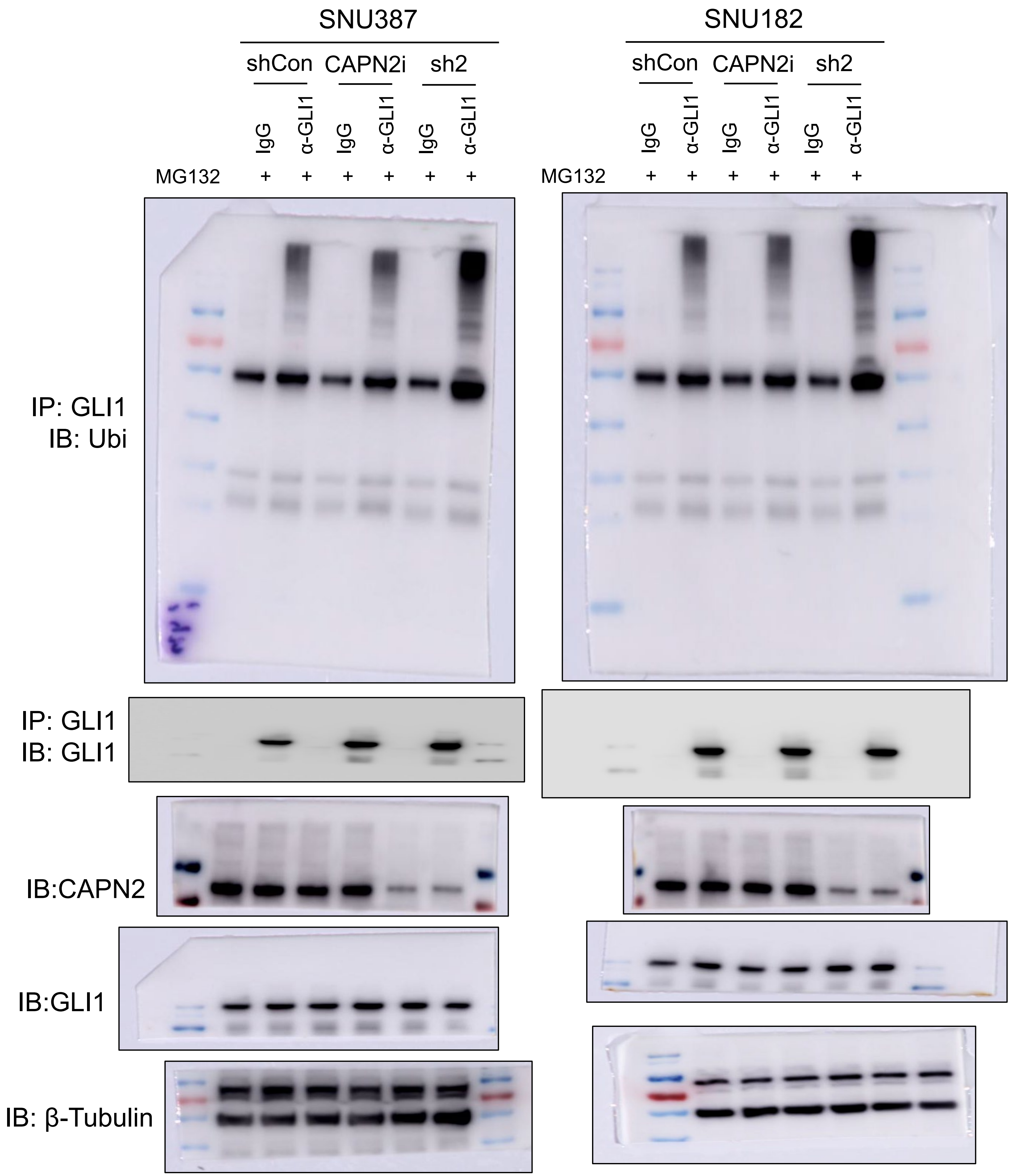
SNU387
shCon
CAPN2i
sh2
IgG
α-GLI1
IgG
α-GLI1
IgG
α-GLI1
MG132
+ + + + + +
SNU182
shCon
CAPN2i
sh2
IgG
α-GLI1
IgG
α-GLI1
IgG
α-GLI1
MG132
+ + + + + +
IP: GLI1
IB: Ubi
IP: GLI1
IB: GLI1
IB:CAPN2
IB:GLI1
IB: β-Tubulin

Fig. 5i

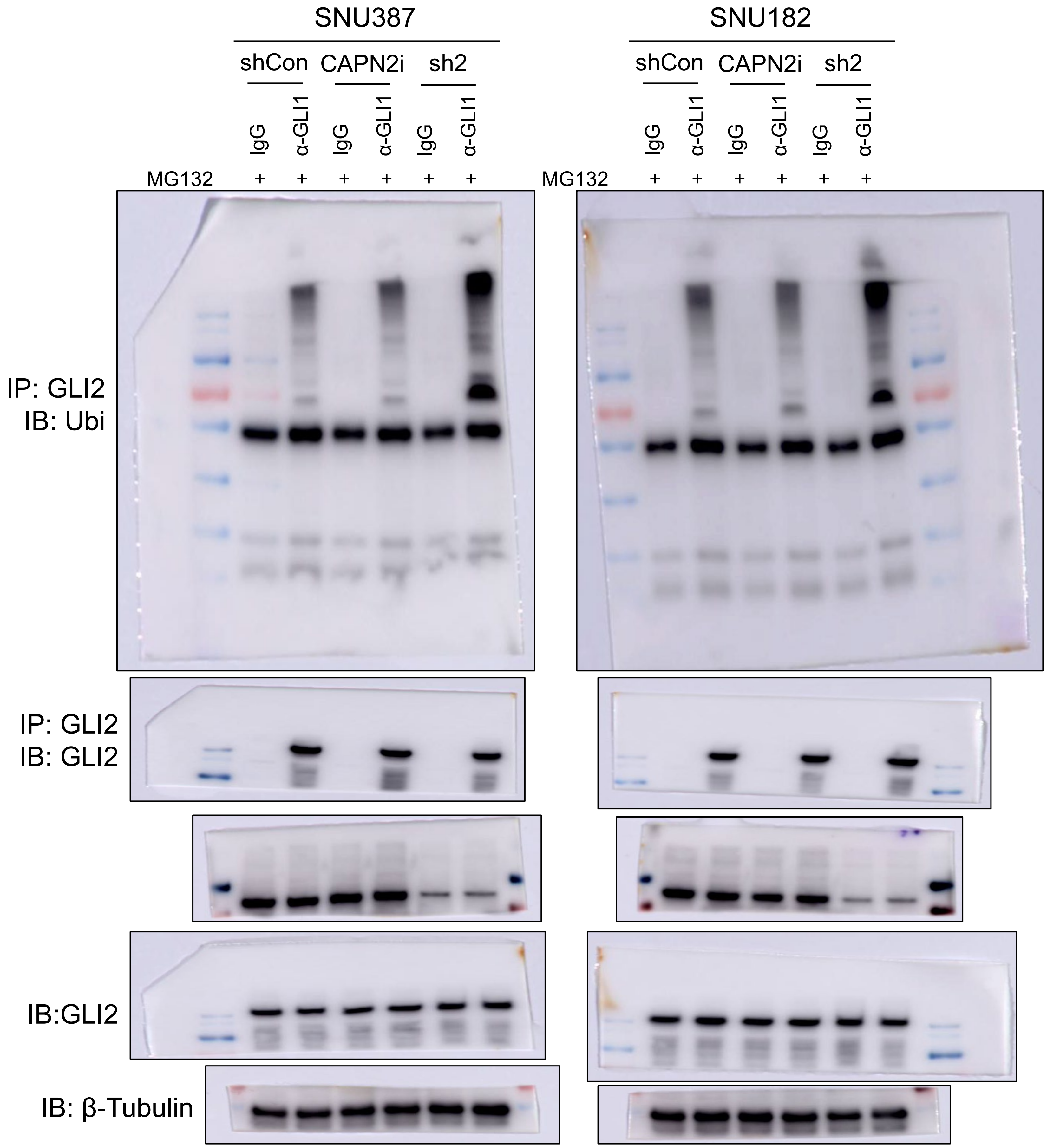
SNU387
shCon
CAPN2i
sh2
IgG
α-GLI1
MG132
+
SNU182
IP: GLI2
IB: Ubi
IP: GLI2
IB: GLI2
IB:GLI2
IB: β-Tubulin

Fig. 6b

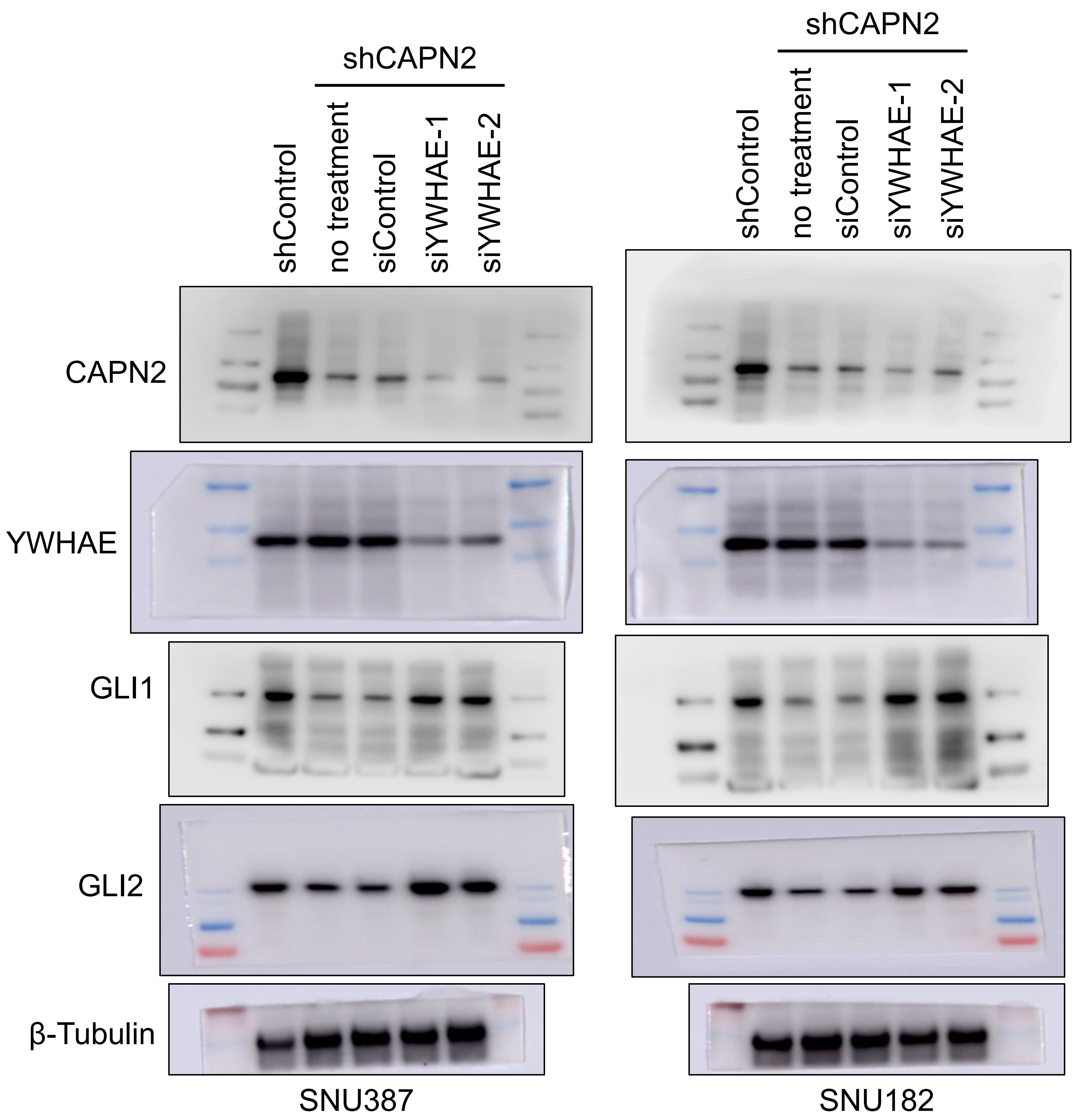

shCAPN2
shControl
no treatment
siControl
siYWHAE-1
siYWHAE-2
CAPN2
YWHAE
GLI1
GLI2
β-Tubulin
SNU387
SNU182

Fig. 6c

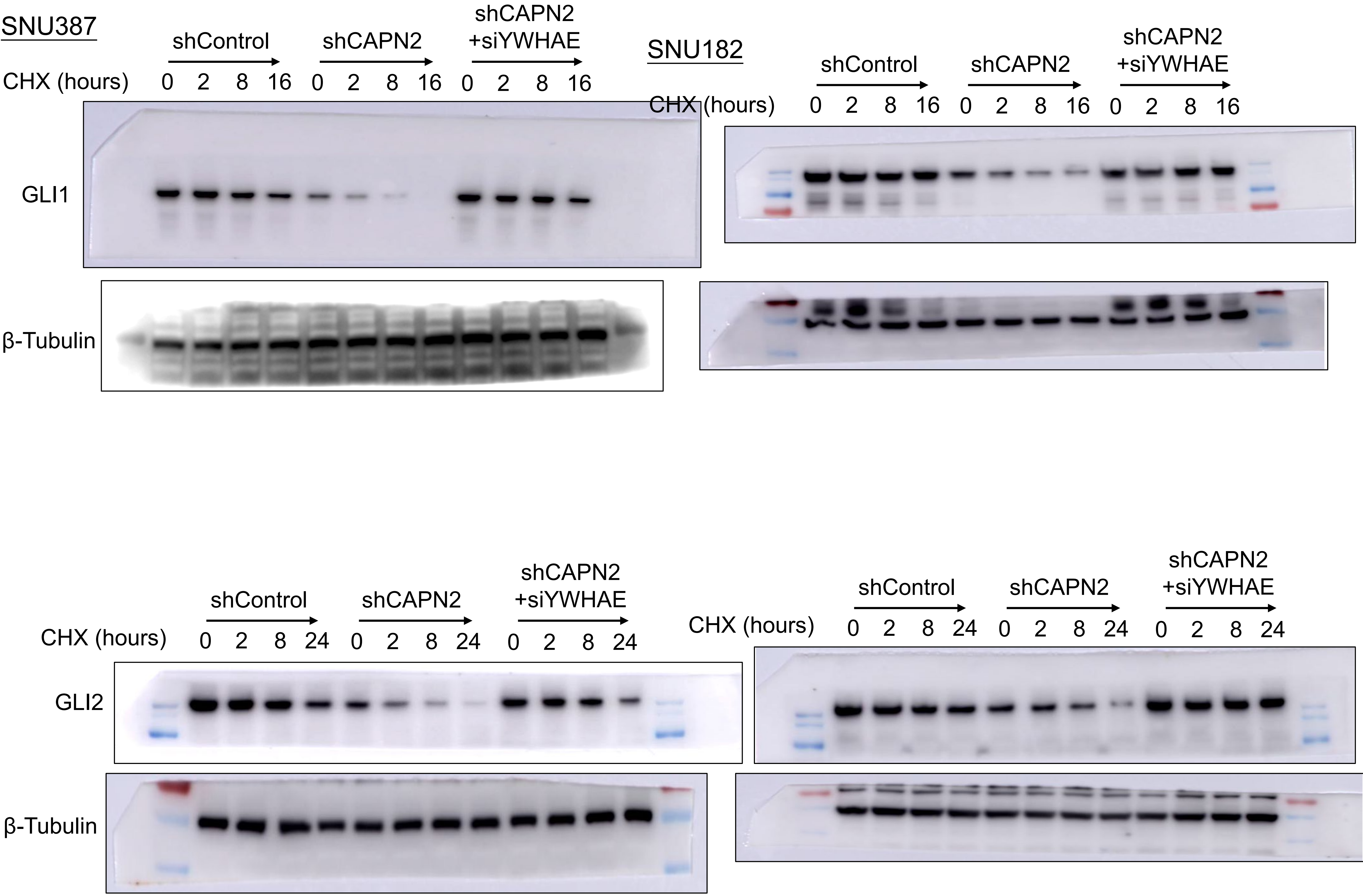
SNU387
shControl
shCAPN2
shCAPN2
+siYWHAE
CHX (hours)
0 2 8 16 0 2 8 16 0 2 8 16
GLI1
β-Tubulin
SNU182
shControl
shCAPN2
shCAPN2
+siYWHAE
CHX (hours)
0 2 8 16 0 2 8 16 0 2 8 16
shControl
shCAPN2
shCAPN2
+siYWHAE
CHX (hours)
0 2 8 24 0 2 8 24 0 2 8 24
GLI2
β-Tubulin
shControl
shCAPN2
shCAPN2
+siYWHAE
CHX (hours)
0 2 8 24 0 2 8 24 0 2 8 24

Fig. 6d

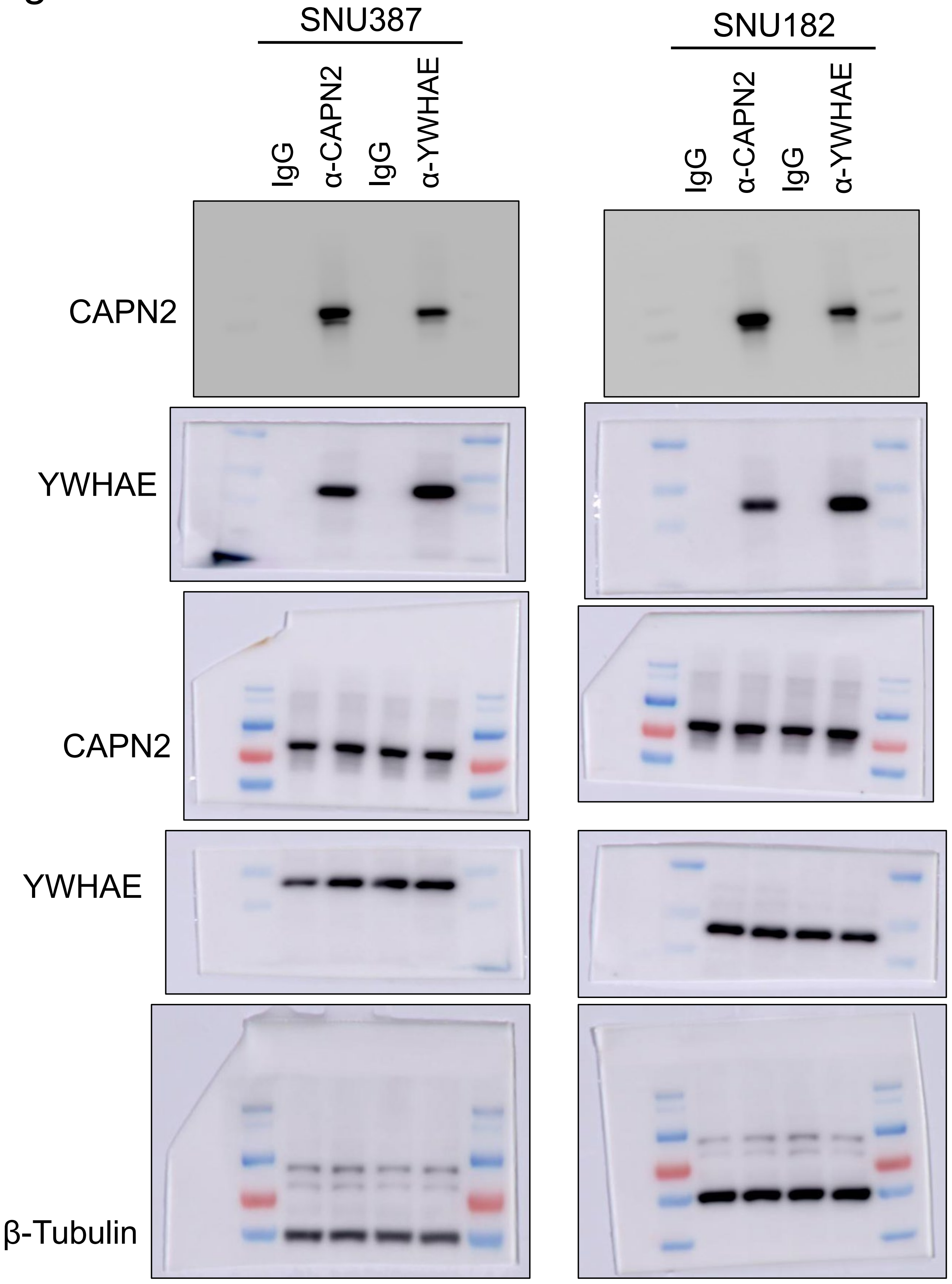
SNU387
SNU182
IgG
α-CAPN2
IgG
α-YWHAE
CAPN2
YWHAE
CAPN2
YWHAE
β-Tubulin

Fig. 6e

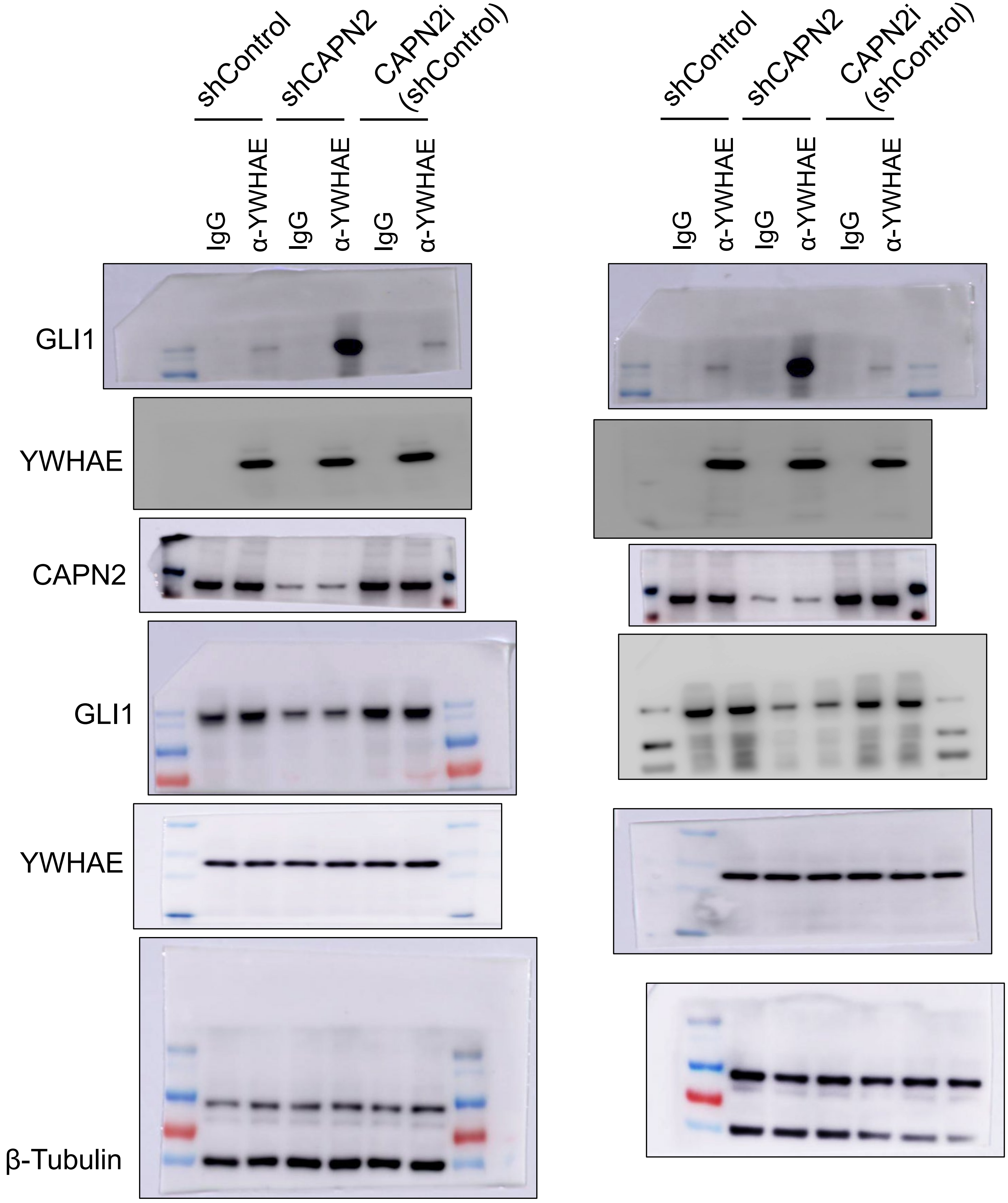
shControl
shCAPN2
CAPN2i (shControl)
IgG
α-YWHAE
IgG
α-YWHAE
IgG
α-YWHAE
GLI1
YWHAE
CAPN2
GLI1
YWHAE
β-Tubulin

Fig. 6f

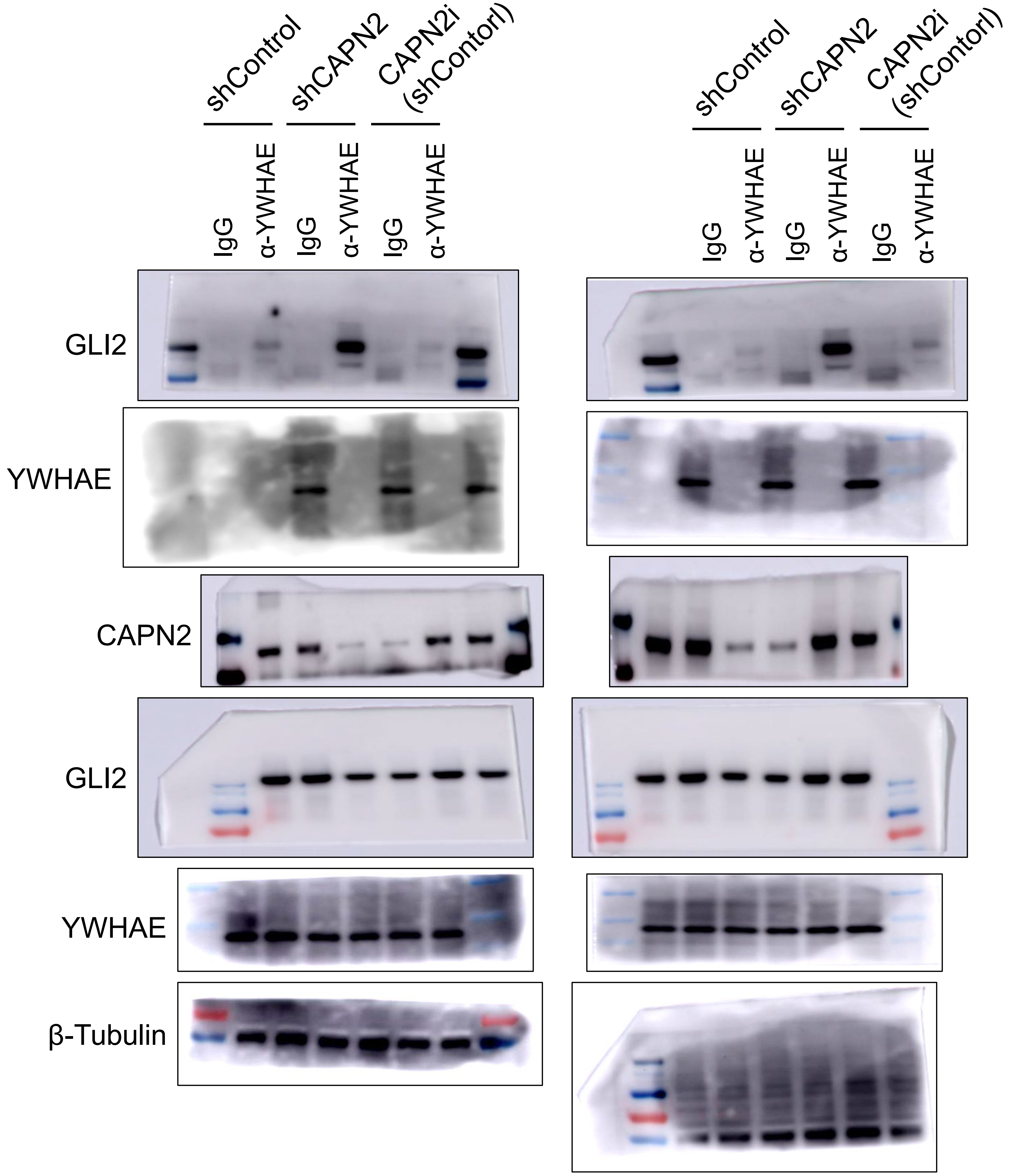
shControl
shCAPN2
CAPN2i (shContorl)
IgG
α-YWHAE
IgG
α-YWHAE
IgG
α-YWHAE
shControl
shCAPN2
CAPN2i (shContorl)
IgG
α-YWHAE
IgG
α-YWHAE
IgG
α-YWHAE
GLI2
YWHAE
CAPN2
GLI2
YWHAE
β-Tubulin

Fig. 6g

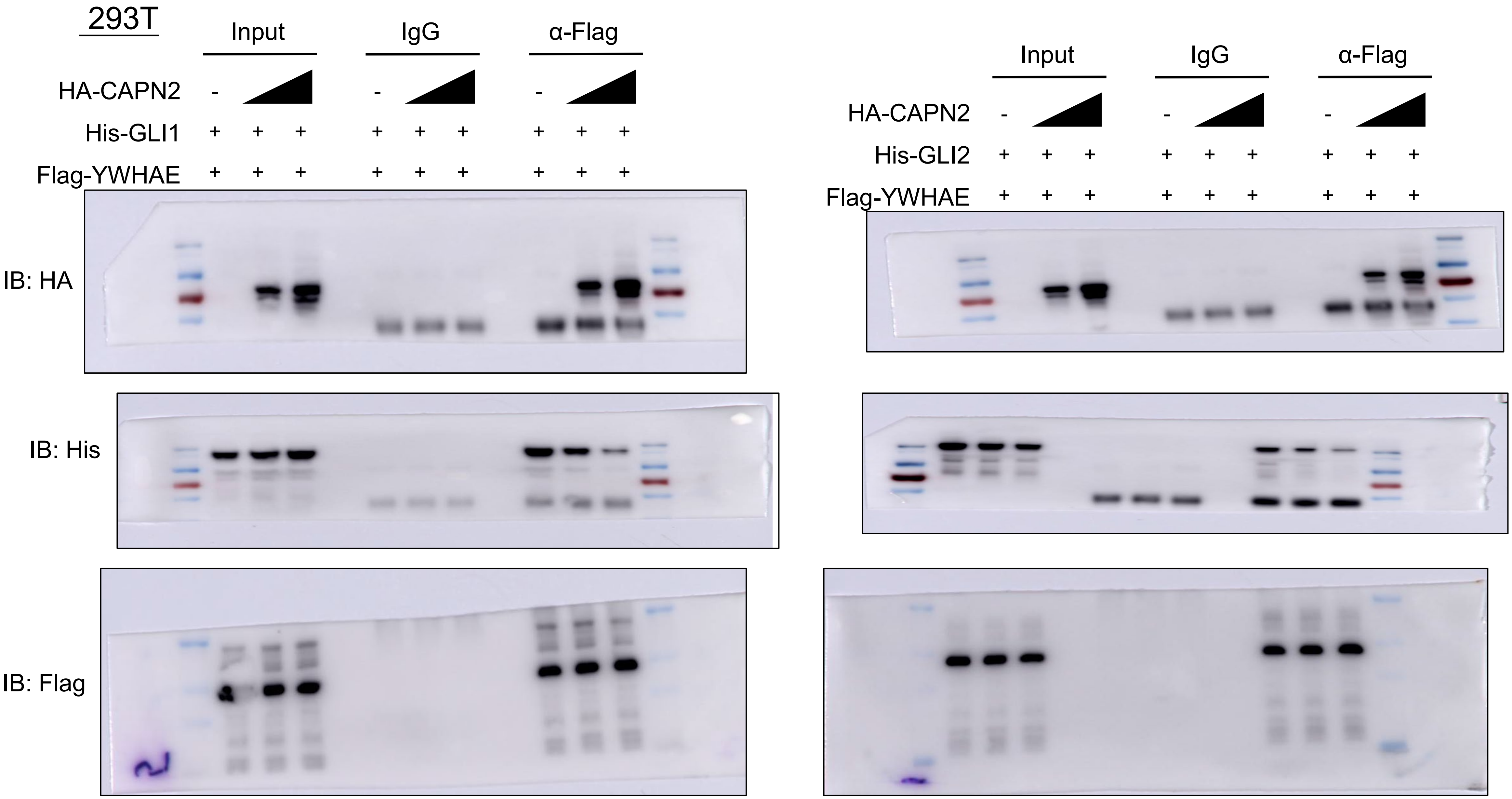
293T
Input
IgG
α-Flag
HA-CAPN2
His-GLI1
Flag-YWHAE
IB: HA
IB: His
IB: Flag
Input
IgG
α-Flag
HA-CAPN2
His-GLI2
Flag-YWHAE

Fig. 6h

Fig. 7e

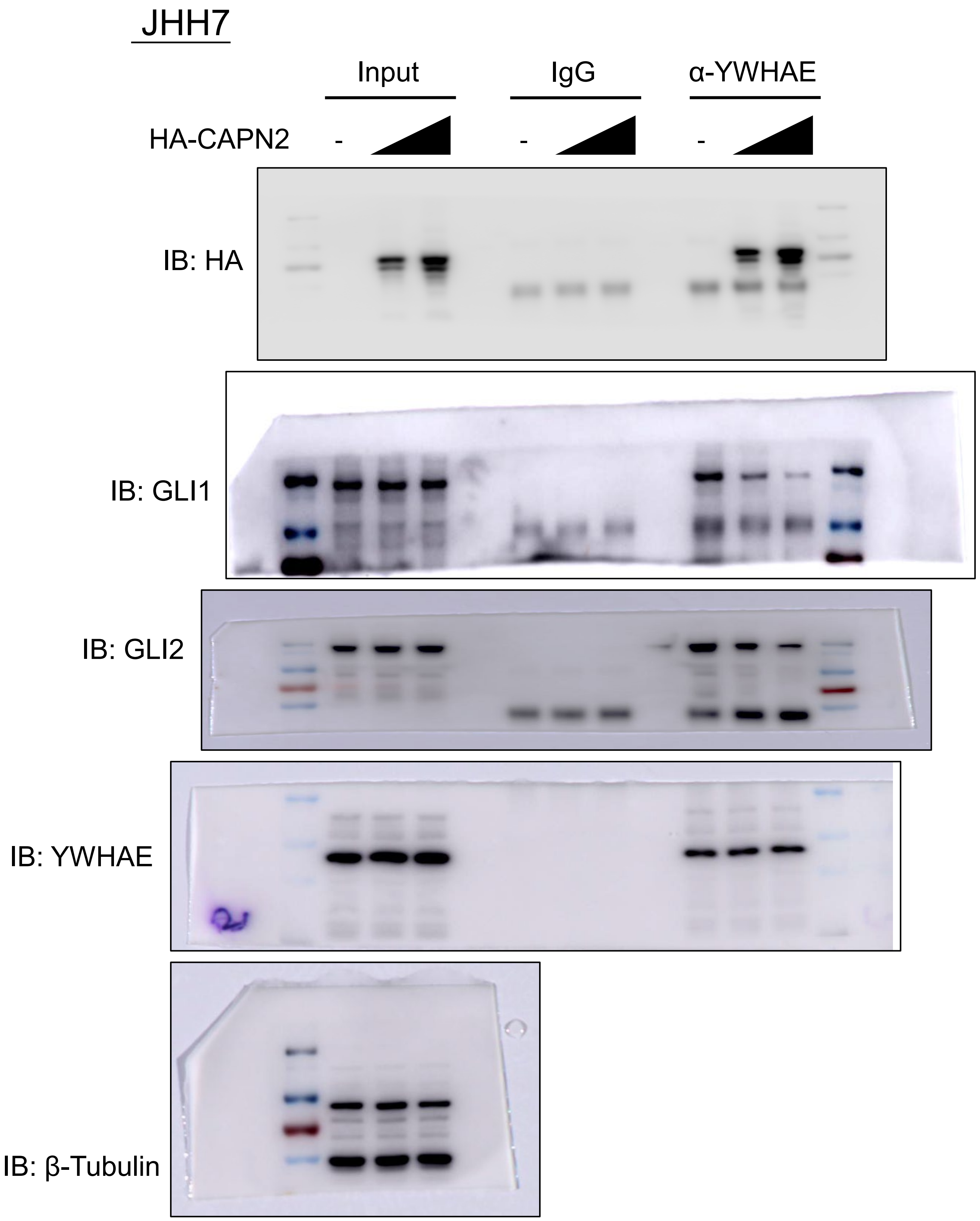
JHH7
Input
IgG
α-YWHAE
HA-CAPN2
-
-
-
IB: HA
IB: GLI1
IB: GLI2
IB: YWHAE
IB: β-Tubulin

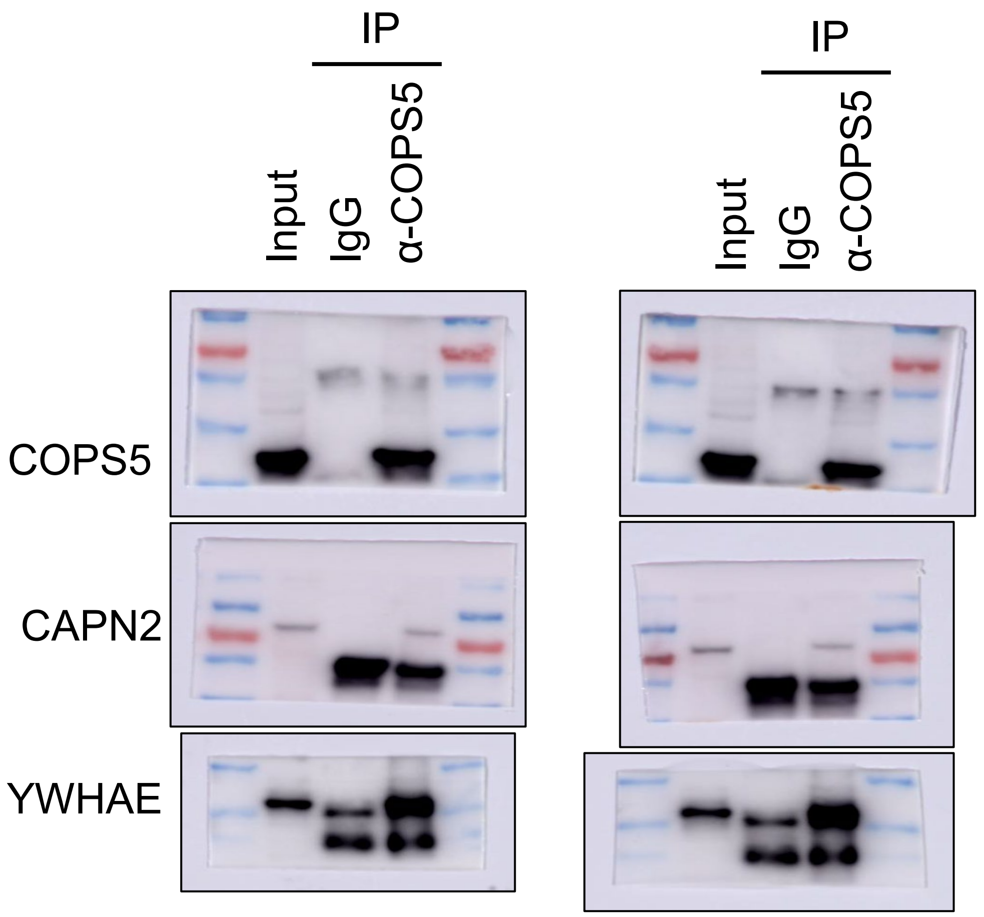
IP
Input
IgG
α-COPS5
IP
Input
IgG
α-COPS5
COPS5
CAPN2
YWHAE

Fig. 6i

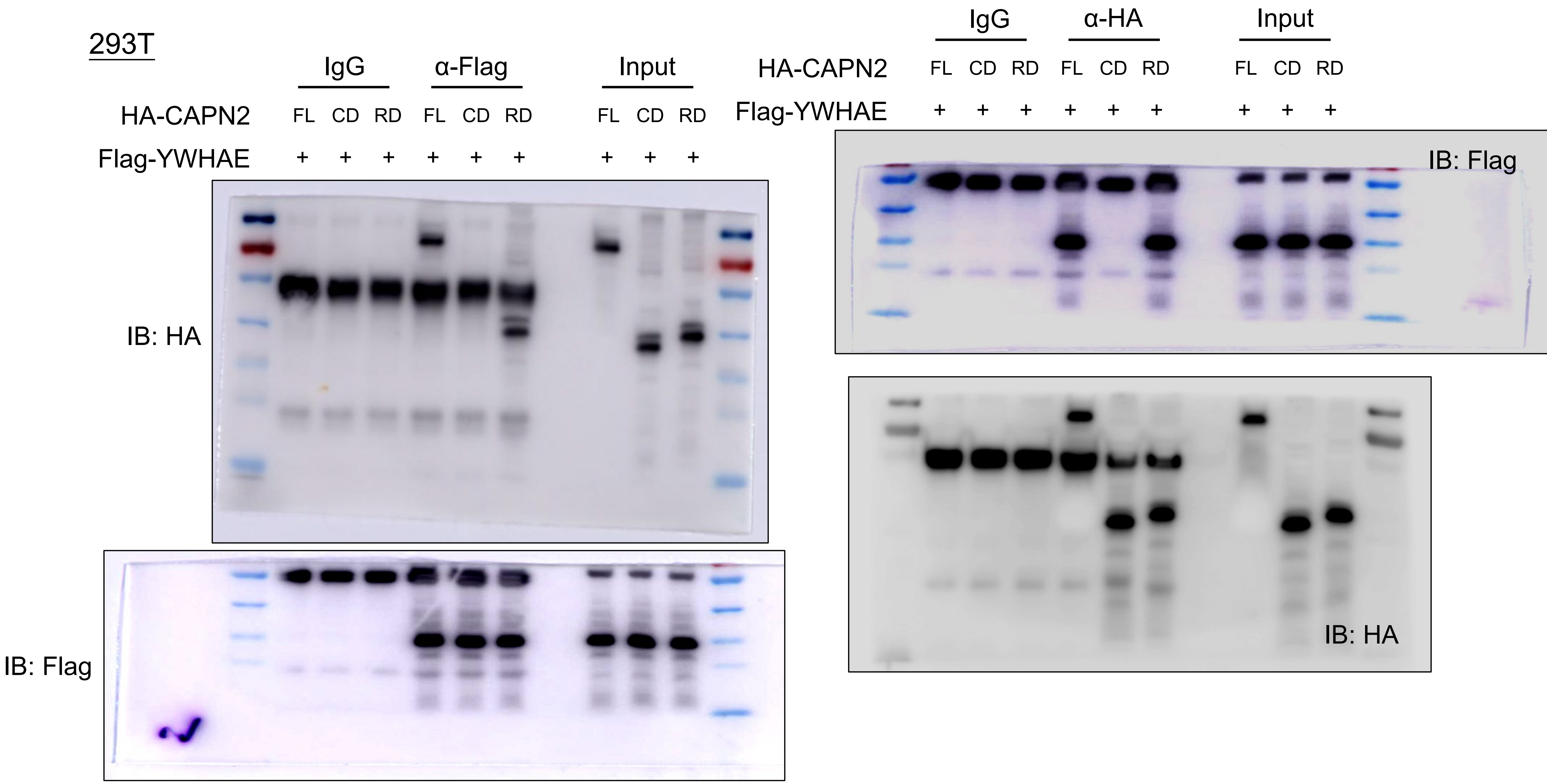

293T
IgG
α-Flag
Input
HA-CAPN2
FL CD RD FL CD RD FL CD RD
Flag-YWHAE
+ + + + + + + + +
IB: HA
IB: Flag
IgG
α-HA
Input
HA-CAPN2
FL CD RD FL CD RD FL CD RD
Flag-YWHAE
+ + + + + + + + +
IB: Flag
IB: HA

Fig. 6j

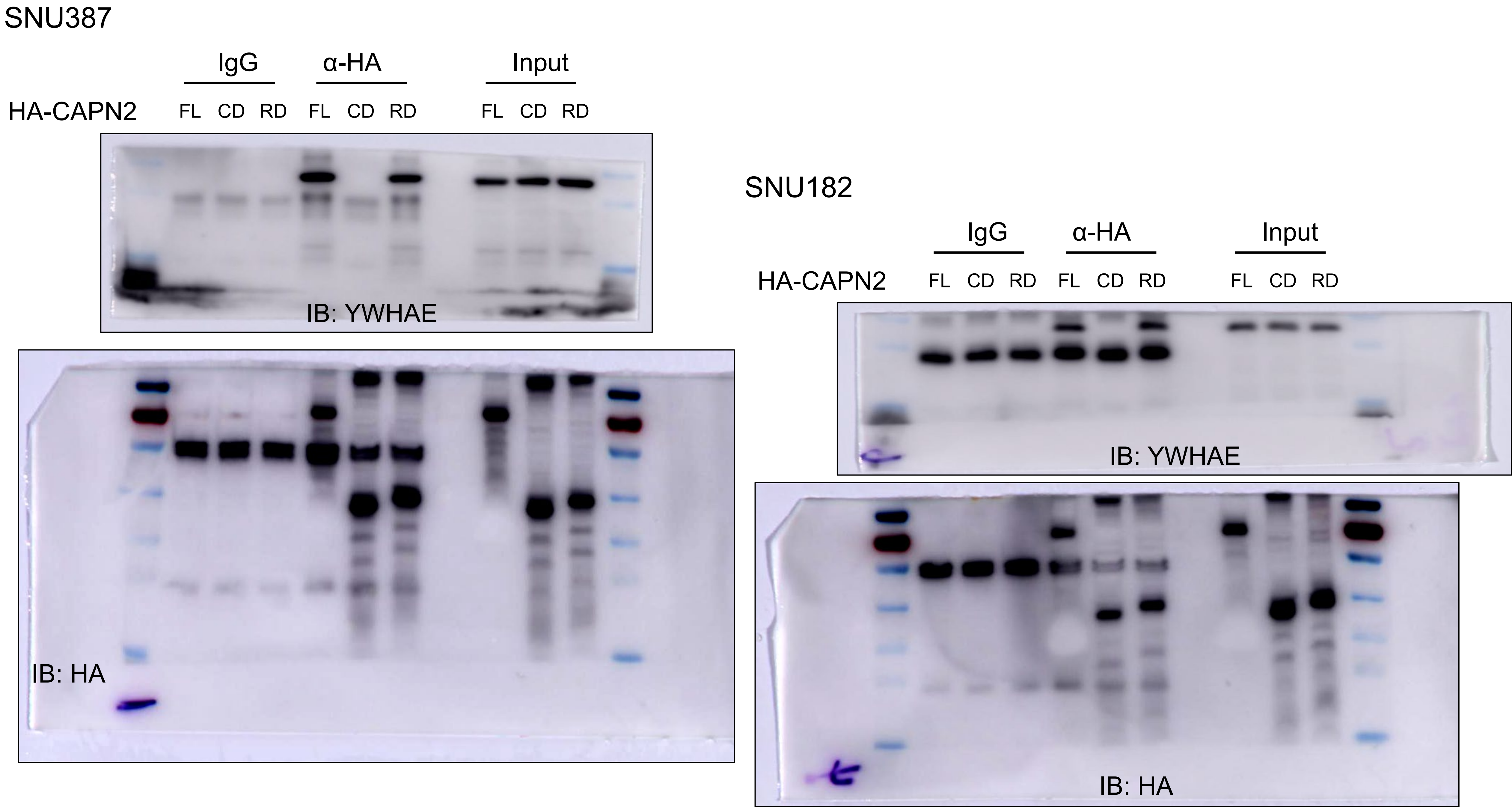
SNU387
IgG
α-HA
Input
HA-CAPN2
FL CD RD FL CD RD FL CD RD
IB: YWHAE
IB: HA
SNU182
IgG
α-HA
Input
HA-CAPN2
FL CD RD FL CD RD FL CD RD
IB: YWHAE
IB: HA

Fig. 7c

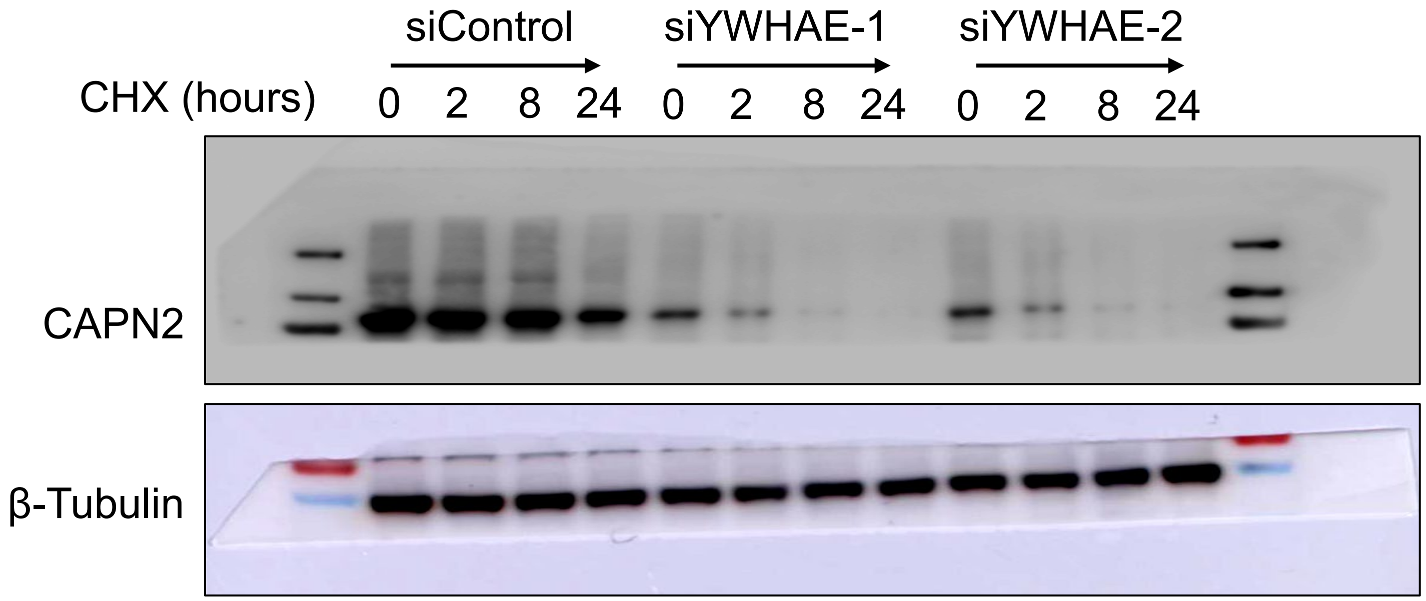
siControl
siYWHAE-1
siYWHAE-2
CHX (hours)
0 2 8 24 0 2 8 24 0 2 8 24
CAPN2
β-Tubulin

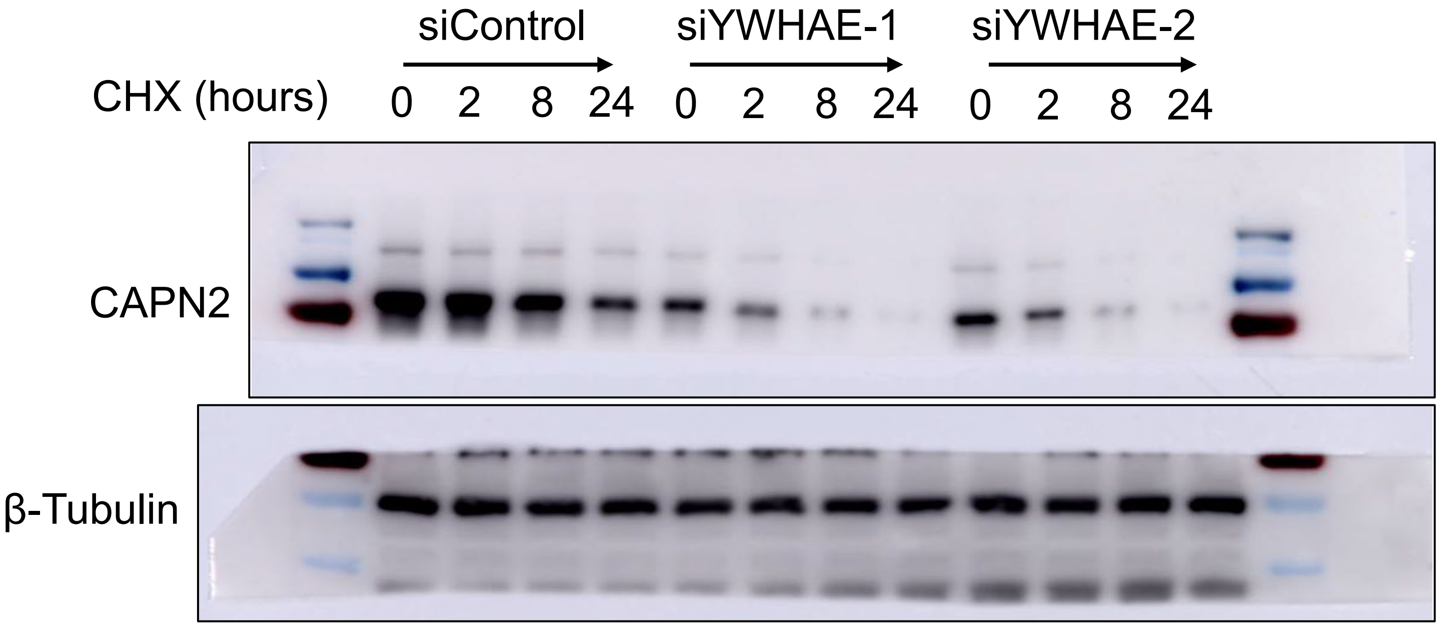
siControl
siYWHAE-1
siYWHAE-2
CHX (hours)
0 2 8 24 0 2 8 24 0 2 8 24
CAPN2
β-Tubulin

Fig. 7d

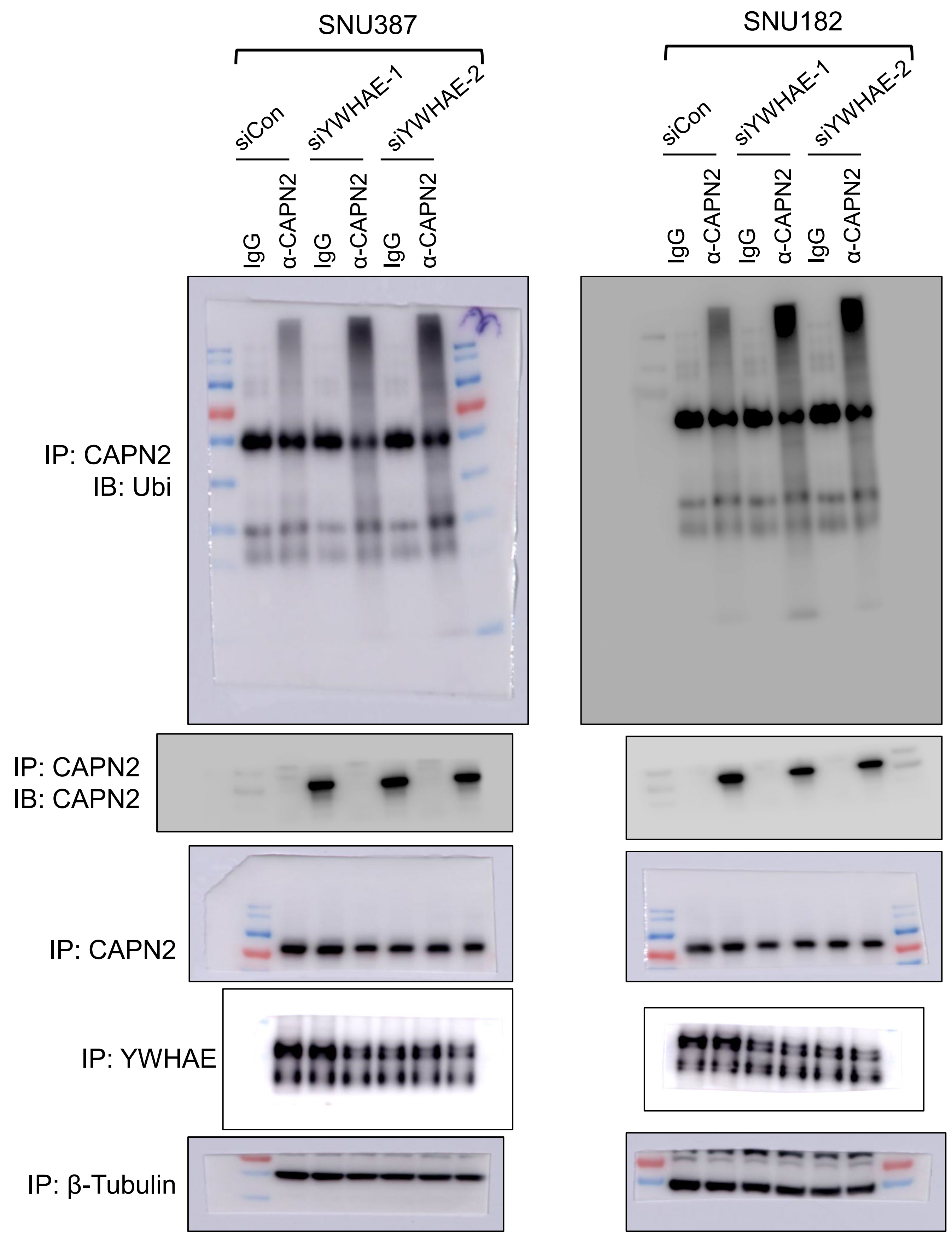
SNU387
SNU182
siCon
siYWHAE-1
siYWHAE-2
IgG
α-CAPN2
IP: CAPN2
IB: Ubi
IP: CAPN2
IB: CAPN2
IP: CAPN2
IP: YWHAE
IP: β-Tubulin

Fig. 7f

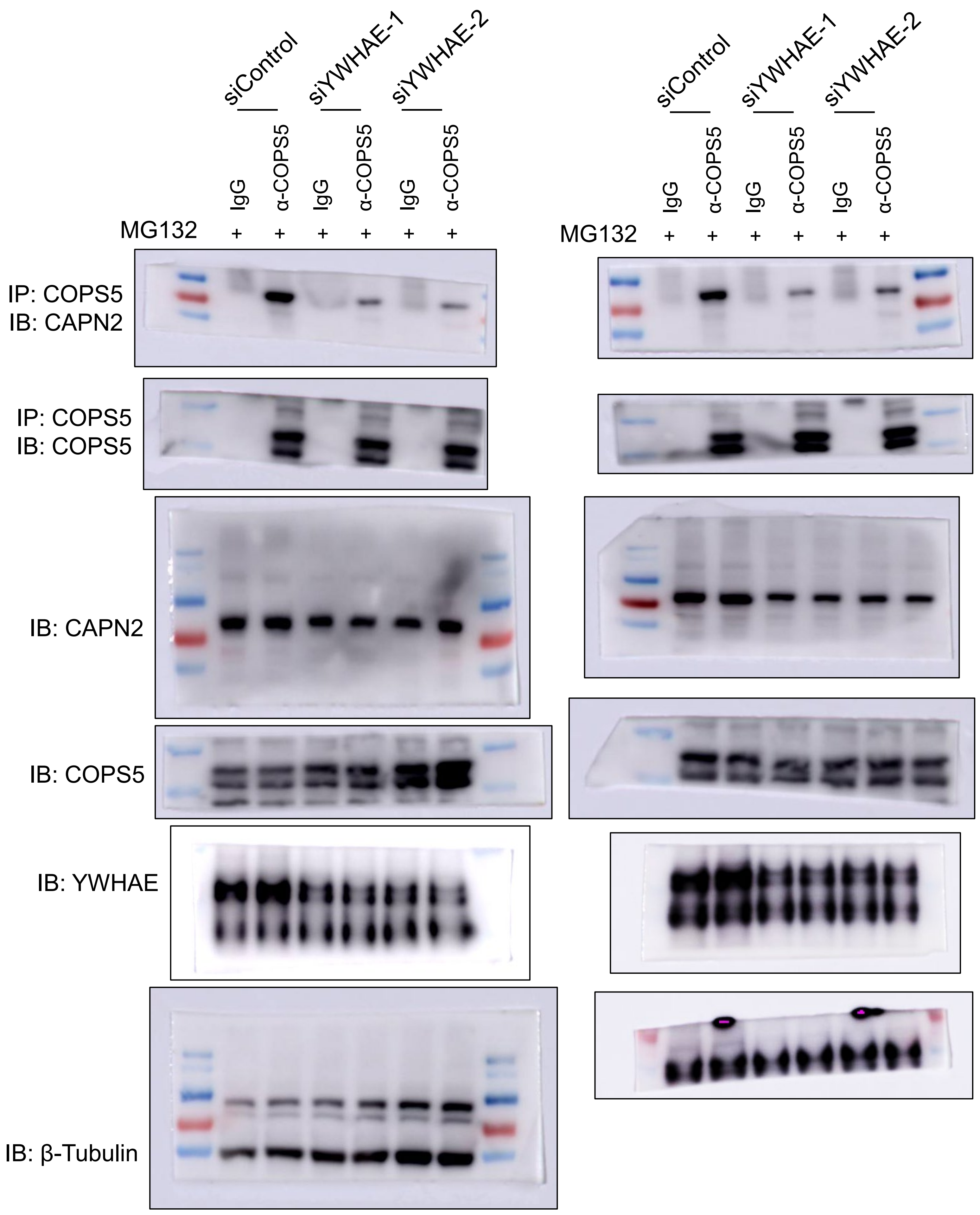
siControl
siYWHAE-1
siYWHAE-2
IgG
α-COPS5
MG132
+
IP: COPS5
IB: CAPN2
IP: COPS5
IB: COPS5
IB: CAPN2
IB: COPS5
IB: YWHAE
IB: β-Tubulin

Fig. 7g

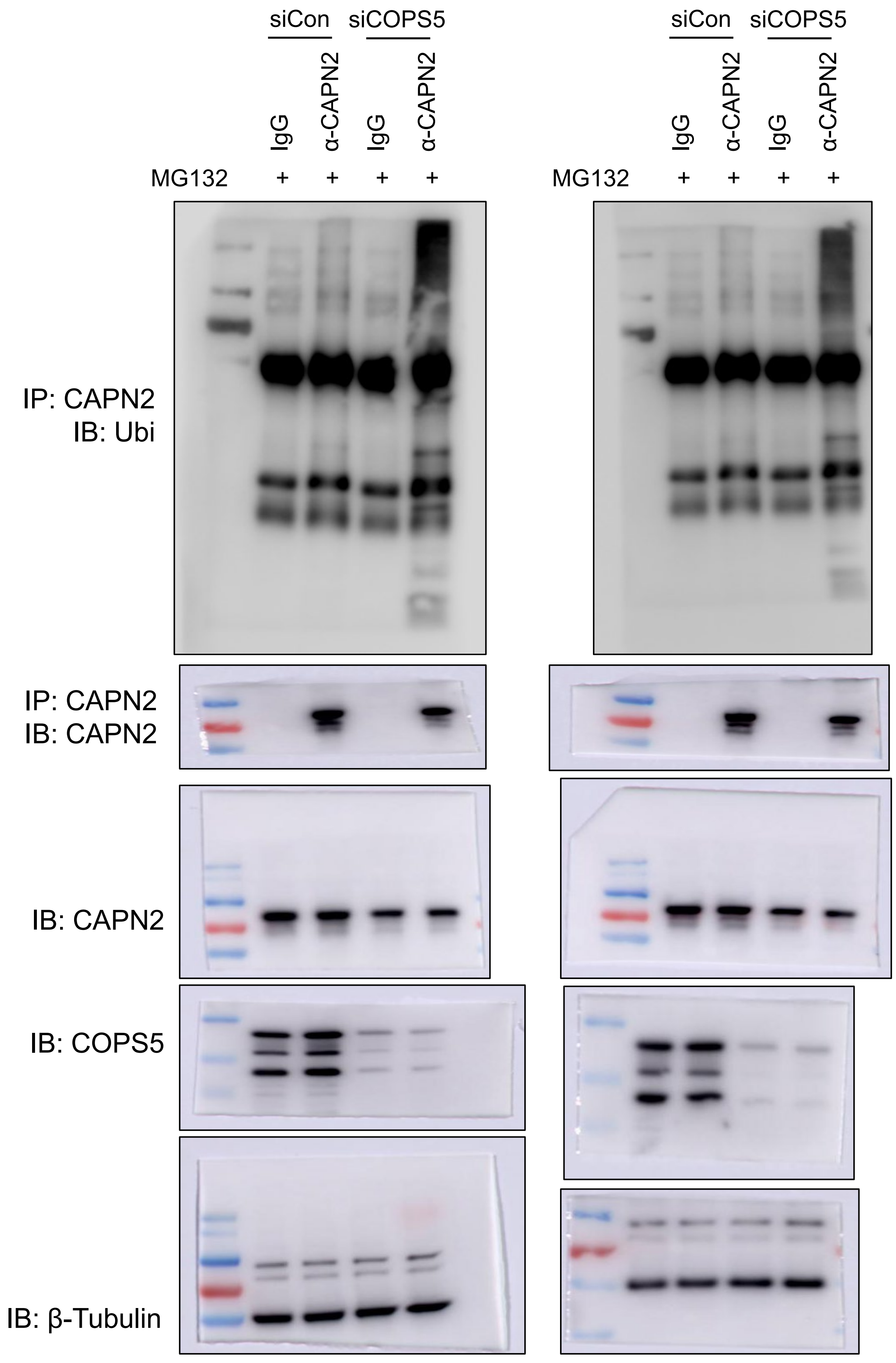
siCon
siCOPS5
IgG
α-CAPN2
IgG
α-CAPN2
MG132
+ + + +
IP: CAPN2
IB: Ubi
IP: CAPN2
IB: CAPN2
IB: CAPN2
IB: COPS5
IB: β-Tubulin

Fig. 7h

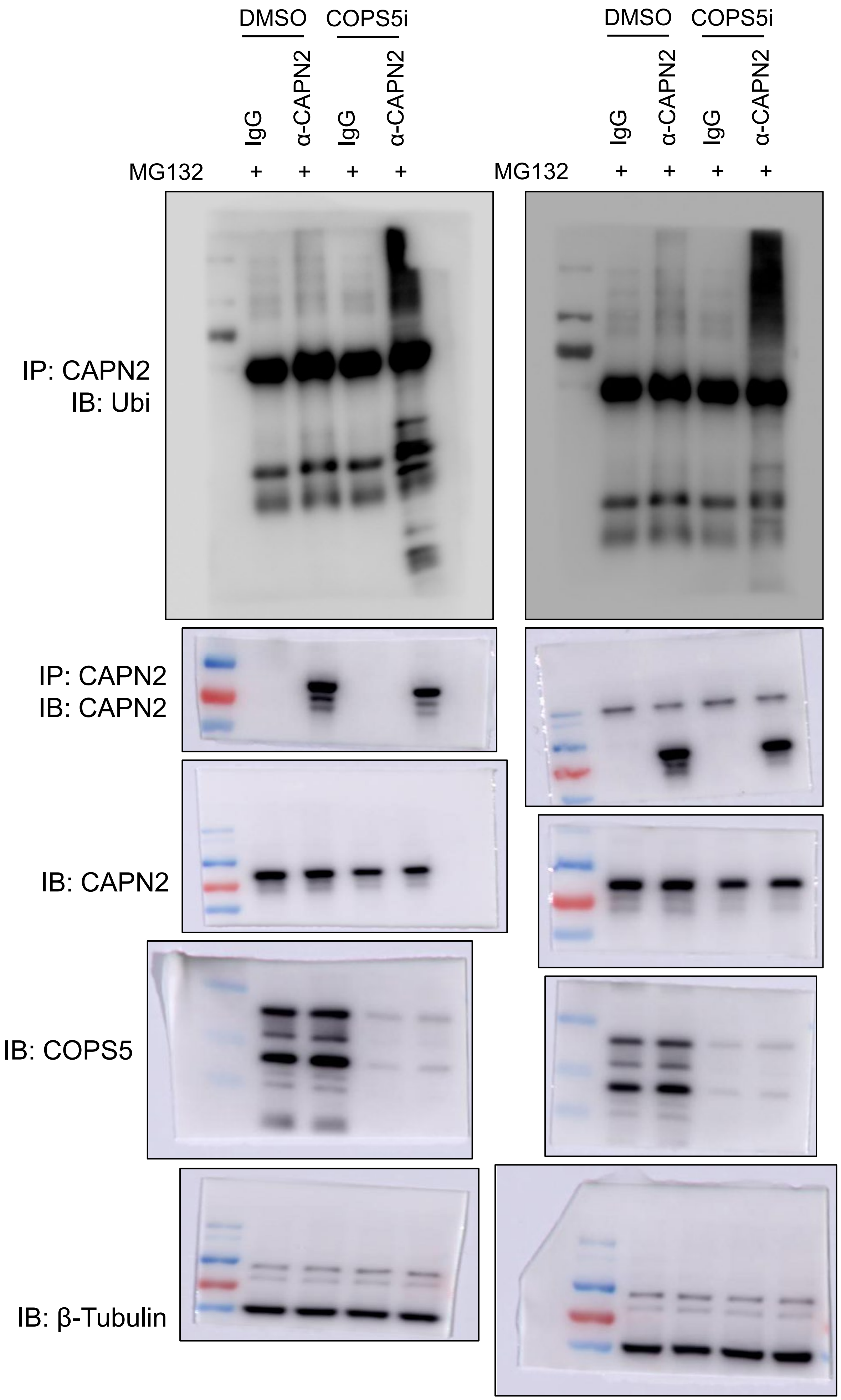
DMSO
COPS5i
IgG
α-CAPN2
IgG
α-CAPN2
MG132
+ + + +
IP: CAPN2
IB: Ubi
IP: CAPN2
IB: CAPN2
IB: CAPN2
IB: COPS5
IB: β-Tubulin
